# Assessment of Global Trends in the Diagnosis of Mesothelioma From 1990 to 2017

**DOI:** 10.1001/jamanetworkopen.2021.20360

**Published:** 2021-08-11

**Authors:** Zhen Zhai, Jian Ruan, Yi Zheng, Dong Xiang, Na Li, Jingjing Hu, Jianfei Shen, Yujiao Deng, Jia Yao, Peng Zhao, Shuqian Wang, Si Yang, Linghui Zhou, Ying Wu, Peng Xu, Lijuan Lyu, Jun Lyu, Raymond Bergan, Tianhui Chen, Zhijun Dai

**Affiliations:** 1 Department of Breast Surgery, The First Affiliated Hospital, College of Medicine, Zhejiang University, Hangzhou, China; 2 Department of Oncology, The Second Affiliated Hospital of Xi’an Jiaotong University, Xi’an, China; 3 Department of Medical Oncology, The First Affiliated Hospital, College of Medicine, Zhejiang University, Hangzhou, China; 4 Celilo Cancer Center, Oregon Health Science Center Affiliated Mid-Columbia Medical Center, The Dalles, Oregon; 5 Dana-Farber Cancer Institute, Harvard Medical School, Boston, Massachusetts; 6 Department of Cardiothoracic Surgery, Taizhou Hospital of Zhejiang Province, Wenzhou Medical University, Linhai, China; 7 Department of Clinical Research, The First Affiliated Hospital of Jinan University, Guangzhou, China; 8 Knight Cancer Institute, Oregon Health and Science University, Portland; 9 Department of Cancer Prevention/Experimental Research Center, Cancer Hospital of the University of Chinese Academy of Sciences (Zhejiang Cancer Hospital), Hangzhou, China; 10 Institute of Basic Medicine and Cancer, Chinese Academy of Sciences, Hangzhou, China

## Abstract

**Importance:**

It is difficult for policy makers and clinicians to formulate targeted management strategies for mesothelioma because data on current epidemiological patterns worldwide are lacking.

**Objective:**

To evaluate the mesothelioma burden across the world and describe its epidemiological distribution over time and by sociodemographic index (SDI) level, geographic location, sex, and age.

**Design, Setting, and Participants:**

Annual case data and age-standardized rates of incidence, death, and disability-adjusted life-years associated with mesothelioma among different age groups were obtained from the Global Burden of Disease 2017 database. The estimated annual percentage changes in age-standardized rates were calculated to evaluate temporal trends in incidence and mortality. The study population comprised individuals from 21 regions in 195 countries and territories who were diagnosed with mesothelioma between 1990 and 2017. Data were collected from May 23, 2019, to January 18, 2020.

**Main Outcomes and Measures:**

Primary outcomes were incident cases, deaths, and their age-standardized rates and estimated annual percentage changes. Secondary outcomes were disability-adjusted life-years and relative temporal trends.

**Results:**

Overall, 34 615 new cases (95% uncertainty interval [UI], 33 530-35 697 cases) of mesothelioma and 29 909 deaths (95% UI, 29 134-30 613 deaths) associated with mesothelioma were identified in 2017, and more than 70% of these cases and deaths were among male individuals. In 1990, the number of incident cases was 21 224 (95% UI, 17 503-25 450), and the number of deaths associated with mesothelioma was 17 406 (95% UI, 14 495-20 660). These numbers increased worldwide from 1990 to 2017, with more than 50% of cases recorded in regions with high SDI levels, whereas the age-standardized incidence rate (from 0.52 [95% UI, 0.43-0.62] in 1990 to 0.44 [95% UI, 0.42-0.45] in 2017) and the age-standardized death rate (from 0.44 [95% UI, 0.37-0.52] in 1990 to 0.38 [95% UI, 0.37-0.39] in 2017) decreased, with estimated annual percentage changes of –0.61 (95% CI, –0.67 to –0.54) for age-standardized incidence rate and −0.44 (95% CI, –0.52 to –0.37) for age-standardized death rate. The proportion of incident cases among those 70 years or older continued to increase (from 36.49% in 1990 to 44.67% in 2017), but the proportion of patients younger than 50 years decreased (from 16.74% in 1990 to 13.75% in 2017) over time. In addition, mesothelioma incident cases and age-standardized incidence rates began to decrease after 20 years of a complete ban on asbestos use. For example, in Italy, a complete ban on asbestos went into effect in 1992; incident cases increased from 1409 individuals (95% UI, 1013-1733 individuals) in 1990, peaked in 2015 after 23 years of the asbestos ban, then decreased from 1820 individuals (95% UI, 1699-1981 individuals) in 2015 to 1746 individuals (95% UI, 1555-1955 individuals) in 2017.

**Conclusions and Relevance:**

This cross-sectional study found that incident cases of mesothelioma and deaths associated with mesothelioma continuously increased worldwide, especially in resource-limited regions with low SDI levels. Based on these findings, global governments and medical institutions may consider formulating optimal policies and strategies for the targeted prevention and management of mesothelioma.

## Key Points

**Question:**

What are the temporal trends and epidemiological distribution of mesothelioma, and have cases of mesothelioma increased worldwide since 1990?

**Findings:**

In this cross-sectional study of 21 regions comprising 195 countries and territories, global mesothelioma cases continuously increased, with more than 50% of cases recorded in regions with high sociodemographic index levels; in recent years, incidence and mortality also increased, especially among female individuals, in regions with low sociodemographic index levels.

**Meaning:**

This study found that incident mesothelioma cases and deaths associated with mesothelioma have continuously increased worldwide, especially in resource-limited regions in which a complete and immediate ban on asbestos use may be warranted.

## Teaser

This cross-sectional study uses data from the Global Burden of Disease Study 2017 to evaluate the temporal trends and epidemiological distribution of mesothelioma worldwide from 1990 to 2017.

## Introduction

Mesothelioma is a rare disease that accounted for 30 443 new cancer cases (0.2%) and 25 576 cancer deaths (0.3%) worldwide in 2018.^1^ Long-term exposure to asbestos during activities such as mining, shipbuilding, and construction is a well-established risk factor for mesothelioma.^2,3,4^ In addition, germline *BAP1* variations and radiotherapy may also be associated with the development of mesothelioma.^5,6,7^ Male individuals have higher incidence and mortality rates for mesothelioma compared with female individuals, and mesothelioma is commonly diagnosed at older ages because of its long latent period (approximately 40 years).^1,8,9^

The incidence of mesothelioma has decreased in countries, such as Sweden, that have implemented a complete ban on asbestos use for more than 2 decades.^10^ Although a complete ban on asbestos use has been implemented in some non–resource-limited countries, resource-limited countries, such as Brazil, Russia, India, and China, are still using large amounts of chrysotile asbestos.^11,12,13,14,15^ Although the Global Burden of Disease (GBD) 2016 Occupational Carcinogens Collaborators^16^ investigated the cancer burden associated with occupational exposure to asbestos (eMethods in the Supplement), estimations of the global burden and temporal trends for mesothelioma are scant, which makes it difficult for global policy makers to form a targeted management strategy from a global perspective. Therefore, we aimed to investigate the global burden of mesothelioma overall and stratified by sociodemographic index (SDI) level, geographic location, sex, age, and change over time, which may provide a basis for optimizing strategies for the global management of mesothelioma and may help to support the call for a complete ban on asbestos use (rather than restricted use) in resource-limited countries.

## Methods

Using the Global Health Data Exchange (GHDx) online query tool,^17^ we extracted annual case data and age-standardized rates (ASRs) of mesothelioma incidence, deaths, and disability-adjusted life-years (DALYs) among individuals who received a diagnosis of mesothelioma from 1990 to 2017 (eMethods in the Supplement). Data were collected from May 23, 2019, to January 18, 2020. The study was approved by the ethics committee of the First Affiliated Hospital of School of Medicine, Zhejiang University, China, with a waiver of informed consent because all data from the GHDx are deidentified. This study followed the Strengthening the Reporting of Observational Studies in Epidemiology (STROBE) reporting guideline for cross-sectional studies.

Uncertainty intervals (UIs) of numbers and rates were calculated to reflect the certainty of estimates (eMethods in the Supplement). In the Global Burden of Disease Study 2017 (GBD 2017),^17^ every estimate was calculated 1000 times; each time estimates were calculated, they were sampled from distributions rather than point estimates for data inputs, data transformations, and model choice. The 95% UI was determined by the 25th and 975th value of the 1000 values after ordering them from smallest to largest. The data were derived from 21 regions comprising 195 countries and territories. Patients were categorized into 3 age groups (15-49 years, 50-69 years, and >69 years). Patients younger than 15 years were excluded because no data for this age group were available. Data were grouped into 21 different regions according to geographic location and into 5 SDI levels according to the socioeconomic status of the countries and territories. The SDI was developed by GBD researchers and is a composite indicator constructed from measures of per capita income, average years of education, and total fertility rates.^17^ Mesothelioma incidence was estimated using individual or integrated databases of cancer registries, such as the Nordic Cancer Statistics; Cancer Incidence in Five Continents; and Surveillance, Epidemiology, and End Results registries.

The ASRs and their estimated annual percentage changes (EAPCs) were calculated to evaluate the incidence and mortality trends of mesothelioma. Disability-adjusted life-years were estimated by adding the years lived with disability and the years of life lost.^18^ Temporal trends in ASRs were reflected by the EAPC value, which was approximately equal to the annual change in a specified range and was calculated on a linear scale.^19^ In the linear regression model, the natural logarithm of ASR was calculated as the constant in the initial calendar year plus the annual change in rates per 100 000 people in a given calendar year plus the error term. The EAPC was calculated as 100 multiplied by the product of the exponential function (based on the constant) and the annual change in rates per 100 000 people minus 1. We also calculated 95% CIs for EAPCs. When the estimated value and lower 95% CI of the EAPC were positive, the ASRs were considered to be increasing; when the estimated value and upper 95% CI of the EAPC were negative, the ASRs were considered to be decreasing. Otherwise, the ASR was considered to be stable.

In addition, we explored the association between the time since the complete asbestos ban was enacted and the temporal trend of mesothelioma incidence using information about the chronology of global asbestos bans and restrictions issued by the International Ban Asbestos Secretariat.^20^ We included 47 countries with a complete ban on asbestos use (including chrysotile) (eMethods and eTable 1 in the Supplement), and we calculated temporal trends of mesothelioma incidence after the complete ban was enacted in those 47 countries.

### Statistical Analysis

Most of the results of this study were descriptive. Statistical analysis was performed to calculate the correlations of EAPC with mesothelioma ASR and SDI level and to describe the temporal trends in ASR using a joinpoint regression model. We estimated the association of EAPC with ASR in 1990 to ascertain the baseline burden of mesothelioma, and the association of EAPC with SDI level in 2017 was used as a surrogate for the current SDI level of each national health care system. The population correlation coefficient (ρ index) and *P* value were derived using Pearson correlation analysis. All analyses were performed using R software, version 3.5.3 (R Foundation for Statistical Computing). The joinpoint regression model was used to calculate temporal trends in incidence and mortality rates (eMethods in the Supplement). The EAPC between successive joinpoints and best-fitting points (ie, the points at which statistically significant changes occurred) were calculated using the Joinpoint Regression Program, version 4.7.0.0 (Surveillance Research Program, National Cancer Institute), with 2-sided *P* < .05 indicating a statistically significant difference.

## Results

### Global Burden of Mesothelioma

A total of 34 615 new cases (95% UI, 33 530-35 697 cases) of mesothelioma and 29 909 deaths (95% UI, 29 134-30 613 deaths) associated with mesothelioma were identified globally in 2017 (Table; eTable 2 in the Supplement); more than 70% of cases and deaths were among male individuals. From 1990 to 2017, the number of incident cases, deaths, and DALYs increased substantially from 1990 to 2017 (incident cases: 21 224 [95% UI, 17 503-25 450] to 34 615 [95% UI, 33 530-35 697]; deaths: 17 406 [95% UI, 14 495-20 660] to 29 909 [95% UI, 29 134-30 613]; DALYs: 431 940 [95% UI, 352 210-527 020] to 670 690 [95% UI, 648 200-693 050]), and these increases were found only in those older than 50 years (eTable 2, eTable 3, and eFigure 1 in the Supplement). Among those younger than 50 years, the number of new cases remained stable in regions with high SDI levels but continued to increase in regions with low SDI levels (from 344 cases [95% UI, 168-644 cases] in 1990 to 582 cases [95% UI, 385-907 cases] in 2017). Among the SDI quintiles, countries with higher SDI levels had more incident cases, deaths, and DALYs compared with countries with low SDI levels, with 54.7% of new cases and 57.5% of deaths occurring in countries with high SDI levels in 2017 (Table; eTable 2 and eFigure 1 in the Supplement).

**Table.** Incident Cases, Age-Standardized Incidence Rates, and Temporal Trends for Mesothelioma From 1990 to 2017

Variable	No. (95% UI)						No. (95% CI)	
	1990			2017			1990-2017	
	Incident cases, ×10^2^	ASIR per 100 000 people	Incident cases, ×10^2^		ASIR per 100 000 people	EAPC	
SDI							
High	129.98 (108.66 to 146.28)	1.00 (0.83 to 1.12)	189.60 (182.05 to 198.31)		0.85 (0.82 to 0.89)	–0.52 (–0.56 to –0.48)	
Middle to high	31.94 (27.04 to 39.46)	0.32 (0.27 to 0.40)	53.81 (50.38 to 56.53)		0.30 (0.28 to 0.31)	–0.29 (–0.47 to –0.10)	
Middle	23.89 (20.60 to 29.23)	0.22 (0.19 to 0.27)	51.43 (48.88 to 53.95)		0.23 (0.22 to 0.24)	–0.12 (–0.27 to 0.03)	
Low to middle	16.66 (11.11 to 26.27)	0.26 (0.18 to 0.39)	33.22 (28.85 to 39.29)		0.26 (0.23 to 0.31)	0.05 (0.01 to 0.08)	
Low	9.40 (5.39 to 16.75)	0.25 (0.15 to 0.43)	17.36 (13.06 to 25.17)		0.22 (0.17 to 0.32)	–0.45 (–0.65 to –0.25)	
Global	212.24 (175.03 to 254.50)	0.52 (0.43 to 0.62)	346.15 (335.30 to 356.97)		0.44 (0.42 to 0.45)	–0.61 (–0.67 to –0.54)	
Regional							
Andean Latin America	1.73 (1.41 to 2.16)	0.77 (0.63 to 0.96)	1.48 (1.32 to 1.66)		0.27 (0.24 to 0.30)	–5.97 (–6.94 to –4.99)	
Australasia	5.51 (4.11 to 6.48)	2.26 (1.69 to 2.65)	0.65 (9.14 to 12.43)		2.13 (1.84 to 2.47)	0 (–0.17 to 0.17)	
Caribbean	0.75 (0.56 to 1.02)	0.27 (0.20 to 0.36)	1.12 (0.96 to 1.34)		0.22 (0.19 to 0.26)	–0.91 (–1.04 to –0.79)	
Central Asia	1.25 (1.12 to 1.45)	0.24 (0.21 to 0.28)	2.06 (1.93 to 2.21)		0.26 (0.25 to 0.28)	–0.03 (–0.45 to 0.39)	
Central Europe	4.27 (3.82 to 4.90)	0.28 (0.25 to 0.33)	8.27 (7.66 to 8.98)		0.42 (0.38 to 0.45)	1.87 (1.72 to 2.02)	
Central Latin America	2.73 (2.38 to 3.13)	0.28 (0.25 to 0.32)	7.23 (6.86 to 7.61)		0.30 (0.29 to 0.32)	0.41 (0.24 to 0.57)	
Central sub-Saharan Africa	0.65 (0.39 to 1.12)	0.24 (0.15 to 0.40)	1.16 (0.86 to 1.70)		0.20 (0.15 to 0.28)	–0.99 (–1.14 to –0.84)	
East Asia	13.81 (10.57 to 18.27)	0.14 (0.11 to 0.18)	30.55 (28.30 to 32.33)		0.15 (0.14 to 0.16)	0.87 (0.44 to 1.31)	
Eastern Europe	9.51 (8.04 to 11.69)	0.33 (0.28 to 0.41)	12.33 (11.48 to 13.40)		0.38 (0.35 to 0.41)	0 (–0.43 to 0.43)	
Eastern sub-Saharan Africa	2.25 (1.21 to 4.17)	0.25 (0.13 to 0.45)	2.87 (1.91 to 3.93)		0.16 (0.11 to 0.21)	–2.18 (–2.43 to –1.92)	
High-income Asia Pacific	7.04 (6.13 to 7.73)	0.34 (0.30 to 0.38)	16.98 (15.94 to 18.44)		0.39 (0.37 to 0.43)	1.02 (0.78 to 1.26)	
High-income North America	28.51 (25.23 to 30.94)	0.78 (0.69 to 0.84)	38.15 (36.64 to 39.66)		0.62 (0.59 to 0.64)	–1.09 (–1.21 to –0.98)	
North Africa and Middle East	10.61 (6.16 to 16.42)	0.54 (0.32 to 0.83)	20.94 (18.09 to 23.90)		0.47 (0.41 to 0.52)	–0.38 (–0.69 to –0.07)	
Oceania	0.13 (0.08 to 0.21)	0.34 (0.23 to 0.55)	0.32 (0.23 to 0.51)		0.40 (0.30 to 0.60)	0.94 (0.80 to 1.09)	
South Asia	13.76 (8.66 to 23.84)	0.21 (0.13 to 0.35)	34.74 (29.26 to 44.52)		0.25 (0.21 to 0.31)	0.62 (0.45 to 0.78)	
Southeast Asia	11.89 (8.37 to 17.85)	0.37 (0.27 to 0.53)	17.85 (16.23 to 19.67)		0.28 (0.26 to 0.31)	–1.36 (–1.57 to –1.15)	
Southern Latin America	1.50 (1.34 to 1.70)	0.32 (0.28 to 0.36)	3.46 (3.10 to 3.90)		0.43 (0.39 to 0.49)	1.78 (1.49 to 2.08)	
Southern sub-Saharan Africa	1.98 (1.67 to 2.33)	0.67 (0.56 to 0.80)	4.00 (3.61 to 4.51)		0.71 (0.65 to 0.80)	0.06 (–0.67 to 0.79)	
Tropical Latin America	5.52 (4.89 to 6.86)	0.55 (0.49 to 0.69)	10.91 (10.15 to 11.60)		0.46 (0.43 to 0.49)	–0.31 (–0.56 to –0.07)	
Western Europe	86.20 (70.15 to 98.33)	1.46 (1.19 to 1.66)	117.29 (111.38 to 123.69)		1.31 (1.24 to 1.38)	–0.34 (–0.42 to –0.26)	
Western sub-Saharan Africa	2.65 (1.79 to 3.46)	0.26 (0.18 to 0.33)	3.78 (3.10 to 4.57)		0.18 (0.15 to 0.21)	–1.99 (–2.23 to –1.76)	

Abbreviations: ASIR, age-standardized incidence rate; EAPC, estimated annual percentage change; SDI, sociodemographic index; UI, uncertainty interval.

**Figure 1.**
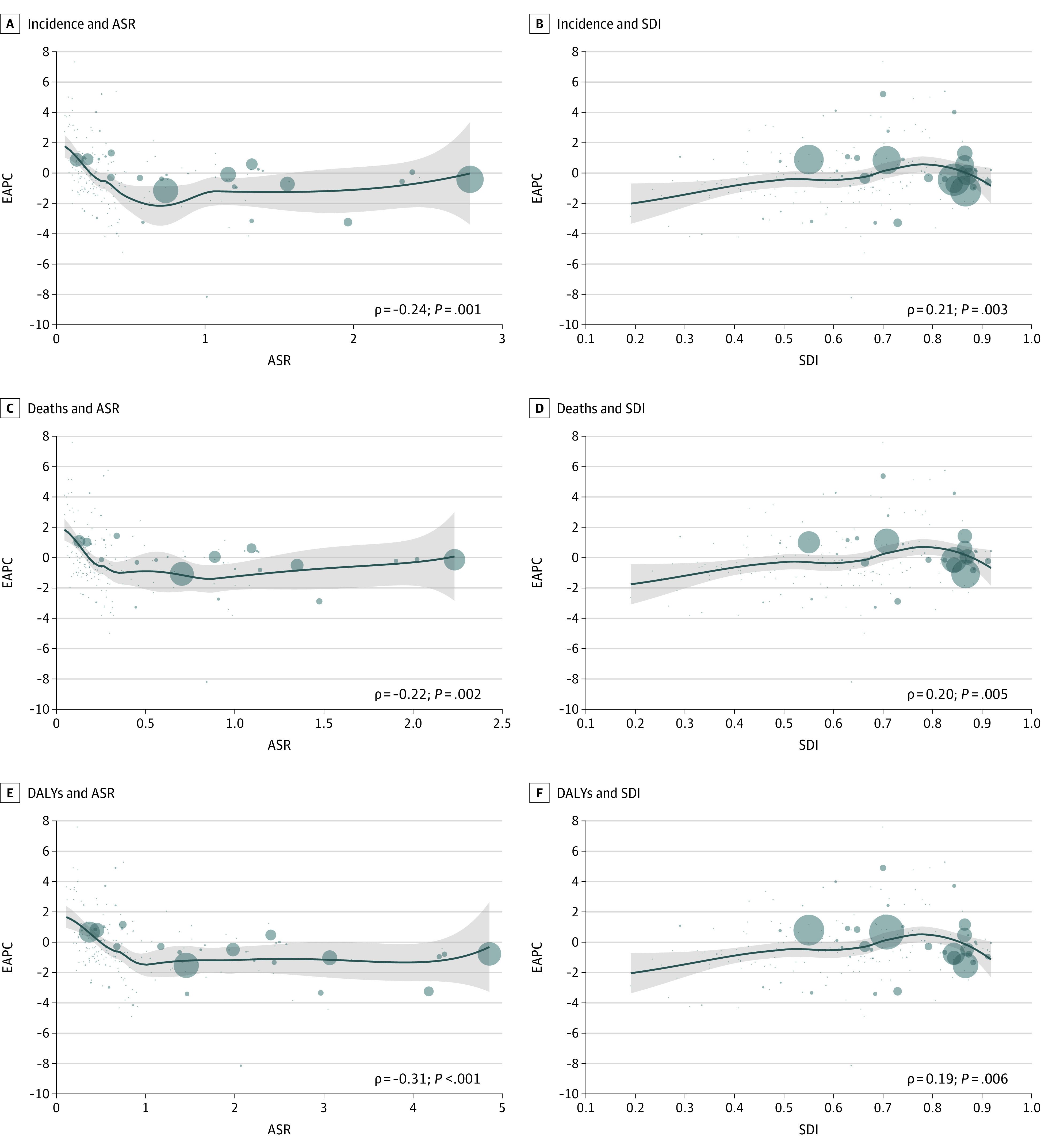
Correlation of Estimated Annual Percentage Change (EAPC) in Mesothelioma Incidence, Deaths, and Disability-Adjusted Life-Years (DALYs) With Age-Standardized Rate of Mesothelioma in 1990 and Sociodemographic Index in 2017 The ρ indices and *P* values were derived using Pearson correlation analysis. Circles represent cases or deaths in countries and territories with available SDI data. Larger circles represent a greater number of cases or deaths. Incidence and deaths were calculated based on the number of mesothelioma cases per 200 people, and DALYs were calculated based on the number of mesothelioma cases per 4000 people.

Sociodemographic index levels were positively correlated with age-standardized incidence rates (ASIRs) and age-standardized death rates (ASDRs) (eFigure 2 and eFigure 3 in the Supplement). With regard to temporal trends in incidence, the EAPC of the ASIR was negatively correlated with the ASIR in 1990 (ρ = −0.24; *P* = .001), and this trend was more distinct in countries with ASIRs lower than 0.7 per 100 000 people. In contrast, the EAPC of the ASIR was positively correlated with the SDI level in 2017 (ρ = 0.21; *P* = .003). However, the correlation between EAPCs and SDI levels was reversed in countries with SDI levels greater than 0.8; these countries had a high mesothelioma burden. Similar results were also observed for the EAPCs of ASDRs and the ASRs of DALYs (Figure 1).

The ASIR, ASDR, and ASR of DALYs decreased from 1990 (ASIR, 0.52 [95% UI, 0.43-0.62]; ASDR, 0.44 [95% UI, 0.37-0.52]; ASR of DALYs, 10.03 [95% UI, 8.25-12.14]) to 2017 (ASIR, 0.44 [95% UI, 0.42-0.45]; ASDR, 0.38 [95% UI, 0.37-0.39]; ASR of DALYs, 8.31 [95% UI, 8.03-8.58]), with EAPCs of –0.61 (95% CI, –0.67 to –0.54) for ASIR, –0.44 (95% CI, –0.52 to –0.37) for ASDR, and –0.71 (95% CI, –0.76 to –0.65) for the ASR of DALYs (Table; eTable 2 and eTable 3 in the Supplement). A total of 86.3% of patients with mesothelioma worldwide were 50 years or older. The global proportion of patients older than 70 years increased from 36.5% in 1990 to 44.7% in 2017, and the proportion of patients (particularly female patients) younger than 50 years decreased substantially (from 16.7% in 1990 to 13.8% in 2017), with the proportion of female patients younger than 50 years decreasing from 26.7% in 1990 to 20.9% in 2017 (eFigure 4 and eFigure 5 in the Supplement). Incident cases and deaths among both female individuals (incident cases: from 7175 [95% UI, 5494-9269] in 1990 to 10 038 [95% UI, 9548-10 685] in 2017; deaths: from 5421 [95% UI, 4254-6883] in 1990 to 8176 [95% UI, 7878-8573] in 2017) and male individuals (incident cases: from 14 049 [95% UI, 11 825-16 475] in 1990 to 24 577 [95% UI, 23 696-25 501] in 2017; deaths: from 11 985 [95% UI, 10 163-13 968] in 1990 to 21 732 [95% UI, 21 028-22 471] in 2017) increased globally, especially in regions with low to middle SDI levels (eFigure 6 and eFigure 7 in the Supplement). In addition, among those older than 44 years, the rates of incidence, death, and DALYs were higher among male individuals compared with female individuals in both 1990 and 2017; however, among those younger than 45 years, female individuals had higher incidence and death rates than male individuals (eFigures 8-10 in the Supplement).

**Figure 2.**
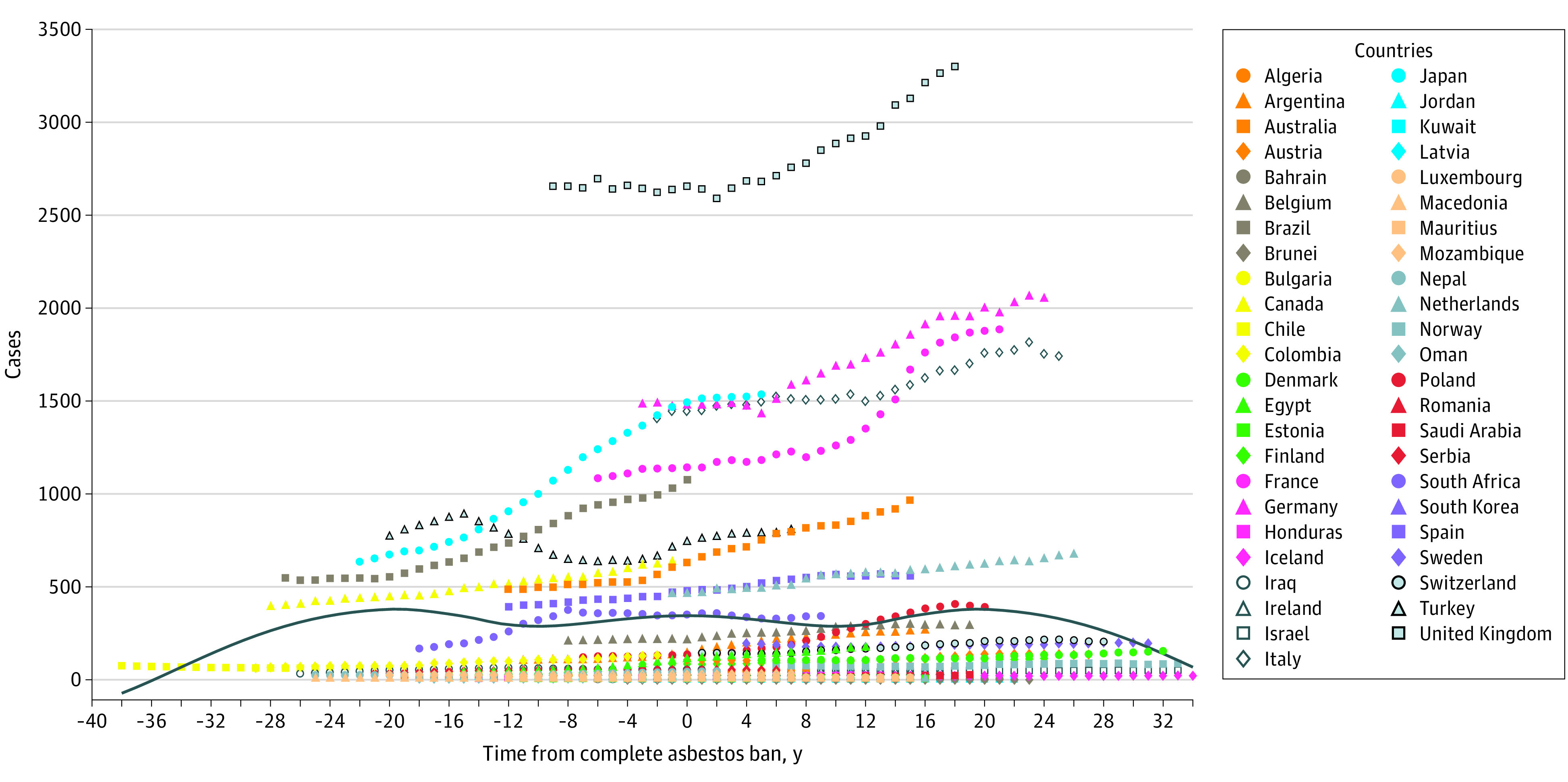
Temporal Trends in Mesothelioma Cases Among 47 Countries With Complete Asbestos Bans The solid blue curve reflects the overall trend of incident cases.

An analysis of temporal trends in mesothelioma incident cases in the 47 countries with complete asbestos bans from 1990 to 2017 revealed that mesothelioma incidence began to decrease after 20 years of a complete ban on asbestos use (Figure 2). A slight increase in ASIR occurred after a complete ban, followed by a distinct decrease after almost 3 decades. For example, from 1990 to 2017, ASIR decreased from 1.04 (95% UI, 0.67-1.31) to 0.66 (95% UI, 0.57-0.77) in the United Kingdom (UK), from 0.74 (95% UI, 0.64-0.87) to 0.77 (95% UI, 0.69-0.87) in South Africa, and from 2.78 (95% UI, 2.16-3.28) to 2.52 (95% UI, 2.40-2.66) in Ireland (eFigure 11 in the Supplement). The ASIR decreased in most countries after a complete ban on asbestos use, and a temporary increase in ASIR occurred shortly after asbestos bans in some countries, followed by a continuous decrease thereafter. For instance, in Australia, which enacted a complete ban in 2002, the ASIR increased from 2.31 (95% UI, 2.18-2.45) in 2002 and peaked at 2.43 (95% UI, 2.27-2.62) in 2008; in Belgium, which enacted a complete ban in 1998, the ASIR increased from 1.29 (95% UI, 1.16-1.47) in 1998 and peaked at 1.47 (95% UI, 1.34-1.65) in 2009. In both countries, the ASIR continuously decreased thereafter.

### Regional Burden of Mesothelioma

**Figure 3.**
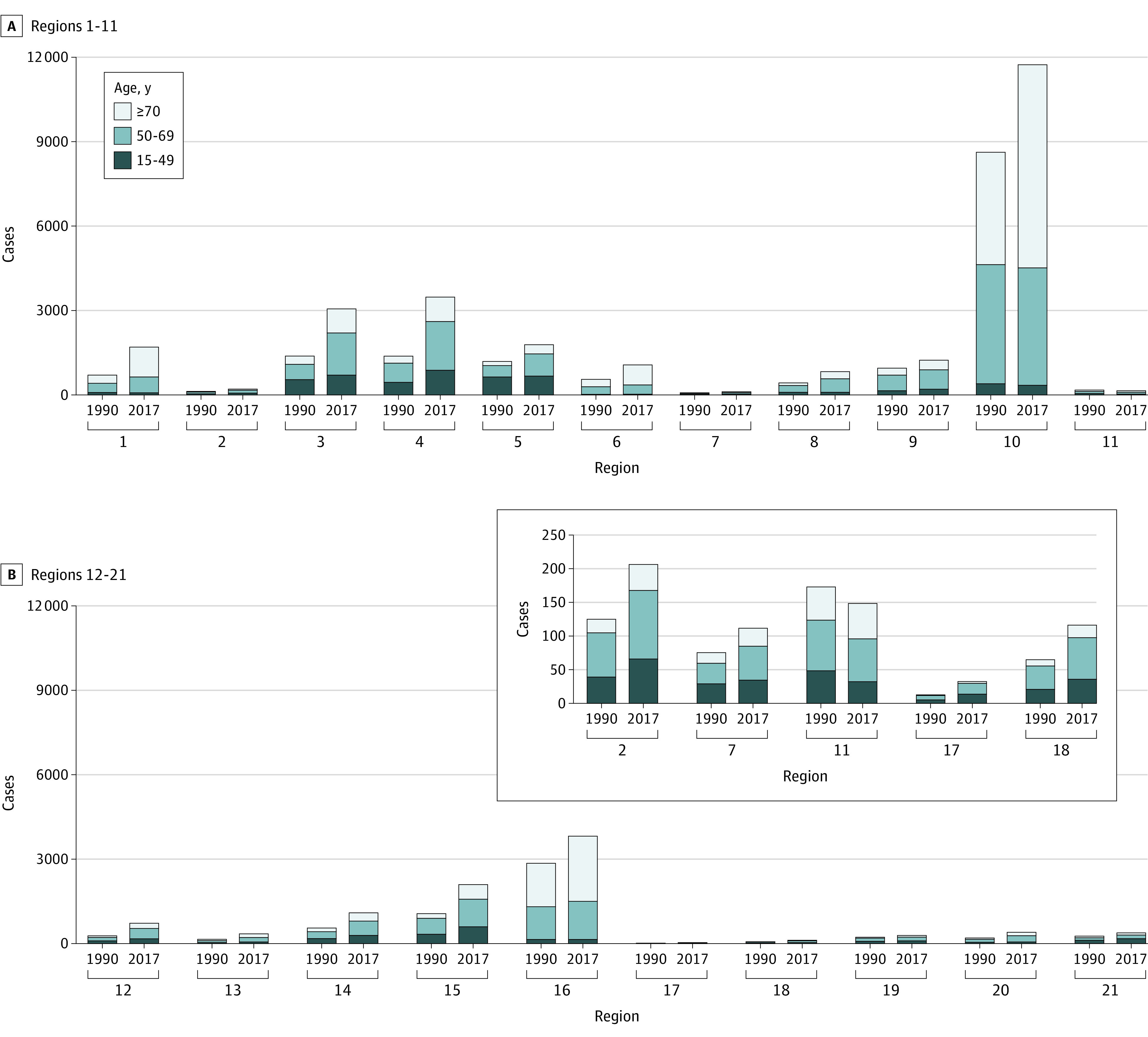
Comparison of Incident Mesothelioma Cases by Age and Region in 1990 and 2017 A, Region 1 indicates high-income Asia Pacific; region 2, central Asia; region 3, East Asia; region 4, South Asia; region 5, Southeast Asia; region 6, Australasia; region 7, Caribbean; region 8, central Europe; region 9, eastern Europe; region 10, western Europe; region 11, Andean Latin America. B, Region 12 indicates central Latin America; region 13, southern Latin America; region 14, tropical Latin America; region 15, North Africa and the Middle East; region 16, high-income North America; region 17, Oceania; region 18, central sub-Saharan Africa; region 19, eastern sub-Saharan Africa; region 20, southern sub-Saharan Africa; and region 21, western sub-Saharan Africa. Data from specific regions (central Asia, Caribbean, Andean Latin America, Oceania, and central sub-Saharan Africa) can be viewed in the top right of panel B.

An increasing number of incident cases was observed in most regions, with the exception of Andean Latin America (decrease from 173 cases [95% UI, 141-216 cases] in 1990 to 148 cases [95% UI, 132-166 cases] in 2017) (Figure 3), and similar temporal trends in deaths and DALYs were also found. The largest absolute increase in both incident cases (from 8620 [95% UI, 7015-9833] in 1990 to 11 729 [95% UI, 11 138-12 369] in 2017) and deaths (from 7147 [95% UI, 5857-8136] in 1990 to 10 303 [95% UI, 9862-10 771] in 2017) occurred in western Europe, although all cases and deaths were among those older than 70 years. In contrast, the decrease in the number of cases in Andean Latin America was mainly observed among patients younger than 50 years (eFigure 12 and eFigure 13 in the Supplement). Distinct age compositions for incident cases and deaths were found in different regions (eg, in 2017, only 8.2% of incident cases occurred among those aged >70 years in Oceania, but >65.0% of incident cases occurred among those aged >70 years in Australasia) (eFigure 4 and eFigure 14 in the Supplement).

Distinctive temporal trends in the ASRs of incident cases, deaths, and DALYs were also observed in various regions. For instance, a significant decrease in ASIR occurred in southern sub-Saharan Africa after 2000 (from 1.11 [95% UI, 1.05-1.16] in 2000 to 0.71 [95% UI, 0.65-0.80] in 2017), whereas before 2000, there was a rapid increase (from 0.67 [95% UI, 0.56-0.80] in 1990). In contrast, the ASIR in Australasia increased (from 2.03 [95% UI, 1.91-2.16] in 1999 to 2.30 [95% UI, 2.16-2.47] in 2008) after a sustained decrease that had occurred before 2000 (from 2.26 [95% UI, 1.69-2.65] in 1990), and the ASIR again decreased in 2008 and thereafter (from 2.30 in 2008 to 2.13 [95% UI, 1.84-2.47] in 2017) (eFigure 2, eFigure 3, and eFigure 15 in the Supplement). Decreasing trends in incidence, mortality, and DALY rates among female individuals were observed in all SDI regions from 1990 to 2017, but increasing trends were found among male individuals in most regions, with the exception of regions with high SDI levels (eFigures 16-18 in the Supplement). A bimodal distribution of ASIR and ASDR was observed in regions with middle to high SDI levels (eg, ASIR peaked at 0.36 [95% UI, 0.20-0.42] in 1999 and 0.33 [95% UI, 0.32-0.34] in 2010) and middle SDI levels, and the rates revealed an increasing trend after a continuous decrease in regions with low SDI levels between 1990 (ASIR, 0.25 [95% UI, 0.15-0.43]) and 2005 (ASIR, 0.21 [95% UI, 0.14-0.33]) (eFigure 19 and eFigure 20 in the Supplement).

### National Burden of Mesothelioma

**Figure 4.**
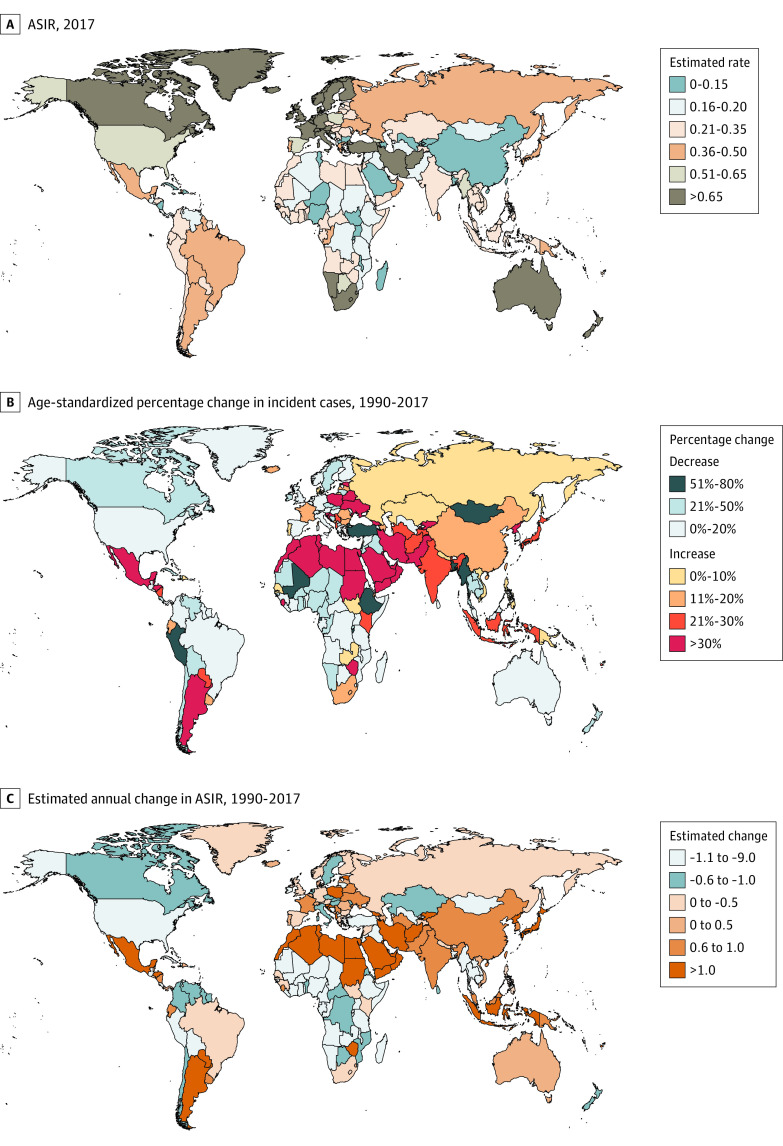
Epidemiological Patterns of Mesothelioma Incidence in 195 Countries and Territories Countries with a large number of cases or a large change in the number of cases were annotated. ASIR indicates age-standardized incidence rates.

A total of 9.6% of global incident cases were recorded in the UK in 2017 (3309 cases [95% UI, 3137-3495 cases]), followed by the US (3172 cases [95% UI, 3044-3309 cases]), where most deaths also occurred (3147 deaths [95% UI, 3024-3280 deaths]) (eTable 4 and eTable 5 in the Supplement). The highest ASIRs in 2017 occurred in the UK, followed by Australia and Andorra (Figure 4; eTable 4 in the Supplement). Notably, the largest ASDRs and ASRs of DALYs also occurred in the UK (ASDR, 2.13 [95% UI, 2.06-2.21]), Andorra (ASDR, 1.95 [95% UI, 1.43-2.42]), and Australia (ASDR, 1.89 [95% UI, 1.67-2.11]) in 2017 (eTable 6 and eTable 7 in the Supplement). The lowest ASIRs, ASDRs, and ASR of DALYs were found in Macedonia, whereas the most substantial decrease in these values was observed in Peru (eFigure 21 and eFigure 22 in the Supplement). The lowest ASIRs and ASDRs among male patients with mesothelioma were recorded in Nigeria in 2017 (ASIR, 0.06 [95% UI, 0.04-0.09]; ASDR, 0.05, [95% UI, 0.03-0.08]) (eFigure 23 in the Supplement). Among female patients with mesothelioma, the highest ASIR (1.10 [95% UI, 0.31-2.58]) and the second-highest ASDR (0.70 [95% UI, 0.21-1.63]) were found in Afghanistan, whereas the lowest ASIR (0.05 [95% UI, 0.04-0.05]) and ASDR (0.03 [95% UI, 0.03-0.04]) were both observed in Dominica (eFigure 24 in the Supplement).

The most distinct increases in both ASIR and ASDR occurred in Georgia, with EAPCs of 7.33 and 7.58, respectively (eFigure 21 and eFigure 22 in the Supplement). In the UK, which had the greatest mesothelioma burden, the ASIR continuously decreased after a complete ban on asbestos was enacted, although incident cases continued to increase for almost 2 decades. In Germany, France, and Italy, the increase in mesothelioma cases generally stabilized and decreased after 20 years of a complete asbestos ban. However, in some countries that imposed an asbestos ban for a short time, such as Brazil and Australia, mesothelioma cases continued to increase (Figure 2; eFigure 11 in the Supplement).

## Discussion

This cross-sectional study is the first, to our knowledge, to use mesothelioma data from the GBD 2017 to find that global mesothelioma cases continuously increased from 1990 to 2017, with more than 50% of cases recorded in regions with high SDI levels. We also found an increasing temporal trend in incidence and mortality in regions with low SDI levels over recent years, especially among female individuals. The proportion of patients with mesothelioma who were older than 70 years continued to increase, but the proportion of patients with mesothelioma who were younger than 50 years decreased over time. Notably, we found that mesothelioma cases began to decrease after 2 decades of a complete ban on asbestos use. Temporal trends in incident cases varied across geographic regions, with the largest absolute increase occurring in western Europe.

The ASR data derived from the GHDx, which could reflect the success of current policies and disease prevention strategies, were considered to be an objective index for comparing the temporal trends in mesothelioma incidence and mortality among different age groups.^21^ Higher ASIRs and ASDRs were observed in regions with higher SDI levels, and EAPCs were positively correlated with SDI levels in most countries. These phenomena suggest that the incidence and mortality of mesothelioma increased as the economy developed along with increases in industrial manufacturing and consequent exposure to asbestos.^13^ However, EAPCs decreased as SDI levels increased to higher than 0.8, suggesting that SDI levels may be associated with the development of fewer cases and lower disease burden in non–resource-limited countries.^22^ The association between EAPC and SDI level may reflect temporal trends in asbestos use as economies developed, suggesting that trends in these countries followed the environmental Kuznets curve, in which the use of asbestos peaked at a certain level of income, then began to decrease.^23^

To prevent the disease burden from shifting toward industrializing countries, then increasing again, the administrations of countries that continue to use large amounts of asbestos and are at risk of experiencing increases in mesothelioma cases and deaths can be made aware of the present findings.^24,25,26^ Given the long latent period of mesothelioma, resource-limited countries are in greater need of more stringent controls on asbestos use, in which a quicker elimination of asbestos use could be expected.^23^ If governments and medical institutions aim to decrease their burden of mesothelioma, they can pursue such strategies.

Mesothelioma is generally considered to be a disease occurring in older patients, primarily because it has a long latent period after initial occupational exposure.^27^ Our results revealed that more than 80% of patients with mesothelioma were 50 years or older and that the proportion of patients who were younger continued to decrease globally over the study period. All new cases identified in western Europe from 1990 to 2017 were among individuals older than 70 years, which is likely because of asbestos exposure during their early occupations.^9^

Given that high occupational exposure is correlated with younger age at diagnosis, the higher number of patients younger than 70 years in regions with low SDI levels is of substantial concern and deserves attention.^28^ In addition, among those younger than 45 years, female individuals had a higher incidence than male individuals, although more incident cases were recorded in male individuals globally, and these cases had a higher death rate. These phenomena may be attributable to latent etiologic, social, or psychological factors, such as different types of risk factors, sex hormone levels, and/or willingness to undergo physical examination. However, the continuous decrease in ASRs among female individuals may reflect socioeconomic improvement and increased protection of women’s rights. To improve the understanding of mesothelioma, further study in this area is warranted.^29^

Peak incidence and mortality in non–resource-limited countries were expected to occur before 2030.^30^ In 1995, Peto et al^9^ estimated that the death rate for mesothelioma may continue to increase for 25 years. The findings of the current study revealed that the global incident cases and deaths associated with mesothelioma continuously increased over the study period, whereas the temporal trends differed between countries. However, after standardization for age, the global incidence and death rates both decreased over the study period, indicating that the increase in incident cases and deaths might be partly owing to the aging of the population and thus varied among countries and regions.^30,31,32^

The highest ASIR occurred in the UK, where the highest ASDR and ASR of DALYs were also observed. This finding highlights the substantial societal burden and disease management challenge that mesothelioma continues to pose. Notably, the most distinct increases in both ASIR and ASDR occurred in Georgia, raising a potential area of concern for the government of that country. In contrast, a substantial decrease in ASIR and ASDR occurred in Peru, suggesting successful prevention and health care policies. Thus, the policies of Peru warrant further study and could be used as a reference. A previous study reported that the incidence of mesothelioma remained high even after the complete banning of asbestos for more than 15 years.^33^ We found that mesothelioma cases began to decrease after a complete ban on asbestos use for more than 2 to 3 decades, suggesting that implementing a complete and immediate ban on asbestos use may be warranted in all countries rather than non–resource-limited countries only.

### Strengths and Limitations

This study has several strengths. The study is the first, to our knowledge, to use data from the GBD 2017 to evaluate worldwide epidemiological patterns of mesothelioma to help policy makers form a targeted management strategy from a global perspective. The current study explored the age and sex composition of mesothelioma cases and deaths as well as the correlation between socioeconomic level and the disease burden of mesothelioma. In addition, the study is the first, to our knowledge, to describe the temporal trends of mesothelioma incidence after a complete asbestos ban using data from dozens of countries.

This study also has limitations. First, the GBD 2017 data were collected from various databases and institutions with inconsistent quality; this inconsistency increases the probability of bias and may have produced heterogeneity in the data. Second, the data could not be explored further for information regarding histologic characteristics, risk factors, and treatment; such information was not provided in the GHDx. Therefore, we could not ascertain a detailed etiologic understanding of global pattern changes. Third, the linear model is not accurate when incidence is close to 0 and could provide negative estimates of incidence. A Poisson mixed model with a space-time Gaussian process can be used in future analyses to allow capture of the correlation between spatial and temporal incidence, which would also allow us to obtain more robust estimates for the uncertainty of the association between incidence and the different risk factors considered.

## Conclusions

In this cross-sectional study, incident cases of mesothelioma and deaths associated with mesothelioma continued to increase globally throughout the study period, especially in non–resource-limited regions with low SDI levels. Banning asbestos was associated with reductions in the increase of mesothelioma incidence and mortality; however, the association between mesothelioma and previous exposure to asbestos remains a concern. Governments and global medical institutions may consider implementing stricter asbestos controls immediately and developing targeted management strategies accordingly.

## Notes

Supplement. **eMethods.** GBD Overview, ASR Estimation, DALY Estimation, Joinpoint Regression Analysis, SDI Estimation, Definition and Estimation of UI, and Inclusion Criteria of Countries With Complete Asbestos Bans **eTable 1.** Symbols and Asbestos Banning Times for 47 Countries Included in Study **eTable 2.** Deaths and Age-Standardized Death Rates for Mesothelioma Among Regions in 1990 and 2017 and Temporal Trends From 1990 to 2017 **eTable 3.** DALYs and Age-Standardized DALY Rates for Mesothelioma Among Regions in 1990 and 2017 and Temporal Trends From 1990 to 2017 **eTable 4.** Countries With the Highest and Lowest ASRs or Temporal Trends Using Different Measures **eTable 5.** Incident Cases of Mesothelioma Among Countries and Territories in 1990 and 2017 and Temporal Trends From 1990 to 2017 **eTable 6.** Deaths and Age-Standardized Death Rates for Mesothelioma Among Countries and Territories in 1990 and 2017 and Temporal Trends From 1990 to 2017 **eTable 7.** DALYs and Age-Standardized DALY Rates for Mesothelioma Among Countries and Territories in 1990 and 2017 and Temporal Trends From 1990 to 2017 **eFigure 1.** Mesothelioma Cases With Age Composition by SDI Region From 1990 to 2017 **eFigure 2.** Age-Standardized Incidence Rates for Mesothelioma per 100 000 People in 21 Geographic Regions by SDI in 2017 **eFigure 3.** Age-Standardized Death Rates for Mesothelioma per 100 000 People in 21 Geographic Regions by SDI in 2017 **eFigure 4.** Proportion of Age Subgroups of Incident Cases by Region and Year in 1990 and 2017 **eFigure 5.** Proportion of Age Subgroups of Incident Cases by Year Among Female and Male Patients **eFigure 6.** Mesothelioma Cases With Age Composition Among Female Patients by SDI Region From 1990 to 2017 **eFigure 7.** Mesothelioma Cases With Age Composition Among Men by SDI Region From 1990 to 2017 **eFigure 8.** Comparison of Incidence Rates With Age Composition Between Female and Male Patients **eFigure 9.** Comparison of Death Rates With Age Composition Between Female and Male Patients **eFigure 10.** Comparison of DALY Rates With Age Composition Between Female and Male Patients **eFigure 11.** Temporal Trends of Age-Standardized Incidence Rates (ASIRs) for Mesothelioma per 100 000 People in 47 Countries With Complete Asbestos Bans **eFigure 12.** Comparison Between Deaths in 1990 vs 2017 With Age Composition at Regional Level **eFigure 13.** Comparison Between DALYs in 1990 vs 2017 With Age Composition at Regional Level **eFigure 14.** Proportion of Deaths in Age Subgroups by Region and Year **eFigure 15.** Age-Standardized DALY Rates for Mesothelioma per 100 000 People in 21 Geographic Regions by SDI in 2017 **eFigure 16.** Temporal Trends of Age-Standardized Incidence, Death, and DALY Rates for Mesothelioma by SDI Level Among Female and Male Patients **eFigure 17.** Joinpoint Regression Analysis of Age-Standardized Incidence Rates (ASIRs) Among Female Patients in SDI Regions From 1990 to 2017 **eFigure 18.** Joinpoint Regression Analysis of Age-Standardized Incidence Rates (ASIRs) Among Male Patients in SDI Regions From 1990 to 2017 **eFigure 19.** Joinpoint Regression Analysis of Age-Standardized Incidence Rates (ASIRs) in SDI Regions From 1990 to 2017 **eFigure 20.** Joinpoint Regression Analysis of Age-Standardized Death Rates (ASDRs) in SDI Regions From 1990 to 2017 **eFigure 21.** Epidemiological Patterns of Mesothelioma Deaths in 195 Countries and Territories **eFigure 22.** Epidemiological Patterns of Mesothelioma DALYs in 195 Countries and Territories **eFigure 23.** Epidemiological Patterns Among Male Patients in 195 Countries and Territories **eFigure 24.** Epidemiological Patterns Among Female Patients in 195 Countries and Territories
eReferences
