# Supplementary material for: Assessment of Global Trends in the Diagnosis of Mesothelioma From 1990 to 2017
Source: JAMA Netw Open. 2021 Aug 11;4(8):e2120360. doi: 10.1001/jamanetworkopen.2021.20360 (PMC8358735; doi:10.1001/jamanetworkopen.2021.20360)
Supplement: Supplement. — eMethods. GBD Overview, ASR Estimation, DALY Estimation, Joinpoint Regression Analysis, SDI Estimation, Definition and Estimation of UI, and Inclusion Criteria of Countries With Complete Asbestos Bans eTable 1. Symbols and Asbestos Banning Times for 47 Countries Included in Study eTable 2. Deaths and Age-Standardized Death Rates for Mesothelioma Among Regions in 1990 and 2017 and Temporal Trends From 1990 to 2017 eTable 3. DALYs and Age-Standardized DALY Rates for Mesothelioma Among Regions in 1990 and 2017 and Temporal Trends From 1990 to 2017 eTable 4. Countries With the Highest and Lowest ASRs or Temporal Trends Using Different Measures eTable 5. Incident Cases of Mesothelioma Among Countries and Territories in 1990 and 2017 and Temporal Trends From 1990 to 2017 eTable 6. Deaths and Age-Standardized Death Rates for Mesothelioma Among Countries and Territories in 1990 and 2017 and Temporal Trends From 1990 to 2017 eTable 7. DALYs and Age-Standardized DALY Rates for Mesothelioma Among Countries and Territories in 1990 and 2017 and Temporal Trends From 1990 to 2017 eFigure 1. Mesothelioma Cases With Age Composition by SDI Region From 1990 to 2017 eFigure 2. Age-Standardized Incidence Rates for Mesothelioma per 100 000 People in 21 Geographic Regions by SDI in 2017 eFigure 3. Age-Standardized Death Rates for Mesothelioma per 100 000 People in 21 Geographic Regions by SDI in 2017 eFigure 4. Proportion of Age Subgroups of Incident Cases by Region and Year in 1990 and 2017 eFigure 5. Proportion of Age Subgroups of Incident Cases by Year Among Female and Male Patients eFigure 6. Mesothelioma Cases With Age Composition Among Female Patients by SDI Region From 1990 to 2017 eFigure 7. Mesothelioma Cases With Age Composition Among Men by SDI Region From 1990 to 2017 eFigure 8. Comparison of Incidence Rates With Age Composition Between Female and Male Patients eFigure 9. Comparison of Death Rates With Age Composition Between Female and Male Patients eFigure 10. Comparison of [file jamanetwopen-e2120360-s001.pdf]

Supplementary Online Content

Zhai Z, Ruan J, Zheng Y, et al. Assessment of global trends in the diagnosis of mesothelioma from 1990 to 2017. *JAMA Netw Open*. 2021;4(8):e2120360. doi:10.1001/jamanetworkopen.2021.20360

eMethods. GBD Overview, ASR Estimation, DALY Estimation, Joinpoint Regression Analysis, SDI Estimation, Definition and Estimation of UI, and Inclusion Criteria of Countries With Complete Asbestos Bans

eTable 1. Symbols and Asbestos Banning Times for 47 Countries Included in Study

eTable 2. Deaths and Age-Standardized Death Rates for Mesothelioma Among Regions in 1990 and 2017 and Temporal Trends From 1990 to 2017

eTable 3. DALYs and Age-Standardized DALY Rates for Mesothelioma Among Regions in 1990 and 2017 and Temporal Trends From 1990 to 2017

eTable 4. Countries With the Highest and Lowest ASRs or Temporal Trends Using Different Measures

eTable 5. Incident Cases of Mesothelioma Among Countries and Territories in 1990 and 2017 and Temporal Trends From 1990 to 2017

eTable 6. Deaths and Age-Standardized Death Rates for Mesothelioma Among Countries and Territories in 1990 and 2017 and Temporal Trends From 1990 to 2017

eTable 7. DALYs and Age-Standardized DALY Rates for Mesothelioma Among Countries and Territories in 1990 and 2017 and Temporal Trends From 1990 to 2017

eFigure 1. Mesothelioma Cases With Age Composition by SDI Region From 1990 to 2017

eFigure 2. Age-Standardized Incidence Rates for Mesothelioma per 100 000 People in 21 Geographic Regions by SDI in 2017

eFigure 3. Age-Standardized Death Rates for Mesothelioma per 100 000 People in 21 Geographic Regions by SDI in 2017

eFigure 4. Proportion of Age Subgroups of Incident Cases by Region and Year in 1990 and 2017

eFigure 5. Proportion of Age Subgroups of Incident Cases by Year Among Female and Male Patients

eFigure 6. Mesothelioma Cases With Age Composition Among Female Patients by SDI Region From 1990 to 2017

eFigure 7. Mesothelioma Cases With Age Composition Among Men by SDI Region From 1990 to 2017

eFigure 8. Comparison of Incidence Rates With Age Composition Between Female and Male Patients

© 2021 Zhai Z et al. *JAMA Network Open*.

eFigure 9. Comparison of Death Rates With Age Composition Between Female and Male Patients

eFigure 10. Comparison of DALY Rates With Age Composition Between Female and Male Patients

eFigure 11. Temporal Trends of Age-Standardized Incidence Rates (ASIRs) for Mesothelioma per 100 000 People in 47 Countries With Complete Asbestos Bans

eFigure 12. Comparison Between Deaths in 1990 vs 2017 With Age Composition at Regional Level

eFigure 13. Comparison Between DALYs in 1990 vs 2017 With Age Composition at Regional Level

eFigure 14. Proportion of Deaths in Age Subgroups by Region and Year

eFigure 15. Age-Standardized DALY Rates for Mesothelioma per 100 000 People in 21 Geographic Regions by SDI in 2017

eFigure 16. Temporal Trends of Age-Standardized Incidence, Death, and DALY Rates for Mesothelioma by SDI Level Among Female and Male Patients

eFigure 17. Joinpoint Regression Analysis of Age-Standardized Incidence Rates (ASIRs) Among Female Patients in SDI Regions From 1990 to 2017

eFigure 18. Joinpoint Regression Analysis of Age-Standardized Incidence Rates (ASIRs) Among Male Patients in SDI Regions From 1990 to 2017

eFigure 19. Joinpoint Regression Analysis of Age-Standardized Incidence Rates (ASIRs) in SDI Regions From 1990 to 2017

eFigure 20. Joinpoint Regression Analysis of Age-Standardized Death Rates (ASDRs) in SDI Regions From 1990 to 2017

eFigure 21. Epidemiological Patterns of Mesothelioma Deaths in 195 Countries and Territories

eFigure 22. Epidemiological Patterns of Mesothelioma DALYs in 195 Countries and Territories

eFigure 23. Epidemiological Patterns Among Male Patients in 195 Countries and Territories

eFigure 24. Epidemiological Patterns Among Female Patients in 195 Countries and Territories

eReferences

This supplementary material has been provided by the authors to give readers additional information about their work.

© 2021 Zhai Z et al. *JAMA Network Open*.

eMethods. GBD Overview, ASR Estimation, DALY Estimation, Joinpoint Regression Analysis, SDI Estimation, Definition and Estimation of UI, and Inclusion Criteria of Countries With Complete Asbestos Bans

GBD Overview

The Global Burden of Disease (GBD) is an approach to global descriptive epidemiology. It is a systematic, scientific effort to quantify the comparative magnitude of health loss due to diseases, injuries, and risk factors by age, sex, and geographies for specific points in time. IHME serves as the coordinating center for the GBD and affiliated projects.

GBD produces regular estimates of all-cause mortality, deaths by cause, years of life lost due to premature mortality (YLLs), years lived with disability (YLDs), and disability-adjusted life years (DALYs) for a cause list. The cause list is agreed upon annually by the Scientific Council. The critical milestones for ongoing estimation include regular updates to the GBD estimates, referred to as the "GBD round." For each round, the entire time series back to 1990 is re-estimated using all available data to ensure the most complete and highly comparable set of estimates possible. Previous results will be archived every time new results are released. The GBD provides cutting-edge and timely results through scientific papers, policy reports, web content, and interactive visualizations.

Published in November 2018, GBD 2017 provides for the first time an independent estimation of population, for each of 195 countries and territories and the globe, using a standardized, replicable approach, as well as a comprehensive update on fertility. GBD 2017 incorporates major data additions and improvements, and methodological refinements. Mortality and life expectancy estimates have been extended back to 1950, and new causes have been added to the fatal and non-fatal cause lists, for a total of 359 diseases and injuries

© 2021 Zhai Z et al. *JAMA Network Open*.

(http://www.healthdata.org/gbd/about/protocol).

ASR Estimation

Age-standardized rate was determined to compare the age structure of different groups or the age structure in the same population with changes over time, considered as the objective index to quantify the incidence trends of cancer.

The computational method of the ASR (per 100,000 population) was calculated by the sum of the products of age-specific rates (a_i, where i is the ith age) and the number of population (or weight) (w_i) in the same age group of i in the population which was selected as reference standard population, and then, divide by the sum of the standard population weights, ie (1).

$$\text{ASR} = \frac{\sum_{i=1}^{A} a_i w_i}{\sum_{i=1}^{A} w_i} \times 100,000$$

DALY Estimation

Disability adjusted life-years were also defined as years of healthy life lost, estimated by the sum of the YLLs and the YLDs. YLLs, the years of life lost due to premature mortality, are the multiplication of deaths and a standard life expectancy at each age of death. In each population greater than 5 million, the standard life expectancy was estimated from a life table which includes the lowest observed mortality at each age group (2). YLDs were defined as the years lived with any short-term or long-term health loss weighted for severity by the disability weights, calculated by multiplying prevalence estimate and the disability weight of each mutually exclusive sequela for prostate cancer, such as disability due to incontinence and impotence from prostatectomy (3).

Joinpoint Regression Analysis

© 2021 Zhai Z et al. *JAMA Network Open*.

The joinpoint regression model, which is composed of a few continuous linear phases, is often useful to describe changes in trend data. Joinpoints are the best-fitting point where a statistically significant change occurred, identified with the Joinpoint Regression Program (version 4.7.0.0). The optimum number of joinpoints will be recommended by the program, which ranged from 0 to 5. Annual percentage change (APC) between successive joinpoints were measured to reflect the temporal trends of incidence and mortality rates. The Monte Carlo permutation method was used to test significant difference, and two-sided *P* values less than 0.05 were considered to indicate statistical significance (4).

SDI Estimation

Socio-demographic index is a compound measure of income, average years of schooling and fertility for each GBD location and year which is used as a measure of socio-demographic development.

Developed by GBD researchers and used to help produce these estimates, SDI is a composite indicator of development status strongly correlated with health outcomes. It is the geometric mean of 0 to 1 indices of total fertility rate under the age of 25 (TFU25), mean education for those ages 15 and older (EDU15+), and lag distributed income (LDI) per capita. As a composite, a location with an SDI of 0 would have a theoretical minimum level of development relevant to health, while a location with an SDI of 1 would have a theoretical maximum level (5).

Definition and Estimation of UI

Uncertainty intervals are a range of values that reflects the certainty of an estimate. In GBD, every estimate is calculated 1,000 times, each time sampling from distributions rather

than point estimates for data inputs, data transformations and model choice. The 95th uncertainty interval is determined by the 25th and 975th value of the 1,000 values after ordering them from smallest to largest. Larger uncertainty intervals can result from limited data availability, small studies, and conflicting data, while smaller uncertainty intervals can result from extensive data availability, large studies, and data that are consistent across sources (http://ghdx.healthdata.org/gbd-2017).

Inclusion criteria of the countries with complete asbestos bans

The chronology of asbestos bans and restrictions were extracted from the International Ban Asbestos Secretariat (http://www.ibasecretariat.org/index.htm), to explore the temporal trend of mesothelioma incidence after complete asbestos bans (including chrysotile). There were 67 countries and territories recorded in the list of current asbestos bans. However, compliance with this directive has not been verified in Cyprus, Czech Republic, Greece, Hungary, Lithuania, Malta, Portugal, and Slovakia, which were excluded from this analysis. In addition, Croatia, Djibouti, Gabon, New Zealand, Qatar, Seychelles, Slovenia, Taiwan (China), and Uruguay, were excluded from our list for lack of specific schedule or even enforcement of the complete bans. Gibraltar, Liechtenstein, Monaco, New Caledonia were mot enrolled in the GBD database and incapable of analyzing. Nepal were added into our list, which enforced a ban on the import, sale, distribution and use of all asbestos in 2015 and was not recorded in the current bans list. The 47 countries finally included in our analysis were listed below.

© 2021 Zhai Z et al. *JAMA Network Open*.

eTable 1. Symbols and Asbestos Banning Times for 47 Countries Included in Study

Countries	Symbol	Time for complete bans (year)	Countries	Symbol	Time for complete bans (year)
Algeria	●	2009	Japan	●	2012
Argentina	▲	2001	Jordan	▲	2006
Australia	■	2002	Kuwait	■	1995
Austria	+	1990	Latvia	+	2001
Bahrain	⊠	1996	Luxembourg	⊠	2002
Belgium	❋	1998	Macedonia	❋	2015
Brazil	●	2017	Mauritius	●	2004
Brunei	▲	1994	Mozambique	▲	2010
Bulgaria	■	2005	Nepal	■	2015
Canada	+	2018	Netherlands	+	1991
Chile	⊠	2001	Norway	⊠	1984
Colombia	❋	2019	Oman	❋	2008
Denmark	●	1985	Poland	●	1997
Egypt	▲	2005	Romania	▲	2008
Estonia	■	2001	Saudi Arabia	■	1998
Finland	+	1993	Serbia	+	2011
France	⊠	1996	South Africa	⊠	2008
Germany	❋	1993	South Korea	❋	2009
Honduras	●	2004	Spain	●	2002
Iceland	▲	1983	Sweden	▲	1986
Iraq	■	2016	Switzerland	■	1989
Ireland	+	2000	Turkey	+	2010
Israel	⊠	1984	United Kingdom	⊠	1999
Italy	❋	1992			

© 2021 Zhai Z et al. *JAMA Network Open*.

eTable 2. Deaths and Age-Standardized Death Rates for Mesothelioma Among Regions in 1990 and 2017 and Temporal Trends From 1990 to 2017

Characteristics	1990		2017		1990-2017
	Death cases	ASDR per 100,000	Death cases	ASDR per 100,000	EAPC
	No. $\times 10^2$ (95% UI)	No. (95% UI)	No. $\times 10^2$ (95% UI)	No. (95% UI)	No. (95% CI)
**Global**	174.06 (144.95- 206.6)	0.44 (0.37- 0.52)	299.09 (291.34- 306.13)	0.38 (0.37- 0.39)	-0.44 (-0.52- -0.37)
**Socio-demographic index**					
High SDI	112.26 (94.46- 125.92)	0.85 (0.72- 0.95)	171.93 (165.5- 178.76)	0.75 (0.72- 0.78)	-0.39 (-0.44- -0.34)
High-middle SDI	24.11 (20.52- 29.77)	0.25 (0.21- 0.31)	44.72 (42.2- 46.53)	0.25 (0.24- 0.26)	0.1 (-0.11- 0.3)
Middle SDI	18.64 (16.25- 22.7)	0.19 (0.16- 0.22)	42.94 (40.82- 44.85)	0.19 (0.19- 0.2)	0.01 (-0.14- 0.15)
Low-middle SDI	11.96 (8.35- 18.31)	0.2 (0.14- 0.3)	25.74 (22.74- 30.11)	0.22 (0.19- 0.25)	0.29 (0.26- 0.32)
Low SDI	6.77 (3.99- 11.92)	0.19 (0.12- 0.33)	13.1 (9.96- 18.66)	0.18 (0.14- 0.26)	-0.29 (-0.48- -0.09)
**Regions**					
Andean Latin America	1.35 (1.12- 1.66)	0.64 (0.53- 0.79)	1.21 (1.09- 1.33)	0.22 (0.2- 0.25)	-6 (-6.99- -4.99)
Australasia	4.78 (3.62- 5.5)	1.95 (1.48- 2.23)	9.01 (8.06- 10)	1.79 (1.59- 1.98)	-0.18 (-0.32- -0.04)
Caribbean	0.55 (0.43- 0.72)	0.2 (0.16- 0.26)	0.86 (0.76- 1)	0.17 (0.15- 0.2)	-0.9 (-1.02- -0.77)
Central Asia	0.82 (0.74- 0.95)	0.16 (0.15- 0.19)	1.43 (1.36- 1.5)	0.19 (0.18- 0.2)	0.3 (-0.09- 0.69)
Central Europe	2.95 (2.67- 3.39)	0.2 (0.18- 0.23)	6.24 (5.88- 6.64)	0.3 (0.29- 0.32)	2.15 (1.99- 2.32)
Central Latin America	2.08 (1.83- 2.38)	0.23 (0.2- 0.26)	5.77 (5.52- 6.02)	0.24 (0.23- 0.26)	0.44 (0.28- 0.6)
Central Sub-Saharan Africa	0.46 (0.28- 0.77)	0.19 (0.11- 0.3)	0.85 (0.64- 1.19)	0.16 (0.12- 0.22)	-0.87 (-1.02- -0.72)
East Asia	12.26 (9.61- 16.07)	0.13 (0.1- 0.17)	29.28 (27.09- 31.02)	0.14 (0.13- 0.15)	1.08 (0.64- 1.51)
Eastern Europe	6.51 (5.7- 7.86)	0.23 (0.2- 0.28)	8.84 (8.47- 9.25)	0.26 (0.25- 0.28)	0.11 (-0.28- 0.5)
Eastern Sub-Saharan Africa	1.59 (0.86- 2.88)	0.19 (0.1- 0.33)	2.15 (1.42- 2.83)	0.13 (0.09- 0.17)	-1.87 (-2.11- -1.63)
High-income Asia Pacific	6.36 (5.62- 6.85)	0.32 (0.28- 0.34)	17.02 (16.14- 18.31)	0.37 (0.35- 0.4)	1.14 (0.91- 1.38)

© 2021 Zhai Z et al. *JAMA Network Open*.

High-income North America	27.74 (24.57- 30.14)	0.74 (0.66- 0.81)		37.88 (36.39- 39.45)	0.6 (0.58- 0.63)		-1 (-1.11- -0.88)
North Africa and Middle East	7.7 (4.67- 11.73)	0.42 (0.26- 0.63)		16.69 (14.8- 18.48)	0.4 (0.36- 0.44)		0.07 (-0.25- 0.39)
Oceania	0.09 (0.06- 0.15)	0.26 (0.18- 0.41)		0.23 (0.17- 0.35)	0.31 (0.24- 0.46)		1.04 (0.9- 1.17)
South Asia	10.34 (6.72- 17.64)	0.17 (0.11- 0.28)		27.56 (23.37- 34.82)	0.21 (0.17- 0.26)		0.73 (0.57- 0.89)
Southeast Asia	8.11 (6- 11.69)	0.27 (0.21- 0.39)		13.38 (12.44- 14.48)	0.22 (0.21- 0.24)		-1.13 (-1.32- -0.94)
Southern Latin America	1.28 (1.16- 1.42)	0.27 (0.25- 0.3)		3 (2.74- 3.3)	0.37 (0.34- 0.41)		1.78 (1.47- 2.08)
Southern Sub-Saharan Africa	1.61 (1.36- 1.9)	0.57 (0.48- 0.68)		3.37 (3.06- 3.76)	0.63 (0.57- 0.7)		0.19 (-0.52- 0.9)
Tropical Latin America	4.13 (3.73- 5.06)	0.44 (0.4- 0.55)		8.64 (8.08- 9.05)	0.37 (0.35- 0.39)		-0.3 (-0.54- -0.05)
Western Europe	71.47 (58.57- 81.36)	1.19 (0.97- 1.35)		103.03 (98.62- 107.71)	1.11 (1.06- 1.16)		-0.14 (-0.24- -0.04)
Western Sub-Saharan Africa	1.86 (1.26- 2.42)	0.2 (0.13- 0.25)		2.66 (2.2- 3.17)	0.14 (0.11- 0.16)		-1.87 (-2.11- -1.64)

ASDR: age standardized death rate; CI: confidence interval; EAPC: estimated annual percentage change; SDI: Socio-demographic index; UI: uncertainty interval.

© 2021 Zhai Z et al. *JAMA Network Open*.

eTable 3. DALYs and Age-Standardized DALY Rates for Mesothelioma Among Regions in 1990 and 2017 and Temporal Trends From 1990 to 2017

Characteristics	1990		2017		1990-2017
	DALY cases	ASR per 100,000	DALY cases	ASR per 100,000	EAPC
	No. $\times 10^3$ (95% UI)	No. (95% UI)	No. $\times 10^3$ (95% UI)	No. (95% UI)	No. (95% CI)
**Global**	431.94 (352.21- 527.02)	10.03 (8.25- 12.14)	670.69 (648.2- 693.05)	8.31 (8.03- 8.58)	-0.71 (-0.76- -0.65)
**Socio-demographic index**					
High SDI	235.84 (197.32- 264.82)	18.49 (15.5- 20.75)	311.21 (299.63- 324.45)	14.89 (14.32- 15.55)	-0.78 (-0.81- -0.76)
High-middle SDI	71.63 (60.1- 89.54)	7 (5.89- 8.72)	118.21 (111.27- 123.44)	6.53 (6.14- 6.82)	-0.25 (-0.44- -0.05)
Middle SDI	61.17 (52.08- 75.44)	5.21 (4.52- 6.38)	122.5 (116- 127.92)	5.24 (4.97- 5.47)	-0.19 (-0.35- -0.03)
Low-middle SDI	40.24 (26.27- 63.62)	5.66 (3.86- 8.79)	76.77 (66.76- 90.31)	5.68 (4.98- 6.66)	-0.06 (-0.11- -0.02)
Low SDI	22.1 (12.63- 39.74)	5.37 (3.15- 9.53)	40.27 (30.34- 58.52)	4.84 (3.69- 6.95)	-0.48 (-0.67- -0.29)
**Regions**					
Andean Latin America	3.78 (3.11- 4.7)	15.8 (13.05- 19.64)	3.04 (2.72- 3.36)	5.43 (4.86- 6)	-5.93 (-6.86- -5)
Australasia	10.08 (7.58- 11.58)	41.79 (31.44- 47.89)	15.63 (13.92- 17.36)	33.06 (29.54- 36.69)	-0.83 (-0.95- -0.71)
Caribbean	1.78 (1.31- 2.39)	6.06 (4.53- 8.11)	2.52 (2.18- 3.06)	4.98 (4.29- 6.05)	-0.94 (-1.07- -0.8)
Central Asia	2.63 (2.39- 3.03)	4.82 (4.37- 5.56)	4.38 (4.16- 4.58)	5.23 (4.97- 5.47)	-0.07 (-0.49- 0.34)
Central Europe	8.43 (7.61- 9.7)	5.67 (5.1- 6.52)	15.22 (14.34- 16.27)	8.06 (7.6- 8.64)	1.74 (1.59- 1.89)
Central Latin America	6.53 (5.68- 7.51)	6.31 (5.54- 7.23)	16.03 (15.3- 16.79)	6.54 (6.24- 6.85)	0.26 (0.11- 0.41)
Central Sub-Saharan Africa	1.51 (0.9- 2.56)	5.13 (3.12- 8.52)	2.7 (2.01- 3.87)	4.2 (3.17- 5.93)	-1.09 (-1.24- -0.93)
East Asia	40.53 (30.26- 54.09)	3.68 (2.82- 4.88)	81.36 (75.11- 86.41)	3.93 (3.63- 4.17)	0.68 (0.23- 1.13)
Eastern Europe	17.51 (15.28- 21.48)	6.2 (5.41- 7.61)	22.67 (21.66- 23.83)	7.22 (6.89- 7.61)	0.03 (-0.44- 0.5)
Eastern Sub-Saharan Africa	5.24 (2.84- 9.78)	5.36 (2.91- 9.81)	6.71 (4.55- 9.27)	3.41 (2.27- 4.53)	-2.27 (-2.54- -2)
High-income Asia Pacific	14.57 (12.96- 15.62)	6.95 (6.17- 7.45)	29.5 (27.87- 32.02)	7.55 (7.13- 8.25)	0.82 (0.57- 1.08)

© 2021 Zhai Z et al. *JAMA Network Open*.

High-income North America	55.06 (48.6- 59.96)	15.51 (13.71- 16.9)		67.85 (65.33- 70.4)	11.4 (10.99- 11.82)		-1.47 (-1.62- -1.31)
North Africa and Middle East	24.08 (14.22- 37.04)	11.6 (6.96- 17.73)		47.2 (40.95- 53.75)	9.84 (8.64- 11.04)		-0.47 (-0.76- -0.18)
Oceania	0.31 (0.2- 0.53)	7.7 (5.15- 12.73)		0.82 (0.58- 1.27)	9.17 (6.72- 13.93)		0.98 (0.83- 1.12)
South Asia	32.58 (20.48- 57.06)	4.48 (2.89- 7.7)		78.06 (65.59- 100.78)	5.23 (4.43- 6.68)		0.52 (0.36- 0.68)
Southeast Asia	30.34 (21.31- 45.61)	8.62 (6.28- 12.61)		42.75 (39.06- 46.77)	6.35 (5.82- 6.93)		-1.52 (-1.76- -1.28)
Southern Latin America	3.45 (3.08- 3.84)	7.23 (6.47- 8.06)		7.19 (6.53- 7.96)	9.21 (8.36- 10.2)		1.48 (1.2- 1.77)
Southern Sub-Saharan Africa	4.54 (3.86- 5.24)	14.58 (12.35- 17.07)		8.57 (7.77- 9.64)	14.59 (13.22- 16.32)		-0.27 (-1.05- 0.52)
Tropical Latin America	12.53 (11.26- 15.56)	11.52 (10.38- 14.13)		23.94 (22.41- 25.2)	9.85 (9.22- 10.36)		-0.26 (-0.46- -0.07)
Western Europe	150.4 (122.62- 171.22)	26.33 (21.54- 29.97)		185.75 (177.51- 195.37)	22.37 (21.33- 23.58)		-0.57 (-0.62- -0.53)
Western Sub-Saharan Africa	6.07 (4.11- 7.98)	5.48 (3.7- 7.13)		8.79 (7.19- 10.73)	3.64 (3.02- 4.37)		-2.09 (-2.34- -1.84)

ASR: age standardized rate; CI: confidence interval; DALY: disability adjusted life-year; EAPC: estimated annual percentage change; SDI: Socio-demographic index; UI: uncertainty interval.

© 2021 Zhai Z et al. *JAMA Network Open*.

eTable 4. Countries With the Highest and Lowest ASRs or Temporal Trends Using Different Measures

Measure		ASR (per 100k)				Percent Change (%)				EAPC		
		1st	2nd	3rd		1st	2nd	3rd		1st	2nd	3rd
Incidence	Max	UK (2.52)	Australia (2.30)	Andorra (2.25)		Georgia (343%)	United Arab Emirates (224%)	Libya (205%)		Georgia (7.33)	Croatia (5.39)	Iran (5.21)
	Min	Macedonia (0.076)	Palestine (0.084)	Cape Verde (0.089)		Peru (-77%)	Maldives (-63%)	Mongolia (-62%)		Peru (-8.18)	Mongolia (-5.23)	Rwanda (-4.19)
Death	Max	UK (2.13)	Andorra (1.95)	Australia (1.89)		Iran (490%)	Qatar (425%)	United Arab Emirates (397%)		Georgia (7.58)	Croatia (5.74)	Iran (5.38)
	Min	Macedonia (0.053)	Palestine (0.065)	Cape Verde (0.071)		Peru (-45%)	Bermuda (-30%)	Grenada (-26%)		Peru (-8.20)	Mongolia (-4.98)	Burundi (-3.83)
DALY	Max	UK (40.10)	Andorra (38.83)	Australia (34.82)		Iran (566%)	Georgia (457%)	Saudi Arabia (414%)		Georgia (7.60)	Croatia (5.28)	United Arab Emirates (4.90)
	Min	Macedonia (1.61)	Palestine (1.94)	Cape Verde (1.95)		Bermuda (-36%)	Peru (-32%)	Mali (-22%)		Peru (-8.14)	Mongolia (-4.89)	Maldives (-4.42)
		ASIR (per 100k)				ASDR (per 100k)				EAPC of ASIR		
		1st	2nd	3rd		1st	2nd	3rd		1st	2nd	3rd
Female	Max	Afghanistan (1.10)	Australia (0.85)	UK (0.80)		UK (0.74)	Afghanistan (0.70)	Australia (0.65)		Georgia (5.60)	Argentina (4.02)	Lesotho (3.91)
	Min	Dominica (0.046)	Guam (0.053)	Palestine (0.055)		Dominica (0.031)	Tunisia (0.034)	Guam (0.038)		Mongolia (-8.91)	Maldives (-8.76)	Peru (-8.40)
Male	Max	UK (4.60)	Andorra (4.27)	Australia (3.98)		UK (3.80)	Andorra (3.76)	Australia (3.33)		Georgia (9.28)	Croatia (6.33)	Libya (5.94)
	Min	Nigeria (0.058)	Mali (0.067)	Niger (0.070)		Nigeria (0.052)	Mali (0.059)	Niger (0.060)		Peru (-7.98)	Ghana (-4.06)	Bermuda (-3.37)

ASR: age standardized rate; ASDR: age standardized death rate; ASIR: age standardized incidence rate; DALY: disability adjusted life-year; EAPC: estimated annual percentage change.

© 2021 Zhai Z et al. *JAMA Network Open*.

eTable 5. Incident Cases of Mesothelioma Among Countries and Territories in 1990 and 2017 and Temporal Trends From 1990 to 2017

Location	Incident Cases No. (95% UI)		Age-standardized percent change (%)	EAPC No. (95% CI)
	1990	2017	1990-2017	1990-2017
Afghanistan	49.17 (11.37- 132.98)	134.74 (45.73- 309.66)	20.72 (-22.88- 182.25)	1.09 (0.64- 1.53)
Albania	3.38 (2.16- 5.14)	7.05 (4.21- 12.53)	24.37 (-16.45- 78.63)	1.15 (0.87- 1.42)
Algeria	10.14 (7.15- 13.6)	53.24 (39.86- 76.36)	116.6 (39.82- 209.39)	3.7 (3.2- 4.21)
American Samoa	0.09 (0.07- 0.1)	0.21 (0.18- 0.25)	16.07 (-11.69- 48.09)	1.94 (1.5- 2.39)
Andorra	1.47 (1.09- 1.87)	2.95 (2.17- 3.74)	-9.82 (-37.31- 30.45)	-0.3 (-0.39- -0.22)
Angola	13.6 (6.18- 25.8)	26.19 (17.71- 37.93)	-19.94 (-49.15- 39.26)	-1.46 (-1.61- -1.31)
Antigua and Barbuda	0.09 (0.08- 0.1)	0.11 (0.1- 0.13)	-41.34 (-51.44- -29.95)	-2.37 (-2.69- -2.05)
Argentina	95.75 (86.37- 108.63)	257.98 (224.94- 298.81)	58.68 (28.89- 91.9)	2.77 (2.39- 3.15)
Armenia	21.28 (17.11- 28.33)	27.91 (24.79- 31.47)	-7.49 (-31.11- 18.9)	-0.44 (-0.7- -0.18)
Australia	487.07 (366.51- 578.13)	967.98 (819.09- 1146.4)	-6.02 (-26.41- 32.03)	0.05 (-0.1- 0.21)
Austria	103.94 (77.61- 124.62)	133.31 (119.44- 153.74)	-12.62 (-32.5- 26.32)	-0.04 (-0.29- 0.22)
Azerbaijan	6.92 (5.81- 8.15)	13.97 (11.6- 16.95)	13.55 (-9.51- 43.45)	0.2 (-0.29- 0.68)
Bahrain	0.43 (0.35- 0.52)	3.17 (2.56- 3.9)	58.26 (15.24- 115.65)	3.01 (1.92- 4.12)
Bangladesh	145.97 (83.59- 270.59)	153.43 (111.58- 224.36)	-52.96 (-67.04- -27.14)	-2.99 (-3.1- -2.87)
Barbados	0.73 (0.63- 0.84)	1.02 (0.89- 1.18)	-18.46 (-34.04- 1.87)	-0.99 (-1.2- -0.78)
Belarus	25.97 (20.52- 34.07)	39.38 (33.42- 47.42)	30.52 (0.99- 64.19)	0.93 (0.18- 1.7)
Belgium	211.46 (172.83- 246.59)	295.07 (258.01- 337.64)	-4.49 (-24.02- 25.03)	0.24 (0.09- 0.4)
Belize	0.49 (0.4- 0.61)	1.9 (1.65- 2.3)	30.18 (2.43- 69.8)	0.05 (-0.48- 0.59)
Benin	5.45 (3.24- 8.01)	11.58 (8.21- 16.41)	-6.1 (-34.63- 40.65)	-0.74 (-0.91- -0.56)
Bermuda	0.75 (0.55- 0.93)	0.55 (0.48- 0.63)	-61.58 (-70.54- -43.39)	-3.59 (-3.79- -3.39)
Bhutan	0.64 (0.38- 1.13)	1.23 (0.89- 1.68)	-9.96 (-49.33- 64.56)	-0.48 (-0.61- -0.36)

© 2021 Zhai Z et al. *JAMA Network Open*.

Bolivia	29.74 (16.94- 46.73)	42.72 (33.43- 52.93)	-40.76 (-62.3- -2.39)	-2.24 (-2.52- -1.96)
Bosnia and Herzegovina	11.13 (8.32- 15.84)	10.87 (9.26- 12.97)	-23.42 (-48.29- 8.29)	-1.38 (-1.7- -1.07)
Botswana	4.47 (3.05- 6.37)	7.89 (5.74- 11.54)	-14.81 (-44.17- 23.23)	-0.95 (-1.11- -0.8)
Brazil	547.65 (484.67- 681.37)	1077.7 (1002.89- 1145.76)	-16.18 (-34- -2.35)	-0.33 (-0.58- -0.08)
Brunei	0.54 (0.34- 0.78)	1.52 (1.34- 1.74)	-5.66 (-34.58- 47.84)	0.08 (-0.24- 0.41)
Bulgaria	13.05 (10.6- 16.28)	14.58 (12.9- 16.86)	13.05 (-12.03- 49.25)	-0.14 (-0.54- 0.25)
Burkina Faso	18.14 (9.25- 28.91)	22.92 (14.33- 35.67)	-34.52 (-52.89- -4.16)	-2.34 (-2.74- -1.95)
Burundi	10.78 (5.91- 20.53)	8.09 (5.01- 13.53)	-55.81 (-69.14- -28.39)	-4.17 (-4.59- -3.74)
Cambodia	21 (11.17- 38.5)	36.82 (28.04- 50.87)	-20.82 (-48.12- 26.2)	-1.03 (-1.12- -0.95)
Cameroon	20.19 (13.09- 28.22)	39.75 (29.48- 54.12)	-20.18 (-44.44- 31.39)	-1.53 (-1.9- -1.17)
Canada	399.25 (316.89- 467.14)	642.12 (581.25- 711.96)	-23.21 (-36.82- 1.11)	-0.91 (-0.96- -0.85)
Cape Verde	0.18 (0.13- 0.24)	0.42 (0.36- 0.5)	13.07 (-25.19- 69.14)	0.01 (-0.15- 0.18)
Central African Republic	4.74 (2.41- 9.22)	7.32 (4.4- 13.16)	-12.1 (-34.63- 32.75)	-0.72 (-0.86- -0.57)
Chad	8.2 (3.76- 12.98)	11.82 (6.8- 19.13)	-27.89 (-48.5- 11.99)	-1.47 (-1.61- -1.33)
Chile	45.29 (35.95- 54.39)	72.99 (63.36- 84.41)	-25.53 (-42.41- 0.52)	-0.83 (-0.98- -0.68)
China	1304.73 (994.53- 1731.33)	2876.51 (2641.65- 3053.12)	11.95 (-20.47- 48.06)	0.87 (0.41- 1.33)
Colombia	59.78 (48.82- 70.41)	129.75 (109.78- 154.99)	-18.82 (-36.57- 12.4)	-0.82 (-1.02- -0.63)
Comoros	0.52 (0.29- 0.93)	0.86 (0.59- 1.24)	-12.41 (-43.74- 45.41)	-1.23 (-1.47- -0.99)
Congo	6.17 (3.48- 10.07)	10.15 (7.24- 14.26)	-27.94 (-55.07- 13.37)	-1.89 (-2.2- -1.57)
Costa Rica	3.67 (3.3- 4.16)	8.48 (7.54- 9.71)	-20.13 (-32.58- -4.53)	-1.06 (-1.53- -0.59)
Cote d'Ivoire	18.14 (11.01- 26.4)	36.55 (25.05- 51.9)	-10.36 (-36.44- 31.98)	-1.19 (-1.52- -0.86)
Croatia	26.89 (22.03- 31.69)	84.79 (73.44- 99.32)	155.9 (101.08- 236.62)	5.39 (4.29- 6.5)
Cuba	20.68 (16.37- 24.72)	25.6 (22.07- 30.16)	-24.17 (-39.65- -2.8)	-1.11 (-1.4- -0.82)
Cyprus	11.06 (8.12- 14.85)	15.42 (12.95- 18.27)	-37.88 (-57.91- -7.28)	-1.84 (-2.14- -1.53)
Czech Republic	57.14 (46.26- 69.9)	63.16 (55.22- 73.15)	-22.15 (-40.2- 5.33)	-0.7 (-0.84- -0.56)

© 2021 Zhai Z et al. *JAMA Network Open*.

Democratic Republic of the Congo	37.61 (22.98- 64.41)	68.92 (46.11- 108.31)	-8.91 (-32.29- 26.14)	-0.69 (-0.89- -0.49)
Denmark	103.03 (89.53- 117.8)	152.87 (135.09- 176.45)	2.37 (-16.82- 31.06)	0.22 (0.11- 0.33)
Djibouti	0.33 (0.19- 0.58)	1.09 (0.73- 1.76)	10.99 (-32.38- 89.63)	-0.24 (-0.4- -0.08)
Dominica	0.18 (0.15- 0.21)	0.33 (0.28- 0.4)	34.83 (3.76- 76.7)	1.21 (1.13- 1.3)
Dominican Republic	5.01 (4.26- 6)	12.18 (10.04- 15.13)	8.55 (-16.52- 41.67)	0.36 (-0.13- 0.85)
Ecuador	12.5 (10.15- 14.66)	34.92 (30.16- 40.22)	18.91 (-7.14- 57.06)	0.97 (0.48- 1.46)
Egypt	35.87 (18.38- 60.35)	170.57 (84.98- 259.96)	157.87 (96.05- 250.65)	4.12 (3.56- 4.67)
El Salvador	5.8 (4.85- 7.05)	10.04 (7.89- 12.92)	-0.29 (-29.21- 34.24)	-0.28 (-0.49- -0.06)
Equatorial Guinea	0.8 (0.4- 1.65)	0.83 (0.51- 1.33)	-53.16 (-78.74- -5.23)	-3.56 (-3.98- -3.14)
Eritrea	4.64 (2.56- 8.74)	8.58 (5.84- 12.32)	-5.16 (-40.38- 58.83)	-0.98 (-1.06- -0.9)
Estonia	3.66 (3.17- 4.25)	6.23 (5.21- 7.43)	46.15 (11.32- 87.55)	1.26 (0.81- 1.71)
Ethiopia	97.5 (46.89- 196.76)	70.94 (40.16- 102.07)	-58.3 (-75.64- -12.45)	-4 (-4.32- -3.68)
Federated States of Micronesia	0.21 (0.16- 0.31)	0.36 (0.24- 0.48)	2.96 (-27.53- 39.33)	0.77 (0.71- 0.83)
Fiji	1.01 (0.82- 1.24)	2.72 (2.22- 3.35)	25.52 (-5.54- 69.69)	1.71 (1.23- 2.19)
Finland	82.39 (64.17- 96.98)	112.6 (98.66- 129.95)	-19.48 (-36.53- 9.79)	-0.48 (-0.67- -0.29)
France	1085.86 (889.85- 1277.62)	1889.79 (1651.3- 2170.13)	10.71 (-13.43- 48.52)	0.58 (0.22- 0.94)
Gabon	1.86 (1.11- 2.82)	2.77 (2.2- 4.2)	-17.1 (-47.91- 36.9)	-1.14 (-1.65- -0.62)
Georgia	7.58 (5.9- 9.09)	29.49 (25.83- 34.25)	342.75 (249.63- 473.48)	7.33 (6.06- 8.61)
Germany	1491.99 (1197.72- 1735.74)	2063.11 (1786.24- 2398.36)	-10.01 (-27.29- 16.7)	-0.11 (-0.23- 0.01)
Ghana	32.02 (25.46- 40.09)	42.63 (32.34- 55.27)	-40.24 (-58.5- -16.46)	-3.27 (-3.82- -2.71)
Greece	62.24 (48.79- 76.66)	87.29 (77.81- 97.51)	-0.29 (-22.27- 28.72)	-0.09 (-0.16- -0.02)
Greenland	0.44 (0.3- 0.57)	0.74 (0.65- 0.83)	-10.59 (-35.99- 37.48)	-0.12 (-0.62- 0.39)
Grenada	0.42 (0.34- 0.54)	0.44 (0.39- 0.51)	-52.05 (-64.28- -37.7)	-1.94 (-2.44- -1.43)
Guam	0.19 (0.15- 0.23)	0.59 (0.5- 0.7)	22.41 (-7.06- 63.5)	2.33 (1.7- 2.95)
Guatemala	8.55 (6.67- 11.67)	18.83 (16.23- 22.29)	-9.65 (-36.51- 22.71)	-1.25 (-1.51- -0.99)

© 2021 Zhai Z et al. *JAMA Network Open*.

Guinea	12.06 (5.97- 18.37)	17.83 (11.85- 27.4)	-4.68 (-35.69- 64.38)	-0.5 (-0.71- -0.29)
Guinea-Bissau	2.96 (1.52- 5.04)	3.74 (2.31- 5.94)	-22.86 (-48.21- 26.19)	-1.61 (-1.76- -1.46)
Guyana	2.21 (1.82- 2.65)	2.85 (2.4- 3.39)	-6.32 (-27.07- 19.75)	-0.64 (-1.16- -0.13)
Haiti	25.06 (11.87- 46.39)	34.84 (22.62- 56.09)	-31.15 (-51.87- 17.29)	-1.22 (-1.43- -1)
Honduras	6.19 (4.49- 9.15)	22.31 (16.17- 31.39)	36.53 (-12.59- 115.72)	1.31 (1.1- 1.53)
Hungary	47.42 (40.8- 56.48)	49.12 (43.54- 56.31)	-12.27 (-29.6- 9.38)	-0.68 (-0.9- -0.46)
Iceland	2.45 (2.07- 2.84)	5.29 (4.69- 6.07)	16.8 (-3.97- 47.29)	0.33 (0- 0.66)
India	1120.66 (706.43- 1958.5)	3030.61 (2545.97- 3875.25)	28.46 (-7.85- 73.64)	0.9 (0.69- 1.11)
Indonesia	238.34 (163.68- 335.56)	552.73 (476.85- 626.62)	23.88 (-4.8- 59.73)	1.01 (0.75- 1.26)
Iran	88.51 (68.09- 125.61)	578.52 (500.35- 692.66)	158.64 (67.71- 253.24)	5.21 (4.33- 6.09)
Iraq	31.14 (20.73- 44.35)	51.93 (44.95- 61.83)	-43.62 (-63.15- -14.04)	-2.68 (-3.48- -1.87)
Ireland	45.03 (29.08- 57.33)	48.46 (41.96- 56.58)	-37.6 (-53.89- 5.42)	-1.83 (-1.98- -1.68)
Israel	32.64 (28.16- 37.51)	49.33 (43.02- 57.04)	-33.67 (-47.49- -14.99)	-1.73 (-1.94- -1.51)
Italy	1408.81 (1012.71- 1733.32)	1746.21 (1555.42- 1954.99)	-18.61 (-37.97- 21.12)	-0.74 (-0.81- -0.66)
Jamaica	3.78 (3.22- 4.4)	6.51 (4.87- 8.52)	5.8 (-24.67- 50.44)	-1.29 (-2.16- -0.4)
Japan	634.67 (558.69- 698.27)	1538.77 (1440.29- 1669.82)	22.65 (7.75- 49.73)	1.32 (1.08- 1.55)
Jordan	5.6 (3.56- 7.87)	13.91 (10.98- 17.35)	-32.8 (-59.24- 14.72)	-2.09 (-2.48- -1.7)
Kazakhstan	46.32 (39.28- 56.29)	58.28 (51.77- 66.9)	0.89 (-21.22- 23.83)	-0.57 (-1.34- 0.2)
Kenya	14.24 (9.2- 20.55)	39.44 (31.09- 57.45)	20.69 (-2.68- 53.14)	-0.06 (-0.43- 0.31)
Kiribati	0.07 (0.06- 0.08)	0.16 (0.13- 0.2)	23.49 (-7.64- 70.21)	1.42 (1.21- 1.64)
Kuwait	1.21 (1.07- 1.46)	7.08 (5.7- 9.04)	72.11 (35.55- 131.5)	2.86 (1.73- 4)
Kyrgyzstan	3.38 (2.86- 3.98)	6.89 (5.77- 8.66)	45.67 (13.92- 91.86)	1.48 (1.15- 1.82)
Laos	9.96 (4.27- 18.91)	12.56 (8.8- 17.78)	-36.48 (-60.42- 19.6)	-1.95 (-2.07- -1.83)
Latvia	9.77 (8.38- 11.52)	11.62 (9.95- 13.66)	19.13 (-8.23- 51.06)	0.26 (-0.27- 0.78)
Lebanon	3.03 (1.97- 4.18)	8.47 (6.03- 13.23)	4.85 (-37.62- 61.01)	0.58 (0.38- 0.78)

© 2021 Zhai Z et al. *JAMA Network Open*.

Lesotho	11.12 (7.24- 16.07)	17.7 (13.39- 23.9)	36.63 (-9.33- 106.97)	1.29 (0.92- 1.66)
Liberia	2.87 (1.64- 4.46)	4.46 (3.09- 6.86)	-15.63 (-39.37- 26.66)	-1.04 (-1.34- -0.74)
Libya	2.41 (1.57- 3.33)	18.45 (10.64- 30.8)	204.65 (85.34- 374.5)	4.92 (4.57- 5.28)
Lithuania	18.17 (15.8- 21.11)	13.35 (11.99- 14.92)	-36.06 (-47.88- -21.93)	-2.32 (-2.83- -1.8)
Luxembourg	6.53 (5.04- 7.82)	9.62 (8.03- 11.77)	-13.16 (-34.78- 31.01)	-0.45 (-0.58- -0.33)
Macedonia	2.23 (1.72- 2.91)	2.37 (1.98- 2.84)	-29.66 (-51.03- -0.11)	-2.04 (-2.46- -1.62)
Madagascar	10.06 (5.85- 18.27)	15.8 (9.54- 26.54)	-19.52 (-38.74- 8.46)	-1.15 (-1.27- -1.04)
Malawi	7.23 (3.15- 14.27)	11.17 (6.82- 17.09)	-4.3 (-36.2- 82.24)	-1.44 (-1.94- -0.94)
Malaysia	19.71 (16.35- 25.21)	56 (46.19- 68.58)	-2.58 (-30.17- 30.71)	0.8 (0.64- 0.95)
Maldives	1.53 (0.66- 2.7)	1.67 (1.36- 2.13)	-63.08 (-81.28- -16.35)	-4.16 (-4.4- -3.93)
Mali	17.67 (8.68- 29.89)	15.59 (9.13- 24.08)	-50.39 (-64.35- -25.75)	-3.48 (-3.92- -3.05)
Malta	4.75 (3.95- 5.54)	10.7 (9.39- 12.24)	10.44 (-9.67- 37.58)	0.55 (0.39- 0.71)
Marshall Islands	0.06 (0.04- 0.1)	0.17 (0.11- 0.24)	18.96 (-14.59- 63.95)	1.23 (0.93- 1.53)
Mauritania	4.72 (3.09- 6.7)	4.58 (3.63- 5.77)	-46.04 (-63.02- -17.31)	-3.07 (-3.42- -2.72)
Mauritius	1.22 (1.02- 1.48)	1.82 (1.61- 2.15)	-32.54 (-48.29- -13.34)	-0.6 (-0.97- -0.22)
Mexico	163.32 (141.89- 186.98)	472.06 (451.45- 496.38)	31.61 (12.84- 51.31)	1.09 (0.86- 1.32)
Moldova	8.06 (6.7- 9.96)	8.54 (7.35- 9.89)	-4.43 (-25.22- 19.8)	-0.92 (-1.45- -0.38)
Mongolia	5.07 (3.57- 6.67)	4.54 (3.66- 5.64)	-61.62 (-73.3- -42.15)	-5.23 (-5.97- -4.48)
Montenegro	1.17 (1.03- 1.35)	1.98 (1.63- 2.33)	14.34 (-8.85- 40.34)	0.37 (-0.02- 0.77)
Morocco	14.94 (7.9- 25.28)	66.95 (43.31- 93.91)	134.24 (53.79- 253.17)	3.78 (3.41- 4.15)
Mozambique	14.75 (7.3- 28.34)	22.75 (14.65- 39.01)	-6.87 (-32.49- 46.64)	-0.6 (-0.91- -0.29)
Myanmar	378.11 (142.71- 773.51)	284.82 (201.41- 382.58)	-54.12 (-73.48- -7.07)	-3.17 (-3.43- -2.91)
Namibia	8.35 (5.8- 11.43)	11.39 (8.82- 15.28)	-21.93 (-46.89- 8.98)	-1.86 (-2.37- -1.34)
Nepal	22.16 (11.35- 42.79)	43.08 (29.79- 61.96)	5.82 (-32.06- 85.8)	-0.09 (-0.49- 0.31)
Netherlands	467.54 (394.44- 542.35)	680.42 (598.04- 773.37)	-15.02 (-30.83- 6.03)	-0.57 (-0.73- -0.4)

© 2021 Zhai Z et al. *JAMA Network Open*.

New Zealand	64.36 (43.88- 76.9)	96.87 (86.67- 107.66)	-20.38 (-36.54- 23.02)	-0.58 (-0.97- -0.2)
Nicaragua	1.76 (1.4- 2.22)	5.62 (4.58- 7.04)	21.07 (-15.68- 64.95)	0.8 (0.62- 0.98)
Niger	8.65 (4.05- 14.38)	12.04 (6.65- 20.17)	-38.74 (-54.99- -14.67)	-2.83 (-3.19- -2.46)
Nigeria	93.05 (62.68- 128.19)	111.8 (71.93- 165.23)	-32.97 (-56.85- 2.51)	-2.52 (-2.77- -2.27)
North Korea	25.44 (17.54- 34.95)	73.45 (54.8- 101.11)	49.86 (10.45- 105.85)	2.35 (2.04- 2.66)
Northern Mariana Islands	0.03 (0.02- 0.04)	0.07 (0.06- 0.09)	-18.8 (-36.8- 7.42)	0.58 (0.19- 0.97)
Norway	72.46 (60.27- 83.79)	85.2 (76.67- 96.5)	-18.61 (-32.9- 7.62)	-0.28 (-0.53- -0.02)
Oman	2.29 (1.49- 3.69)	10.44 (7.23- 14.64)	63.43 (-17.83- 193.39)	2.79 (2.37- 3.22)
Pakistan	86.54 (54.36- 140.67)	245.86 (180.52- 332.46)	43.92 (0.96- 112.3)	0.79 (0.42- 1.15)
Palestine	1.06 (0.68- 1.65)	2.53 (2.13- 3.14)	-22.99 (-53.92- 28.51)	-1.17 (-1.35- -1)
Panama	2.77 (2.41- 3.17)	6.72 (5.87- 7.8)	-13.3 (-28.96- 6.2)	0.09 (-0.1- 0.29)
Papua New Guinea	8.87 (4.94- 16.27)	23.52 (14.86- 40.55)	6.89 (-17.41- 44.68)	0.71 (0.53- 0.89)
Paraguay	4.18 (3.5- 4.96)	13.69 (10.96- 17.23)	25.15 (-7.15- 70.41)	1.29 (1.16- 1.42)
Peru	130.64 (105.04- 160.88)	70.79 (57.98- 84.74)	-77.04 (-83.19- -69.25)	-8.18 (-9.44- -6.89)
Philippines	91.48 (78.69- 113.57)	192.5 (160.69- 235.26)	4.78 (-18.74- 33.29)	-0.19 (-0.33- -0.05)
Poland	120.03 (98.9- 145.24)	391.02 (343.63- 444.32)	123 (71.01- 189.57)	4.02 (3.53- 4.52)
Portugal	47.95 (36.56- 58.93)	79.19 (68.97- 91.54)	6.15 (-21.33- 56.81)	0.14 (-0.42- 0.71)
Puerto Rico	9.19 (7.91- 10.88)	13.31 (11.65- 15.39)	-18.63 (-36.21- 3.86)	-0.91 (-1.17- -0.65)
Qatar	0.19 (0.13- 0.26)	3.03 (2.15- 4.25)	104.18 (30.51- 224.82)	3.22 (2.72- 3.72)
Romania	74.4 (64.09- 87.87)	96.16 (84.38- 110.6)	13.45 (-10.6- 40.9)	0.07 (-0.16- 0.3)
Russian Federation	676.87 (547.63- 889.74)	843.15 (767.81- 935.57)	4.19 (-18.78- 32.96)	-0.3 (-0.76- 0.17)
Rwanda	11.16 (6.31- 20.94)	9.33 (6.01- 13.42)	-49.8 (-69.57- -0.2)	-4.19 (-4.8- -3.58)
Saint Lucia	0.29 (0.24- 0.36)	0.48 (0.42- 0.56)	-30.7 (-47.21- -11.92)	-1.89 (-2.26- -1.51)
Saint Vincent and the Grenadines	0.18 (0.16- 0.22)	0.3 (0.26- 0.35)	-11.5 (-29.67- 9.35)	-0.73 (-0.99- -0.46)
Samoa	0.21 (0.16- 0.26)	0.32 (0.25- 0.42)	-11.89 (-30.81- 10.98)	0.2 (0.12- 0.27)

© 2021 Zhai Z et al. *JAMA Network Open*.

Sao Tome and Principe	0.1 (0.06- 0.15)	0.21 (0.17- 0.27)	21.24 (-18.81- 108.74)	0.11 (-0.13- 0.35)
Saudi Arabia	4.22 (2.53- 6.33)	23.71 (15.1- 34.02)	85.13 (-10.64- 283.2)	2.74 (2.42- 3.07)
Senegal	9.77 (5.7- 14.13)	20.16 (14.72- 27.7)	2.38 (-25.12- 47.07)	-0.48 (-0.62- -0.35)
Serbia	33.84 (27.67- 42.65)	48.53 (40.47- 59.07)	20.37 (-7.2- 55.13)	0.7 (0.26- 1.14)
Seychelles	0.17 (0.12- 0.24)	0.27 (0.21- 0.35)	-25.06 (-46.06- -1.85)	-1.12 (-1.22- -1.01)
Sierra Leone	4.28 (2.5- 6.55)	9.91 (6.69- 15.17)	31.55 (-11.81- 109.47)	0.91 (0.71- 1.1)
Singapore	12.97 (8.29- 17.08)	20.38 (18.16- 22.93)	-48.45 (-62.62- -15.15)	-2.34 (-2.58- -2.1)
Slovakia	19.22 (15.58- 24.48)	20.51 (17.18- 24.18)	-25.46 (-48.24- 1.79)	-0.99 (-1.14- -0.83)
Slovenia	16.71 (13.69- 20)	37.1 (32- 43.08)	43.6 (10.18- 87.51)	2.11 (1.43- 2.8)
Solomon Islands	0.6 (0.37- 1.05)	1.43 (1- 2.28)	-0.66 (-24.84- 36.99)	0.59 (0.46- 0.72)
Somalia	8.08 (3.3- 18.16)	16.28 (8.84- 29.24)	-7.88 (-41.56- 62.15)	-1.05 (-1.3- -0.81)
South Africa	165.97 (143.04- 193.32)	341.61 (305.14- 386.88)	10.97 (-2.28- 27.17)	-0.13 (-0.9- 0.64)
South Korea	55.5 (43.78- 66.32)	137.63 (120.08- 163.18)	-3.04 (-25.19- 38.47)	0.84 (0.5- 1.18)
South Sudan	3.38 (1.27- 7.85)	5.39 (2.71- 10.32)	4.97 (-30.55- 77.65)	-0.49 (-0.74- -0.23)
Spain	387.17 (317.24- 463.8)	556.64 (496.46- 626.75)	-13.48 (-31.86- 15.29)	-0.39 (-0.48- -0.29)
Sri Lanka	57.18 (44.61- 77.14)	99.41 (77.14- 130.1)	-11.95 (-43.77- 29.2)	-0.92 (-1.29- -0.55)
Sudan	9.47 (4.36- 18.82)	34.53 (22.33- 52.07)	99.08 (20.89- 237.64)	3.06 (2.77- 3.35)
Suriname	0.76 (0.64- 0.9)	1.44 (1.23- 1.7)	-5.82 (-26.4- 24.31)	-0.65 (-0.88- -0.42)
Swaziland	2.89 (1.98- 4.07)	5.17 (3.96- 6.69)	-4.94 (-35.96- 45.55)	0.14 (-0.37- 0.66)
Sweden	178.13 (147.96- 210.78)	180.6 (161.44- 205.9)	-29.86 (-43.46- -9.38)	-0.96 (-1.07- -0.85)
Switzerland	142.05 (114.21- 166.61)	203.85 (175.71- 239.37)	-11.96 (-30.99- 21.57)	0.14 (-0.12- 0.4)
Syria	9.61 (5.97- 14.81)	22.85 (17.96- 29.16)	-1.26 (-44.35- 72.47)	-0.34 (-0.57- -0.1)
Taiwan（China）	27.89 (22.01- 36.3)	56.04 (50.53- 63.44)	-11.9 (-34.53- 14.48)	-0.37 (-0.66- -0.08)
Tajikistan	11 (7.99- 14.5)	23.91 (19.65- 29.16)	20.99 (-13.29- 77.88)	-0.04 (-0.29- 0.21)
Tanzania	21.84 (11.88- 36.99)	40.85 (25.95- 57.52)	-3.77 (-31.96- 41.49)	-1.09 (-1.42- -0.77)

© 2021 Zhai Z et al. *JAMA Network Open*.

Thailand	270.04 (218.05- 346.78)	320.4 (261.27- 403.06)	-43.01 (-60.13- -20.83)	-3.26 (-3.84- -2.67)
The Bahamas	0.6 (0.5- 0.73)	1.17 (1.01- 1.41)	-16.33 (-34.26- 4.61)	-0.59 (-0.72- -0.45)
The Gambia	1.03 (0.6- 1.53)	2.29 (1.75- 3.13)	-1.22 (-30.31- 64.68)	-0.41 (-0.5- -0.33)
Timor-Leste	0.8 (0.45- 1.42)	1.79 (1.16- 2.41)	18.48 (-30.69- 120.27)	0.45 (0.14- 0.77)
Togo	5.21 (3.26- 7.55)	9.88 (7.1- 13.95)	-22.12 (-44.84- 19.92)	-1.67 (-1.92- -1.41)
Tonga	0.11 (0.09- 0.14)	0.18 (0.15- 0.22)	0.8 (-22.21- 30.3)	0.66 (0.59- 0.74)
Trinidad and Tobago	1.75 (1.54- 2.03)	3.21 (2.58- 3.98)	-7.05 (-30.6- 22.34)	-0.34 (-1.21- 0.54)
Tunisia	3.17 (2.53- 4.17)	17.48 (11.7- 26.14)	147.26 (55.25- 271.82)	3.8 (3.34- 4.26)
Turkey	779.43 (441.82- 1198.13)	814.36 (704.73- 959.32)	-52.12 (-70.71- -15.18)	-3.25 (-3.72- -2.78)
Turkmenistan	3.23 (2.63- 3.92)	7.67 (6.47- 9.21)	29.75 (2.84- 60.94)	0.67 (0.22- 1.12)
Uganda	10.86 (4.72- 19.47)	18.31 (11.41- 26.21)	-9.9 (-40.9- 51.82)	-1.25 (-1.49- -1.01)
Ukraine	208.34 (172.58- 258.56)	310.73 (274.67- 352)	53.53 (18.08- 97.02)	0.91 (0.51- 1.31)
United Arab Emirates	0.7 (0.38- 1.14)	17.56 (8.91- 31.6)	223.78 (92.37- 419.26)	5.01 (4.62- 5.4)
United Kingdom	2662.81 (2068.21- 3128.76)	3308.89 (3136.57- 3494.8)	-11.37 (-26.58- 14.48)	-0.42 (-0.48- -0.37)
United States	2451.43 (2196.81- 2645.88)	3172.34 (3043.92- 3309.45)	-16.56 (-25.29- -5.32)	-1.16 (-1.3- -1.03)
Uruguay	9.44 (8.38- 10.7)	14.57 (12.73- 16.84)	13.81 (-8.1- 39.08)	0.99 (0.64- 1.35)
Uzbekistan	20.19 (15.56- 27.65)	33.62 (28.2- 41.1)	-7.98 (-35.17- 27.35)	-1.29 (-1.98- -0.6)
Vanuatu	0.29 (0.17- 0.44)	0.94 (0.57- 1.51)	37.42 (-5.79- 103.17)	1.4 (1.27- 1.53)
Venezuela	21.24 (18.46- 24.9)	49.31 (40.99- 59.76)	-22.93 (-40.56- -0.74)	-1 (-1.29- -0.71)
Vietnam	97.59 (72.99- 134.03)	221.47 (181.33- 277.32)	0.33 (-29.29- 45.59)	0.16 (0.03- 0.28)
Virgin Islands, U.S.	0.58 (0.51- 0.69)	1.29 (1.06- 1.55)	16.05 (-9.93- 44.93)	0.76 (0.59- 0.93)
Yemen	8.02 (2.65- 19.16)	38.41 (20.39- 63.81)	101.76 (18.46- 328.41)	2.83 (2.51- 3.14)
Zambia	9.12 (5.23- 16.01)	17.87 (13.1- 25.26)	4.33 (-29.57- 64.17)	-1.37 (-1.97- -0.77)
Zimbabwe	5.03 (3.6- 7.81)	16.08 (12.74- 21.13)	92.62 (37.4- 165.99)	3.14 (1.93- 4.36)

CI: confidence interval; EAPC: estimated annual percentage change; UI: uncertainty interval.

© 2021 Zhai Z et al. *JAMA Network Open*.

eTable 6. Deaths and Age-Standardized Death Rates for Mesothelioma Among Countries and Territories in 1990 and 2017 and Temporal Trends From 1990 to 2017

Location	Death Cases No. (95% UI)		All ages percent change (%)	EAPC No. (95% CI)
	1990	2017	1990-2017	1990-2017
Afghanistan	31.59 (8.08- 81.27)	85.32 (32- 184.67)	133.77 (65.69- 383.31)	1.38 (0.93- 1.84)
Albania	2.25 (1.49- 3.32)	5.27 (3.26- 9.11)	78.71 (32.88- 130.69)	1.4 (1.13- 1.68)
Algeria	7.3 (5.35- 9.67)	41.99 (31.2- 57.93)	317.48 (180.53- 472.49)	3.99 (3.46- 4.51)
American Samoa	0.07 (0.06- 0.08)	0.16 (0.14- 0.19)	64.18 (31.22- 101.54)	1.85 (1.43- 2.27)
Andorra	1.24 (0.93- 1.52)	2.6 (1.92- 3.21)	3.91 (-23.84- 40.64)	-0.22 (-0.31- -0.13)
Angola	9.43 (4.56- 17.27)	18.76 (12.89- 26.34)	94.01 (25.82- 228.28)	-1.28 (-1.42- -1.13)
Antigua and Barbuda	0.07 (0.07- 0.08)	0.09 (0.08- 0.1)	-15.7 (-25.42- -6.28)	-2.35 (-2.66- -2.04)
Argentina	81.61 (74.99- 89.97)	223.55 (199.55- 250.69)	108.86 (80.52- 137.33)	2.77 (2.38- 3.16)
Armenia	14.71 (12.36- 18.86)	21.57 (20.11- 23.14)	27.25 (-1.98- 52.57)	-0.21 (-0.47- 0.04)
Australia	413.61 (315.96- 478.63)	804.05 (710.63- 899.61)	35.97 (13.17- 82.64)	-0.12 (-0.25- 0.02)
Austria	87.77 (67.04- 102.33)	116.6 (106.62- 128.01)	35.52 (11.53- 84.87)	0.07 (-0.19- 0.32)
Azerbaijan	4.62 (3.97- 5.22)	9.68 (8.31- 11.09)	52.35 (28.19- 81.44)	0.38 (-0.11- 0.87)
Bahrain	0.35 (0.29- 0.42)	2.7 (2.25- 3.24)	336.25 (225.51- 487.94)	3.22 (2.07- 4.38)
Bangladesh	111.09 (65.83- 200.97)	122.58 (91.11- 174.69)	45.17 (4.05- 116.41)	-2.95 (-3.08- -2.83)
Barbados	0.6 (0.52- 0.67)	0.84 (0.75- 0.93)	15.57 (-1.66- 38.18)	-0.97 (-1.19- -0.74)
Belarus	17.97 (14.8- 22.88)	28.85 (25.16- 33.33)	46.39 (18.03- 75.72)	1.06 (0.34- 1.79)
Belgium	179.83 (151.23- 202.2)	267.55 (241.11- 296.6)	42.8 (21.43- 81.76)	0.43 (0.27- 0.6)
Belize	0.39 (0.33- 0.46)	1.4 (1.28- 1.53)	85.37 (53.43- 126.61)	-0.21 (-0.75- 0.33)
Benin	3.98 (2.42- 5.72)	8.27 (6.02- 11.29)	63.98 (15.56- 135)	-0.66 (-0.84- -0.47)
Bermuda	0.62 (0.46- 0.71)	0.49 (0.45- 0.55)	-30.36 (-43.33- -2.65)	-3.43 (-3.62- -3.23)
Bhutan	0.47 (0.29- 0.8)	1 (0.73- 1.35)	150.82 (49.75- 335.07)	-0.15 (-0.25- -0.05)

© 2021 Zhai Z et al. *JAMA Network Open*.

Bolivia	21.83 (12.94- 33.62)	34.32 (27.17- 41.83)	49.56 (0.21- 135.81)	-1.97 (-2.24- -1.7)
Bosnia and Herzegovina	7.58 (5.97- 10.38)	8.19 (7.26- 9.3)	-18.42 (-42.58- 6.81)	-1.14 (-1.43- -0.84)
Botswana	3.87 (2.63- 5.4)	6.84 (5.08- 9.75)	14.08 (-24.62- 59.75)	-0.92 (-1.05- -0.78)
Brazil	409.92 (369.54- 502.23)	853.15 (798.29- 893.43)	47.95 (18.76- 69.64)	-0.32 (-0.57- -0.07)
Brunei	0.42 (0.27- 0.59)	1.18 (1.07- 1.31)	60 (13.52- 148.55)	0.04 (-0.26- 0.35)
Bulgaria	8.82 (7.46- 10.36)	10.55 (9.66- 11.5)	14.45 (-6.37- 38.36)	0 (-0.37- 0.37)
Burkina Faso	11.98 (6.25- 18.36)	15.45 (9.94- 23.75)	7.53 (-21.88- 55.32)	-2.16 (-2.55- -1.77)
Burundi	7.68 (4.4- 14.23)	6.04 (3.72- 9.73)	-16.69 (-37.68- 32.8)	-3.83 (-4.23- -3.43)
Cambodia	14.24 (8.27- 25.15)	27.19 (21.75- 36.66)	111.26 (43.34- 231.37)	-0.84 (-0.92- -0.75)
Cameroon	13.98 (9.38- 18.92)	28.09 (21.66- 37.32)	24.59 (-9.82- 95.85)	-1.35 (-1.69- -1)
Canada	383.95 (308.76- 442.3)	639.69 (583.6- 699.33)	14.83 (-3.34- 48.28)	-0.82 (-0.87- -0.77)
Cape Verde	0.14 (0.1- 0.18)	0.33 (0.29- 0.37)	92.87 (33.58- 174.22)	0.15 (-0.04- 0.35)
Central African Republic	3.34 (1.76- 6.41)	5.33 (3.29- 9.05)	11.92 (-15.66- 68.72)	-0.57 (-0.7- -0.44)
Chad	5.89 (2.72- 9.03)	8.38 (5.07- 13.25)	-2.11 (-29.79- 45.81)	-1.37 (-1.5- -1.24)
Chile	38.1 (31.33- 44.3)	63.54 (55.69- 72.46)	16.91 (-5.88- 50.37)	-0.86 (-1.01- -0.7)
China	1166.11 (910.56- 1536.04)	2780.02 (2556.75- 2950.74)	85.74 (32.65- 145.79)	1.08 (0.63- 1.53)
Colombia	45.11 (37.94- 51.3)	104.59 (89.97- 120.12)	67.3 (35.38- 117.75)	-0.82 (-1.02- -0.62)
Comoros	0.38 (0.22- 0.65)	0.65 (0.45- 0.95)	91.97 (31.94- 201.13)	-1.04 (-1.29- -0.8)
Congo	4.41 (2.6- 7.02)	7.24 (5.48- 9.92)	30.38 (-10.74- 96.89)	-1.74 (-2.04- -1.44)
Costa Rica	3.02 (2.78- 3.31)	7.14 (6.45- 7.84)	16.4 (2.28- 31.34)	-1.18 (-1.66- -0.7)
Cote d'Ivoire	12.21 (7.57- 17.24)	25.38 (18.28- 34.22)	57.75 (13.52- 135.73)	-1.07 (-1.39- -0.75)
Croatia	19.34 (16.47- 21.66)	68.01 (62.05- 74.76)	258.87 (206.61- 344.98)	5.74 (4.67- 6.83)
Cuba	15.52 (12.8- 17.83)	20.56 (18.07- 23.42)	-5.86 (-20.31- 11.55)	-1.03 (-1.32- -0.74)
Cyprus	9.43 (7.09- 12.51)	13.28 (11.51- 15.51)	-1.21 (-30.88- 41.73)	-1.84 (-2.17- -1.52)
Czech Republic	40.89 (34.07- 48.46)	49.22 (44.71- 54.17)	40.48 (12.91- 78.02)	-0.41 (-0.56- -0.27)

© 2021 Zhai Z et al. *JAMA Network Open*.

Democratic Republic of the Congo	26.99 (16.68- 44.27)	50.64 (34.67- 75.93)	48.14 (15.68- 100.94)	-0.6 (-0.81- -0.39)
Denmark	86.12 (77.17- 94.53)	133.29 (119.58- 149.49)	71.55 (49.07- 104.82)	0.43 (0.33- 0.53)
Djibouti	0.23 (0.13- 0.4)	0.82 (0.56- 1.29)	117.87 (50.37- 238.46)	-0.06 (-0.22- 0.09)
Dominica	0.15 (0.13- 0.18)	0.28 (0.25- 0.31)	56.13 (28.82- 91.73)	1.1 (1.01- 1.2)
Dominican Republic	3.53 (3.09- 4.06)	8.93 (7.49- 10.54)	47.87 (23.13- 81.43)	0.31 (-0.14- 0.77)
Ecuador	9.43 (7.78- 10.75)	27.91 (24.8- 31.03)	73.69 (42.56- 122.57)	1.02 (0.55- 1.5)
Egypt	25.72 (13.46- 42.12)	128.76 (62.99- 194.78)	326.77 (243.22- 471.21)	4.28 (3.71- 4.85)
El Salvador	4.3 (3.73- 5.1)	7.57 (6.09- 9.24)	39.65 (8.22- 74.82)	-0.32 (-0.52- -0.13)
Equatorial Guinea	0.57 (0.3- 1.14)	0.61 (0.39- 0.97)	9.59 (-44.51- 92.87)	-3.26 (-3.67- -2.84)
Eritrea	3.32 (1.87- 6.11)	6.18 (4.22- 8.47)	238.83 (119.77- 457.08)	-0.83 (-0.9- -0.76)
Estonia	2.64 (2.37- 3)	5.03 (4.29- 5.87)	140.49 (93.29- 189.79)	1.56 (1.12- 2)
Ethiopia	67.4 (33.3- 133.04)	53.27 (30.12- 77.36)	24.3 (-25.76- 163.88)	-3.63 (-3.94- -3.32)
Federated States of Micronesia	0.16 (0.12- 0.23)	0.27 (0.19- 0.35)	73.24 (26.45- 122.61)	0.81 (0.76- 0.87)
Fiji	0.76 (0.63- 0.9)	2.2 (1.81- 2.64)	70.13 (34.93- 113.12)	1.89 (1.43- 2.36)
Finland	68.54 (55.28- 77.23)	99.79 (89.7- 111.27)	31.83 (10.7- 73.46)	-0.34 (-0.51- -0.18)
France	939.18 (791.88- 1067.68)	1673.69 (1520.91- 1860.47)	62.05 (34.24- 105.96)	0.61 (0.28- 0.94)
Gabon	1.43 (0.87- 2.11)	2.13 (1.75- 3.34)	34.97 (-10.72- 114.2)	-1.03 (-1.5- -0.56)
Georgia	5.32 (4.26- 6.19)	22.59 (20.86- 24.3)	344.28 (277.59- 446.89)	7.58 (6.29- 8.89)
Germany	1168.13 (952.78- 1317.6)	1681.66 (1503.75- 1898.32)	39.43 (19.82- 74.42)	0.04 (-0.08- 0.17)
Ghana	22.33 (18.41- 26.94)	29.36 (22.96- 35.91)	-5.64 (-31.09- 25.07)	-3.31 (-3.89- -2.73)
Greece	52.33 (41.77- 63.89)	77.99 (71.31- 85.24)	8.71 (-12.05- 39.61)	-0.1 (-0.19- -0.01)
Greenland	0.35 (0.24- 0.45)	0.68 (0.6- 0.77)	88.71 (36.84- 187.26)	0.41 (-0.15- 0.97)
Grenada	0.31 (0.27- 0.39)	0.35 (0.32- 0.38)	-26.43 (-42.26- -12.67)	-1.84 (-2.36- -1.31)
Guam	0.15 (0.12- 0.18)	0.49 (0.42- 0.57)	46.35 (15.15- 88.36)	2.17 (1.56- 2.78)
Guatemala	6.04 (4.91- 8.02)	13.63 (12.16- 15.22)	73.75 (26.13- 120.5)	-1.28 (-1.54- -1.02)

© 2021 Zhai Z et al. *JAMA Network Open*.

Guinea	8.36 (4.15- 12.51)	12.76 (8.86- 18.89)	55.39 (6.02- 159.21)	-0.3 (-0.49- -0.1)
Guinea-Bissau	1.93 (1.04- 3.21)	2.48 (1.59- 3.86)	43.68 (1- 133.82)	-1.42 (-1.58- -1.27)
Guyana	1.45 (1.28- 1.65)	1.98 (1.73- 2.24)	30.11 (9.55- 54.29)	-0.54 (-1.02- -0.06)
Haiti	16.62 (8.44- 29.35)	24.51 (16.91- 37.48)	37.04 (3.27- 123.24)	-0.94 (-1.14- -0.74)
Honduras	4.3 (3.11- 6.14)	16.31 (11.84- 23.45)	133.16 (59.19- 245.19)	1.4 (1.18- 1.61)
Hungary	32.69 (29.08- 37.35)	37.01 (34.22- 40.22)	34.7 (14.62- 55.77)	-0.36 (-0.57- -0.16)
Iceland	2.1 (1.84- 2.34)	4.67 (4.25- 5.1)	76.85 (52.23- 112.44)	0.43 (0.11- 0.74)
India	843.67 (549.01- 1441.66)	2422.43 (2063.88- 3045.47)	142.9 (74.88- 226.49)	1.01 (0.81- 1.21)
Indonesia	160.27 (115.57- 219.85)	412.73 (367.17- 461.51)	111.13 (65.05- 175.15)	1.28 (1.02- 1.53)
Iran	70.35 (56.25- 96.33)	493.64 (448.65- 530.99)	490.01 (301.58- 676.41)	5.38 (4.5- 6.27)
Iraq	23.55 (16.3- 32.84)	39.24 (35.23- 43.71)	27.94 (-12.58- 88.84)	-2.6 (-3.4- -1.8)
Ireland	38.8 (25.86- 47.77)	43 (38.41- 48.33)	12.61 (-12.8- 80.49)	-1.73 (-1.88- -1.59)
Israel	26.71 (23.85- 29.55)	41.93 (38.03- 47.01)	-0.14 (-14.25- 20.68)	-1.64 (-1.85- -1.42)
Italy	1253.29 (929.57- 1479.14)	1714.2 (1556.85- 1900.52)	18.98 (-3.25- 68.37)	-0.51 (-0.61- -0.4)
Jamaica	3.19 (2.77- 3.66)	5.08 (3.87- 6.28)	3.75 (-22.14- 36.21)	-1.53 (-2.39- -0.67)
Japan	579.24 (516.81- 617.29)	1555.82 (1477.79- 1665.87)	60.3 (45.2- 88.14)	1.43 (1.19- 1.66)
Jordan	4.33 (2.84- 5.99)	11.54 (9.34- 13.74)	54.06 (-1.33- 150.93)	-1.75 (-2.12- -1.38)
Kazakhstan	31.73 (27.88- 37.51)	41.89 (38.43- 45.77)	26.99 (2.67- 47.31)	-0.35 (-1.07- 0.38)
Kenya	10.39 (6.78- 14.35)	29.52 (22.83- 43.58)	82.49 (49.69- 131.07)	0.07 (-0.31- 0.44)
Kiribati	0.05 (0.04- 0.06)	0.11 (0.09- 0.14)	81.89 (42.45- 142.8)	1.42 (1.23- 1.61)
Kuwait	0.9 (0.83- 0.97)	5.72 (4.79- 7.09)	334.17 (265.22- 440.37)	3.07 (1.87- 4.28)
Kyrgyzstan	2.2 (1.94- 2.49)	4.63 (4.11- 5.4)	128.82 (90.52- 182.65)	1.72 (1.37- 2.06)
Laos	6.57 (3.2- 11.97)	8.84 (6.62- 11.97)	75.35 (13.66- 224.96)	-1.71 (-1.82- -1.6)
Latvia	6.98 (6.25- 7.92)	8.97 (7.85- 10.33)	56.07 (29.01- 83.14)	0.42 (-0.08- 0.91)
Lebanon	2.12 (1.42- 2.79)	6.45 (4.64- 9.5)	101.99 (25.74- 194.22)	0.96 (0.74- 1.18)

© 2021 Zhai Z et al. *JAMA Network Open*.

Lesotho	9.56 (6.35- 13.33)	14.58 (11.37- 19.21)	4 (-27.94- 50.25)	1.13 (0.78- 1.47)
Liberia	1.96 (1.15- 2.9)	3.14 (2.2- 4.64)	102.2 (44.85- 202.51)	-0.66 (-0.95- -0.37)
Libya	1.77 (1.19- 2.42)	13.84 (8.12- 22.92)	322.31 (185.94- 523.2)	5.13 (4.75- 5.5)
Lithuania	12.87 (11.48- 14.58)	10.28 (9.42- 11.19)	-23.12 (-34.66- -12.34)	-2.19 (-2.68- -1.69)
Luxembourg	5.5 (4.37- 6.24)	8.35 (7.18- 9.9)	31.92 (7.63- 88.57)	-0.36 (-0.49- -0.22)
Macedonia	1.49 (1.21- 1.86)	1.7 (1.47- 1.93)	-13.6 (-37.04- 15.23)	-1.97 (-2.37- -1.56)
Madagascar	7.2 (4.33- 12.56)	11.45 (6.99- 18.92)	28.58 (3.1- 66.95)	-1.01 (-1.12- -0.9)
Malawi	5.23 (2.37- 9.75)	8.65 (5.42- 12.61)	119.7 (52.12- 309.33)	-1.16 (-1.63- -0.68)
Malaysia	15.03 (12.96- 18.46)	43.82 (37.3- 51.55)	49.7 (13.94- 87.38)	0.75 (0.62- 0.89)
Maldives	1.04 (0.5- 1.78)	1.3 (1.11- 1.54)	42.37 (-26.09- 230.81)	-3.83 (-4.07- -3.59)
Mali	11.78 (6.03- 19.16)	10.89 (6.57- 16.35)	-17.24 (-37.47- 19.79)	-3.19 (-3.61- -2.77)
Malta	4.02 (3.41- 4.53)	9.33 (8.46- 10.21)	56.26 (34.21- 89.99)	0.52 (0.35- 0.69)
Marshall Islands	0.05 (0.03- 0.08)	0.13 (0.09- 0.18)	89.74 (44.3- 147.8)	1.23 (0.94- 1.51)
Mauritania	3.31 (2.21- 4.55)	3.27 (2.66- 3.94)	10.35 (-19.91- 60.78)	-2.91 (-3.25- -2.57)
Mauritius	0.94 (0.8- 1.13)	1.46 (1.33- 1.6)	2.75 (-18.3- 24.33)	-0.69 (-1.08- -0.3)
Mexico	125.43 (110.59- 143.23)	379.99 (366.63- 393.93)	83.96 (57.37- 110.59)	1.15 (0.93- 1.38)
Moldova	5.15 (4.52- 6.05)	5.59 (5.06- 6.14)	13.62 (-4.26- 31.66)	-0.96 (-1.5- -0.43)
Mongolia	3.3 (2.45- 4.15)	3.01 (2.55- 3.58)	-15.72 (-38.44- 21.08)	-4.98 (-5.69- -4.27)
Montenegro	0.79 (0.72- 0.88)	1.48 (1.25- 1.69)	25.63 (4.23- 46.5)	0.66 (0.31- 1.01)
Morocco	10.35 (5.94- 16.76)	51.82 (35.21- 71.62)	319.24 (200.44- 490.67)	4.19 (3.8- 4.59)
Mozambique	10.77 (5.54- 19.81)	16.96 (11.38- 27.32)	45.03 (7.62- 121.8)	-0.46 (-0.74- -0.18)
Myanmar	239.11 (97.43- 471.53)	207.33 (154.85- 266.2)	2.55 (-39.68- 106.92)	-2.74 (-2.97- -2.51)
Namibia	7.32 (5.17- 9.8)	10.08 (8.1- 12.93)	0.83 (-29.74- 36.88)	-1.83 (-2.34- -1.32)
Nepal	16.24 (8.57- 30.88)	34.87 (24.23- 49.08)	152.48 (69.65- 325.2)	0.18 (-0.19- 0.55)
Netherlands	390.35 (342.98- 432.65)	617.89 (569.46- 676.17)	33.54 (16.73- 55.77)	-0.23 (-0.37- -0.08)

© 2021 Zhai Z et al. *JAMA Network Open*.

New Zealand	64.6 (44.07- 76.96)	96.61 (86.65- 107.42)	22.49 (-2.56- 89.13)	-0.72 (-0.95- -0.49)
Nicaragua	1.22 (1.01- 1.49)	4.02 (3.41- 4.71)	176.68 (106.41- 252.08)	0.75 (0.56- 0.93)
Niger	5.79 (2.79- 9.19)	8.4 (4.8- 13.66)	40.06 (4.95- 94.26)	-2.64 (-3.01- -2.27)
Nigeria	68.17 (46.7- 92.32)	79.55 (52.97- 114.57)	-3.01 (-36.05- 41.74)	-2.44 (-2.69- -2.19)
North Korea	17.87 (12.95- 24.09)	54.14 (41.97- 71.82)	60.9 (33.3- 95.97)	2.36 (2.06- 2.65)
Northern Mariana Islands	0.02 (0.02- 0.03)	0.06 (0.05- 0.07)	29.68 (6.54- 63.24)	0.66 (0.25- 1.07)
Norway	62.59 (54.33- 68.65)	75.62 (70.3- 82.85)	32.2 (13.18- 65.37)	-0.12 (-0.38- 0.14)
Oman	1.64 (1.09- 2.61)	7.53 (5.45- 9.93)	252.9 (90.41- 472.25)	2.96 (2.51- 3.4)
Pakistan	62.11 (40.3- 96.16)	175.39 (133.79- 230.15)	128.12 (70.72- 218.55)	0.96 (0.62- 1.3)
Palestine	0.79 (0.53- 1.19)	1.84 (1.6- 2.1)	72.32 (7.62- 170.68)	-1.24 (-1.44- -1.03)
Panama	2.19 (1.93- 2.45)	5.52 (5- 6.07)	34 (13.95- 56.79)	0.07 (-0.13- 0.28)
Papua New Guinea	6.11 (3.57- 10.95)	16.42 (10.71- 27.67)	44.04 (19.35- 81.09)	0.83 (0.66- 1)
Paraguay	3.31 (2.86- 3.79)	10.86 (8.99- 13.01)	80.06 (45.47- 118.49)	1.22 (1.1- 1.34)
Peru	103.52 (84.79- 125.63)	59.17 (49.22- 69.86)	-44.55 (-57.76- -30.89)	-8.2 (-9.49- -6.89)
Philippines	63.05 (57.21- 69.66)	137.33 (118.06- 158.2)	26.1 (9.62- 43.78)	-0.17 (-0.32- -0.02)
Poland	83.56 (70.78- 100.58)	291.96 (266.05- 318.51)	244.3 (172.84- 325.48)	4.24 (3.7- 4.79)
Portugal	39.16 (30.7- 47.89)	68.73 (61.53- 76.6)	56.19 (20.74- 114.99)	0.13 (-0.43- 0.7)
Puerto Rico	7.85 (6.9- 8.82)	11.8 (10.54- 13.16)	12.43 (-7.28- 36.66)	-1.02 (-1.3- -0.74)
Qatar	0.13 (0.1- 0.18)	2.15 (1.63- 2.82)	425.14 (247.02- 718.81)	3.09 (2.59- 3.59)
Romania	50.01 (45.1- 56.24)	70.69 (64.84- 76.98)	34.44 (14.92- 53.15)	0.3 (0.09- 0.52)
Russian Federation	463.07 (395.34- 590.07)	610.81 (583.97- 640.46)	17.72 (-5.3- 38.04)	-0.14 (-0.56- 0.27)
Rwanda	8.03 (4.76- 14.54)	7.2 (4.61- 10.2)	48.24 (-7.35- 194.27)	-3.8 (-4.36- -3.23)
Saint Lucia	0.21 (0.17- 0.25)	0.36 (0.33- 0.39)	14.76 (-6.58- 38.64)	-1.86 (-2.23- -1.49)
Saint Vincent and the Grenadines	0.14 (0.12- 0.16)	0.23 (0.21- 0.26)	18.6 (0.58- 37.43)	-0.65 (-0.91- -0.39)
Samoa	0.16 (0.13- 0.2)	0.26 (0.2- 0.33)	14.26 (-4.97- 37.37)	0.23 (0.17- 0.29)

© 2021 Zhai Z et al. *JAMA Network Open*.

Sao Tome and Principe	0.07 (0.05- 0.11)	0.15 (0.12- 0.18)	107.29 (44.81- 250.24)	0.26 (0.07- 0.46)
Saudi Arabia	3.16 (1.95- 4.64)	16.93 (11.17- 22.39)	359.71 (120.81- 797.61)	2.82 (2.48- 3.16)
Senegal	7.15 (4.23- 10.08)	14.74 (10.82- 19.37)	104.13 (57.04- 186.85)	-0.44 (-0.58- -0.31)
Serbia	22.44 (18.89- 27.03)	35.28 (30.64- 39.65)	22.84 (1.45- 48.54)	0.96 (0.55- 1.37)
Seychelles	0.13 (0.1- 0.18)	0.21 (0.16- 0.25)	10.66 (-16.38- 37.32)	-1.12 (-1.23- -1.01)
Sierra Leone	3.11 (1.87- 4.65)	6.92 (4.91- 10.17)	122.03 (46.66- 237.47)	0.93 (0.78- 1.07)
Singapore	11.86 (7.69- 15.38)	19.36 (17.36- 21.54)	12.53 (-16.86- 82.38)	-2.32 (-2.56- -2.08)
Slovakia	13.15 (10.96- 15.84)	14.9 (12.96- 16.95)	18.42 (-9.65- 52.28)	-0.78 (-0.93- -0.63)
Slovenia	12 (10.09- 13.78)	29.35 (26.14- 32.99)	124.5 (83.91- 185.12)	2.43 (1.77- 3.09)
Solomon Islands	0.44 (0.28- 0.74)	1.05 (0.75- 1.62)	47.85 (19.25- 90.22)	0.65 (0.52- 0.77)
Somalia	5.57 (2.38- 11.73)	11.74 (6.35- 20.19)	70.91 (18.54- 207.74)	-0.88 (-1.11- -0.65)
South Africa	133.97 (115.84- 156.56)	289.37 (258.27- 324.48)	27.29 (12.7- 45.32)	0.04 (-0.71- 0.8)
South Korea	44.31 (35.97- 51.56)	125.14 (110.28- 147.17)	123.7 (74.01- 213.61)	1.01 (0.7- 1.33)
South Sudan	2.45 (0.97- 5.32)	3.91 (2- 7.18)	36.16 (-6.42- 133.92)	-0.41 (-0.66- -0.16)
Spain	313.18 (266.38- 360.04)	491.42 (451.29- 538.32)	24.92 (4.29- 56.09)	-0.16 (-0.25- -0.08)
Sri Lanka	43.47 (35.22- 56.17)	79.07 (61.99- 96.41)	51.96 (2.35- 104.88)	-0.93 (-1.31- -0.55)
Sudan	6.64 (3.28- 12.94)	26.08 (17.24- 38.98)	354.62 (192.23- 645.67)	3.48 (3.17- 3.79)
Suriname	0.53 (0.46- 0.6)	1.05 (0.93- 1.17)	22.98 (5.23- 45.49)	-0.57 (-0.8- -0.34)
Swaziland	2.43 (1.71- 3.3)	4.28 (3.39- 5.22)	5.04 (-24.93- 49.3)	-0.01 (-0.5- 0.49)
Sweden	154.7 (131.56- 176.05)	164.75 (152.15- 178.85)	9.37 (-7.08- 32.98)	-0.76 (-0.88- -0.64)
Switzerland	120.05 (99.82- 134.52)	183.29 (164.17- 208.07)	47.96 (24.77- 95.93)	0.37 (0.12- 0.62)
Syria	6.97 (4.53- 10.49)	17.5 (14.13- 21.78)	38.68 (-16.95- 132.98)	-0.17 (-0.41- 0.07)
Taiwan (China)	21.72 (17.53- 28.41)	47.05 (43.7- 50.64)	25.96 (-5.34- 57.98)	-0.32 (-0.62- -0.01)
Tajikistan	5.81 (4.47- 6.98)	13.13 (11.79- 14.67)	84.6 (47.69- 146.4)	0.17 (-0.08- 0.42)
Tanzania	15.95 (8.89- 26.21)	31.15 (19.78- 44.13)	87.5 (33.67- 169.96)	-0.87 (-1.17- -0.57)

© 2021 Zhai Z et al. *JAMA Network Open*.

Thailand	192.31 (162.61- 237.76)	241.44 (208.94- 278.77)	-15.11 (-36.09- 4.29)	-3.27 (-3.78- -2.77)
The Bahamas	0.44 (0.38- 0.5)	0.87 (0.79- 0.97)	9.58 (-5.93- 28.96)	-0.66 (-0.8- -0.53)
The Gambia	0.72 (0.43- 1.04)	1.68 (1.33- 2.21)	58.52 (14.25- 150.16)	-0.33 (-0.42- -0.25)
Timor-Leste	0.53 (0.32- 0.89)	1.37 (0.96- 1.84)	175.02 (64.52- 400.1)	0.65 (0.35- 0.95)
Togo	3.47 (2.24- 4.81)	6.87 (5.27- 9.37)	53.01 (12.14- 124.2)	-1.44 (-1.68- -1.2)
Tonga	0.09 (0.08- 0.11)	0.15 (0.12- 0.18)	29.52 (4.89- 58.74)	0.66 (0.57- 0.75)
Trinidad and Tobago	1.4 (1.26- 1.58)	2.56 (2.08- 3.1)	32.83 (12.37- 56.5)	-0.47 (-1.35- 0.43)
Tunisia	2.22 (1.78- 2.96)	14.09 (9.35- 21.47)	322.63 (187.97- 505.27)	4.11 (3.64- 4.58)
Turkey	564.15 (332.06- 861.23)	661.15 (588.06- 748.33)	7.84 (-32.18- 82.11)	-2.88 (-3.35- -2.42)
Turkmenistan	2.09 (1.8- 2.44)	5.1 (4.53- 5.65)	97 (65.64- 125.81)	0.83 (0.39- 1.27)
Uganda	7.88 (3.52- 13.72)	13.96 (8.76- 19.85)	125.64 (51.71- 269.47)	-0.96 (-1.19- -0.74)
Ukraine	142.81 (120.14- 172.65)	214.47 (199.76- 229.24)	37.55 (11.62- 67.14)	0.89 (0.5- 1.27)
United Arab Emirates	0.47 (0.25- 0.76)	12.11 (6.08- 21.34)	396.54 (237.69- 637.75)	5.15 (4.72- 5.58)
United Kingdom	2137.06 (1676.43- 2508.33)	2802.57 (2708.68- 2911.49)	39.27 (15.96- 78.35)	-0.14 (-0.22- -0.07)
United States	2389.62 (2137.49- 2580.57)	3147.07 (3024.33- 3280.45)	-1.37 (-11.7- 12.1)	-1.07 (-1.21- -0.94)
Uruguay	8.35 (7.53- 9.19)	13.12 (11.65- 14.72)	40.97 (22.6- 60.69)	0.89 (0.54- 1.24)
Uzbekistan	12.68 (10.17- 17.11)	21.49 (18.75- 24.33)	6.21 (-20.05- 35.81)	-1.07 (-1.72- -0.42)
Vanuatu	0.22 (0.13- 0.32)	0.73 (0.45- 1.17)	67.89 (28.76- 120.67)	1.49 (1.36- 1.62)
Venezuela	16.01 (14.26- 18.3)	37.81 (32.37- 44.25)	24.59 (3.06- 45.21)	-1.06 (-1.36- -0.76)
Vietnam	73.67 (55.27- 96.53)	173.67 (147.88- 206.5)	68.38 (26.72- 124.72)	0.22 (0.08- 0.37)
Virgin Islands, U.S.	0.45 (0.4- 0.51)	1.08 (0.88- 1.24)	26.14 (10.17- 41.78)	0.79 (0.61- 0.98)
Yemen	5.28 (1.89- 12.26)	27.16 (15.38- 45.34)	368.79 (200.7- 851.9)	3.26 (2.93- 3.59)
Zambia	6.26 (3.73- 10.71)	13.26 (9.72- 17.96)	105.83 (44.47- 215.65)	-0.98 (-1.57- -0.39)
Zimbabwe	3.87 (2.87- 5.82)	12.21 (9.9- 15.25)	118.98 (59.45- 203.2)	2.99 (1.82- 4.18)

ASDR: age-standardized death rate; CI: confidence interval; EAPC: estimated annual percentage change; UI: uncertainty interval.

© 2021 Zhai Z et al. *JAMA Network Open*.

eTable 7. DALYs and Age-Standardized DALY Rates for Mesothelioma Among Countries and Territories in 1990 and 2017 and Temporal Trends From 1990 to 2017

Location	DALY Cases No. (95% UI)		All ages percent change (%)	EAPC No. (95% CI)
	1990	2017	1990-2017	1990-2017
Afghanistan	1131.03 (250.12- 3086.77)	3503.26 (1133.62- 8007.6)	138.02 (62.08- 440.67)	1.09 (0.61- 1.57)
Albania	68.72 (44.6- 103.55)	133.17 (80.08- 237.6)	137.77 (69.49- 214.73)	1.25 (0.98- 1.51)
Algeria	249.84 (177.56- 330.19)	1227.33 (925.03- 1675.52)	344.3 (193.68- 531.79)	3.49 (2.99- 3.99)
American Samoa	2.08 (1.79- 2.43)	4.85 (4.18- 5.56)	87.01 (48.01- 130.34)	1.84 (1.43- 2.26)
Andorra	26.36 (19.71- 32.69)	50.24 (36.43- 61.87)	22.63 (-10.58- 68.75)	-0.43 (-0.5- -0.36)
Angola	323.51 (150.88- 608.19)	613.91 (419.76- 881.4)	95.39 (23.39- 250.37)	-1.6 (-1.76- -1.43)
Antigua and Barbuda	2.02 (1.86- 2.19)	2.5 (2.26- 2.73)	-14.56 (-25.36- -4.04)	-2.5 (-2.84- -2.16)
Argentina	2180.64 (2001.61- 2391.34)	5344.75 (4741.17- 6013.8)	113.41 (82.87- 144.84)	2.43 (2.07- 2.79)
Armenia	424.5 (346.54- 550.98)	524.86 (487.09- 566.75)	58.17 (20.12- 94.66)	-0.38 (-0.64- -0.11)
Australia	8746.02 (6638.4- 10082.03)	13858.23 (12226.44- 15603.39)	22.24 (0.93- 66.04)	-0.79 (-0.91- -0.68)
Austria	1884.81 (1434.09- 2199.6)	2290.22 (2091.31- 2531.65)	26.28 (2.96- 73.62)	-0.19 (-0.43- 0.05)
Azerbaijan	143.22 (125.98- 158.36)	291.25 (248.21- 334.26)	91.7 (57.73- 126.54)	0.3 (-0.14- 0.74)
Bahrain	9.72 (8.15- 11.54)	69.93 (57.96- 84.32)	230.88 (145.92- 350.27)	2.89 (1.84- 3.94)
Bangladesh	3418.21 (1970.89- 6265.25)	3479.76 (2487.87- 4920.8)	81.04 (23.87- 195.15)	-2.98 (-3.09- -2.86)
Barbados	15.96 (14.27- 17.76)	21.71 (19.41- 24.24)	16.75 (-0.82- 37.61)	-1.04 (-1.24- -0.84)
Belarus	481.9 (390.43- 636.8)	704.13 (602.03- 857.53)	56.51 (25.94- 88.37)	0.86 (0.1- 1.63)
Belgium	3791.85 (3203.76- 4238.96)	4889.4 (4399.33- 5447.7)	29.83 (8.86- 65.03)	0.01 (-0.15- 0.16)
Belize	11.85 (9.98- 14.18)	46.31 (42.26- 50.61)	119.58 (77.93- 169.96)	0.03 (-0.51- 0.58)
Benin	122.75 (74.8- 178.45)	269.6 (193.09- 384.65)	83.4 (21.68- 176.34)	-0.82 (-1.02- -0.63)
Bermuda	17.48 (13.04- 20.22)	11.03 (9.91- 12.26)	-36.15 (-47.7- -9.61)	-3.86 (-4.07- -3.64)
Bhutan	15.13 (8.99- 26.42)	27.44 (19.6- 37.01)	164.47 (48.86- 387)	-0.63 (-0.76- -0.51)

© 2021 Zhai Z et al. *JAMA Network Open*.

Bolivia	639.25 (362.84- 997.64)	874.05 (680.67- 1066.77)	75.59 (11.99- 199.89)	-2.3 (-2.56- -2.05)
Bosnia and Herzegovina	222.01 (176.3- 303.25)	205 (182.93- 231.15)	-0.87 (-30.06- 30.57)	-1.22 (-1.52- -0.91)
Botswana	102.41 (68.27- 147.56)	168.59 (122.46- 248.39)	20.25 (-21.88- 71.25)	-1.33 (-1.52- -1.13)
Brazil	12431.98 (11165- 15462.84)	23640.57 (22119.57- 24885.88)	80.72 (40.73- 110.5)	-0.28 (-0.48- -0.08)
Brunei	13 (8.13- 18.17)	35.46 (31.82- 39.5)	63.55 (14.38- 158.67)	0.09 (-0.22- 0.39)
Bulgaria	263.9 (222.63- 308.92)	285.37 (260.81- 312.63)	26.66 (4.02- 54.11)	-0.07 (-0.45- 0.3)
Burkina Faso	413.06 (213.42- 648.47)	535.2 (332.19- 847.15)	12.24 (-21.6- 69.79)	-2.37 (-2.76- -1.98)
Burundi	254.26 (139.76- 478.23)	193.92 (121.72- 322.96)	-19.16 (-40.96- 30.68)	-4.25 (-4.68- -3.82)
Cambodia	524.07 (271.78- 966.71)	888.53 (691.74- 1225.99)	149.96 (61.37- 329.3)	-1.01 (-1.09- -0.94)
Cameroon	471.76 (315.28- 644.85)	932.78 (683.65- 1274.37)	35.08 (-8.26- 118.77)	-1.66 (-2.03- -1.29)
Canada	8047.12 (6434.23- 9301.94)	11505.79 (10458.92- 12541.92)	9.54 (-8.71- 42.76)	-1.33 (-1.42- -1.25)
Cape Verde	3.89 (2.95- 5.14)	9.41 (8.13- 10.81)	150.7 (70.78- 258.73)	-0.06 (-0.22- 0.1)
Central African Republic	111.5 (56.3- 219.05)	178.58 (104- 311.6)	16.28 (-15.98- 82.56)	-0.71 (-0.86- -0.56)
Chad	183.48 (84.03- 291.87)	276.7 (157.99- 450.39)	3.15 (-28.45- 58.56)	-1.51 (-1.64- -1.37)
Chile	1060.7 (852.79- 1240.7)	1549.9 (1359.14- 1773.83)	20.46 (-4.3- 60.3)	-0.99 (-1.14- -0.84)
China	38634 (28761.88- 51729.84)	77194.46 (70757.79- 82160.25)	120.79 (53.49- 205.66)	0.67 (0.2- 1.14)
Colombia	1452.01 (1226.78- 1646.08)	2897.07 (2498.93- 3341.84)	83.98 (46.8- 142.77)	-0.96 (-1.15- -0.77)
Comoros	12.61 (7.23- 22.95)	19.39 (13.55- 27.74)	117.29 (43.25- 250.71)	-1.45 (-1.71- -1.19)
Congo	141.47 (78.7- 231.25)	235.57 (164.96- 330.18)	38.83 (-7.92- 118)	-2.01 (-2.34- -1.68)
Costa Rica	81.51 (75.08- 89.02)	182.12 (164.29- 200.25)	38.65 (20.48- 58.24)	-0.95 (-1.38- -0.52)
Cote d'Ivoire	442.75 (270.07- 634.58)	887.67 (609.23- 1231.16)	68.16 (16.05- 162.6)	-1.27 (-1.61- -0.92)
Croatia	503.58 (426.57- 562.24)	1472.65 (1337.47- 1622.37)	254.63 (202.16- 340.02)	5.28 (4.18- 6.4)
Cuba	493.74 (391.8- 575.07)	556.99 (488.71- 635.74)	1.17 (-15.95- 25.7)	-1.25 (-1.53- -0.97)
Cyprus	195.72 (144.83- 260.49)	266.59 (232.31- 310.98)	-5.51 (-35.11- 36.88)	-1.89 (-2.19- -1.58)
Czech Republic	1061.53 (882.75- 1255.14)	1127.98 (1022.22- 1249.65)	25 (0.27- 60.25)	-0.66 (-0.8- -0.53)

© 2021 Zhai Z et al. *JAMA Network Open*.

Democratic Republic of the Congo	869.56 (534- 1475.2)	1588.62 (1091.91- 2418.48)	53.42 (15.31- 114.21)	-0.78 (-0.99- -0.57)
Denmark	1864.79 (1652.71- 2049.71)	2587.72 (2324.73- 2892.02)	51.64 (30.54- 83.96)	-0.05 (-0.16- 0.07)
Djibouti	8.44 (4.76- 14.99)	26.22 (17.46- 41.11)	138.91 (61.45- 282.21)	-0.44 (-0.61- -0.26)
Dominica	3.92 (3.44- 4.51)	7.3 (6.49- 8.14)	71.84 (41.45- 107.67)	1.17 (1.08- 1.26)
Dominican Republic	123.16 (107.45- 142.83)	288.5 (240.41- 343.43)	103.74 (61.93- 153.24)	0.36 (-0.14- 0.86)
Ecuador	287.17 (244.84- 323.67)	729.79 (641.46- 820.32)	97.49 (58.76- 151.9)	0.81 (0.33- 1.3)
Egypt	887.66 (463.8- 1445.42)	4105.33 (2085.13- 6214.44)	384.52 (280.09- 550.86)	3.99 (3.43- 4.56)
El Salvador	143.33 (123.58- 169.38)	235.25 (190.4- 289.37)	91.07 (44.85- 140.56)	-0.37 (-0.6- -0.15)
Equatorial Guinea	18.71 (9.22- 37.88)	19.32 (11.72- 30.89)	4.02 (-49.88- 94.45)	-3.82 (-4.26- -3.37)
Eritrea	117.17 (63.16- 220.57)	212.33 (146.42- 297.44)	262.82 (128.54- 531.43)	-1.13 (-1.21- -1.06)
Estonia	66.87 (60.17- 75.69)	104.23 (88.18- 122.84)	125.44 (80.92- 170.04)	1.06 (0.59- 1.54)
Ethiopia	2237.91 (1089.24- 4496.82)	1601.51 (927.57- 2218.65)	11.3 (-33.8- 142.44)	-4.15 (-4.48- -3.82)
Federated States of Micronesia	5.12 (3.73- 7.52)	8.66 (5.68- 11.58)	92.62 (31.83- 158.78)	0.76 (0.7- 0.83)
Fiji	24.58 (20.46- 29.06)	64.23 (52.93- 76.26)	93.63 (48.64- 145.53)	1.85 (1.35- 2.35)
Finland	1498.12 (1199.73- 1696.93)	1893.53 (1683.95- 2121.62)	29.76 (8.55- 72.57)	-0.64 (-0.86- -0.42)
France	19208.88 (16257.99- 21857.27)	31733.2 (28546.15- 35773.09)	64.35 (35.89- 111.9)	0.48 (0.12- 0.83)
Gabon	41.6 (24.98- 62.5)	62.45 (49.99- 97.97)	39.1 (-10.17- 123.35)	-1.23 (-1.71- -0.75)
Georgia	143.38 (113.03- 164.74)	568.56 (524.06- 612.97)	457.2 (370.65- 600.19)	7.6 (6.28- 8.93)
Germany	24534.51 (20088.67- 27635.63)	29665.78 (26396.25- 33684.38)	27.58 (9.63- 59.7)	-0.49 (-0.61- -0.37)
Ghana	757.16 (607.59- 930.68)	1010.4 (769.53- 1272.54)	13.18 (-22.71- 57.83)	-3.3 (-3.85- -2.75)
Greece	1125.98 (918.6- 1359.17)	1479.82 (1353.33- 1611.26)	21.03 (-1.58- 51.49)	-0.11 (-0.18- -0.04)
Greenland	9.22 (6.29- 11.81)	14.49 (13.01- 16.23)	99.24 (44.6- 203.89)	-0.25 (-0.77- 0.28)
Grenada	9.63 (7.75- 12.44)	9.6 (8.77- 10.46)	-21.37 (-40.65- -2.74)	-1.95 (-2.45- -1.44)
Guam	4.65 (3.86- 5.65)	13.79 (11.91- 16.03)	77.44 (39.76- 128.25)	2.47 (1.83- 3.11)
Guatemala	221.91 (175.37- 298.9)	474.14 (422.99- 530.88)	100.22 (42.37- 164.73)	-1.31 (-1.57- -1.04)

© 2021 Zhai Z et al. *JAMA Network Open*.

Guinea	265.48 (132.71- 402.57)	402.15 (262.11- 624.82)	75.81 (15.18- 203.4)	-0.56 (-0.78- -0.33)
Guinea-Bissau	69.99 (36.49- 119.37)	90.08 (55.21- 145.07)	55.73 (2.24- 168.22)	-1.71 (-1.87- -1.54)
Guyana	55.23 (47.74- 64.06)	68.82 (59.68- 78.36)	46.01 (18.75- 80.58)	-0.59 (-1.1- -0.08)
Haiti	607.17 (284.94- 1104.64)	836.6 (544.14- 1341.31)	44.32 (4.81- 144.54)	-1.31 (-1.55- -1.08)
Honduras	149.3 (111.06- 223.82)	480.9 (355.2- 681.47)	162.53 (67.88- 328.83)	0.96 (0.76- 1.16)
Hungary	922.9 (814.17- 1059.05)	916.47 (842.25- 1002.07)	30.2 (10.77- 51.9)	-0.71 (-0.93- -0.49)
Iceland	43.15 (36.79- 48.29)	89.72 (82.38- 98.13)	72.11 (46.8- 111.66)	0.2 (-0.13- 0.54)
India	26686.66 (16749.85- 47034.23)	67892.25 (57412.77- 87283.23)	183.18 (99.94- 290.46)	0.8 (0.59- 1)
Indonesia	6153.94 (4184.15- 8650.34)	13251.75 (11404.08- 15001.45)	140.17 (85.33- 225.26)	0.84 (0.58- 1.09)
Iran	2145.82 (1698.12- 2931.33)	12579.23 (11438.78- 13555.72)	566.29 (347.18- 789.07)	4.9 (4.05- 5.76)
Iraq	776.59 (514.44- 1094.51)	1258.52 (1118.14- 1405.1)	3.26 (-31.76- 61.74)	-2.85 (-3.68- -2.02)
Ireland	785.1 (521.57- 968.47)	816.89 (729.04- 919.49)	-5.01 (-26.61- 53.41)	-1.97 (-2.12- -1.82)
Israel	612.39 (545.73- 675.89)	880.64 (793.12- 986.48)	-4.07 (-18.38- 16.57)	-1.83 (-2.06- -1.6)
Italy	27183.16 (19897.04- 31894.86)	30174.25 (27334.64- 33365.98)	16.11 (-6.57- 68.34)	-1.03 (-1.09- -0.96)
Jamaica	75.85 (65.58- 86.93)	139.98 (104.49- 173.77)	40.15 (-0.48- 88.42)	-1.06 (-1.93- -0.18)
Japan	12931.08 (11658.06- 13699.94)	26312.81 (24920.3- 28395.15)	72.31 (56.74- 101.97)	1.16 (0.9- 1.42)
Jordan	136.37 (87.32- 187.91)	310.89 (254.18- 369.85)	30.22 (-19.8- 123.57)	-2.49 (-2.89- -2.09)
Kazakhstan	907.2 (801.97- 1080.29)	1153.71 (1063.09- 1249.49)	39.93 (11.61- 64.09)	-0.51 (-1.3- 0.28)
Kenya	331.65 (214.44- 481.09)	929.08 (744.12- 1377.91)	105.84 (68.69- 167)	-0.04 (-0.45- 0.36)
Kiribati	1.63 (1.4- 1.89)	3.87 (3.18- 4.66)	101.03 (54.4- 168.56)	1.33 (1.13- 1.53)
Kuwait	31.95 (29.67- 34.56)	164.26 (137.17- 208.14)	204.71 (153.76- 283.93)	2.48 (1.44- 3.54)
Kyrgyzstan	71.95 (63.35- 81.19)	148.07 (130.93- 175.14)	135.86 (93.31- 204.93)	1.41 (1.08- 1.75)
Laos	250.2 (104.59- 478.59)	314.24 (219.12- 439.02)	91.63 (16.84- 291.31)	-1.9 (-2.04- -1.77)
Latvia	183.03 (161.82- 207.79)	205.44 (179- 237.93)	62.22 (34.73- 90.47)	0.18 (-0.36- 0.72)
Lebanon	71.1 (45.65- 97.13)	194.98 (139.52- 284.86)	100.8 (17.62- 215.22)	0.51 (0.31- 0.71)

© 2021 Zhai Z et al. *JAMA Network Open*.

Lesotho	256.36 (168.08- 360.17)	403.82 (309.37- 535.72)	22.99 (-18.22- 82.13)	1.22 (0.83- 1.61)
Liberia	60.41 (35.22- 91.25)	105.76 (72.1- 163.27)	129.86 (56.36- 261.53)	-0.91 (-1.2- -0.62)
Libya	59.5 (39.65- 81.11)	450.26 (270.05- 739.13)	382.04 (219.39- 622.7)	4.77 (4.42- 5.12)
Lithuania	329.41 (293.97- 372.23)	227.26 (207.7- 248.18)	-17.44 (-29.86- -5.05)	-2.33 (-2.87- -1.8)
Luxembourg	118.3 (92.8- 134.77)	166.51 (141.85- 198.71)	19.69 (-3.72- 73.55)	-0.62 (-0.73- -0.5)
Macedonia	46.69 (37.44- 58.79)	48.95 (42.31- 55.94)	-1.97 (-29.77- 31.67)	-1.86 (-2.25- -1.46)
Madagascar	248.77 (146.43- 450.39)	389.02 (235.24- 665.28)	30.08 (0.04- 71.38)	-1.31 (-1.44- -1.17)
Malawi	165.34 (67.2- 338.33)	257.12 (161.98- 385.37)	134.36 (55.47- 419.32)	-1.45 (-1.97- -0.93)
Malaysia	504.2 (430.42- 625.86)	1334.18 (1137.58- 1565.52)	60.94 (20.86- 101.56)	0.77 (0.61- 0.94)
Maldives	39.95 (16.6- 70)	40.09 (34.11- 48.11)	24.54 (-38.07- 231.21)	-4.42 (-4.7- -4.13)
Mali	416.14 (209.2- 696.96)	362.63 (214.59- 561.46)	-21.81 (-43.5- 18.95)	-3.64 (-4.07- -3.21)
Malta	84.46 (70.87- 95.16)	181.13 (163.49- 199.3)	68.29 (44.35- 103.76)	0.52 (0.36- 0.69)
Marshall Islands	1.55 (0.95- 2.49)	4.14 (2.74- 5.95)	108.47 (58.66- 172.71)	1.18 (0.88- 1.48)
Mauritania	100.63 (65.98- 142.17)	98.69 (78.61- 122.7)	14.8 (-21.26- 78.13)	-3.11 (-3.46- -2.75)
Mauritius	29.18 (24.87- 34.66)	40.64 (36.99- 44.82)	11.73 (-11.92- 34.98)	-0.63 (-1.01- -0.25)
Mexico	3876.07 (3344.98- 4457.49)	10336.56 (9943.82- 10687.14)	111.04 (79.54- 146.78)	0.91 (0.68- 1.14)
Moldova	171.9 (149.91- 201.36)	172.22 (155.57- 191.53)	26.15 (6.52- 47.23)	-1.07 (-1.62- -0.52)
Mongolia	98.67 (69.49- 128.75)	98.63 (83.02- 117.71)	5.74 (-26.98- 63.76)	-4.89 (-5.62- -4.15)
Montenegro	22.69 (20.52- 25.21)	36.72 (30.66- 42.72)	39.78 (15.27- 65.6)	0.44 (0.05- 0.83)
Morocco	356.49 (187.1- 598.27)	1527.76 (969.6- 2172.93)	372.56 (226.64- 585.62)	3.62 (3.26- 3.97)
Mozambique	359.63 (183.9- 701.58)	564.06 (359.01- 949.73)	62.2 (16.83- 152.7)	-0.64 (-0.96- -0.33)
Myanmar	9266.98 (3291.55- 19367.16)	6528.43 (4589.79- 8725.76)	7.81 (-38.16- 134.69)	-3.34 (-3.61- -3.07)
Namibia	188.82 (132.14- 254.28)	248.53 (191.66- 335.8)	4.23 (-28.48- 42.46)	-2.05 (-2.59- -1.5)
Nepal	523.71 (274.35- 1017.2)	944.14 (651.38- 1365.7)	199.2 (92.54- 425.71)	-0.27 (-0.66- 0.13)
Netherlands	8453.83 (7357.15- 9392.2)	11105.64 (10188.88- 12123.9)	20.7 (4.61- 42.7)	-0.96 (-1.15- -0.76)

© 2021 Zhai Z et al. *JAMA Network Open*.

New Zealand	1338.34 (905.76- 1602.15)	1768.82 (1584.51- 1965.6)	15.45 (-7.72- 79.87)	-1.16 (-1.39- -0.93)
Nicaragua	46.4 (37.87- 56.98)	138.21 (115.05- 163.49)	237.92 (140.69- 349.89)	0.65 (0.45- 0.85)
Niger	205.5 (97.95- 337.25)	283.41 (159.38- 472.08)	40.6 (1.48- 102.78)	-2.88 (-3.27- -2.5)
Nigeria	2079.19 (1409.32- 2862.74)	2533.89 (1629.13- 3767.97)	0.92 (-37.67- 62.21)	-2.67 (-2.94- -2.39)
North Korea	606.77 (427.74- 823.43)	1699.77 (1280.18- 2307.68)	87.62 (52.98- 131.67)	2.41 (2.11- 2.71)
Northern Mariana Islands	0.82 (0.64- 1)	1.66 (1.42- 1.89)	50.1 (20.48- 95.26)	0.51 (0.11- 0.9)
Norway	1249.25 (1072.95- 1375.87)	1401.1 (1304.41- 1541.16)	14.98 (-2.23- 46.44)	-0.51 (-0.77- -0.25)
Oman	60.16 (39.76- 94.88)	267.13 (192.96- 354.92)	209.27 (64.27- 430.86)	2.5 (2.12- 2.89)
Pakistan	1940.63 (1230.22- 3149.38)	5720.61 (4264.98- 7652.88)	169.12 (95.72- 288.47)	0.76 (0.39- 1.14)
Palestine	26.56 (17.03- 40.28)	62.62 (54.55- 71.7)	66.97 (-0.64- 176.71)	-1.33 (-1.53- -1.13)
Panama	61.96 (54.75- 68.95)	146.89 (132.81- 161.88)	52.76 (28.26- 81.25)	0.23 (0.03- 0.43)
Papua New Guinea	222.41 (125.24- 406.38)	598.99 (374.74- 1017.11)	47.76 (19.31- 88.68)	0.71 (0.53- 0.89)
Paraguay	94.27 (79.82- 110.09)	300.86 (243.98- 367.7)	120.41 (72.94- 180.51)	1.23 (1.09- 1.37)
Peru	2850.06 (2315.48- 3469.2)	1437.66 (1182.35- 1714.77)	-32.2 (-50.03- -12.67)	-8.14 (-9.37- -6.9)
Philippines	2472.25 (2243.05- 2738.43)	4850.04 (4162.81- 5595.38)	51.96 (28.64- 77.46)	-0.34 (-0.48- -0.2)
Poland	2432.55 (2045.94- 2931.11)	7158.69 (6533.07- 7845.48)	226.42 (157.53- 308.41)	3.71 (3.22- 4.22)
Portugal	906.01 (708.69- 1103.92)	1390.06 (1239.76- 1563.06)	63.93 (26.33- 127.33)	0.11 (-0.42- 0.66)
Puerto Rico	201.05 (178.33- 224.51)	266.7 (239.47- 297.28)	20.64 (0.83- 46.03)	-0.99 (-1.26- -0.72)
Qatar	5.26 (3.83- 7.21)	81.93 (60.97- 112.29)	251.53 (114.74- 492.93)	3.46 (2.89- 4.02)
Romania	1494.22 (1352.04- 1681.58)	1844.4 (1680.22- 2014.43)	50.33 (28.31- 71.16)	0.1 (-0.13- 0.32)
Russian Federation	12472.65 (10565.18- 16236.16)	15424.88 (14698.05- 16228.13)	23.6 (-2.6- 46.02)	-0.28 (-0.8- 0.24)
Rwanda	259.87 (149.17- 481.11)	216.2 (142.57- 307.24)	40.19 (-13.28- 194.75)	-4.33 (-4.96- -3.7)
Saint Lucia	6.9 (5.73- 8.29)	10.86 (9.85- 12.01)	22.35 (-2.92- 50.78)	-1.94 (-2.32- -1.55)
Saint Vincent and the Grenadines	4.29 (3.82- 4.9)	6.91 (6.29- 7.55)	34.94 (12.97- 59.37)	-0.68 (-0.95- -0.41)
Samoa	4.95 (3.95- 6.2)	7.27 (5.6- 9.41)	25.49 (1.9- 57.8)	0.01 (-0.07- 0.08)

© 2021 Zhai Z et al. *JAMA Network Open*.

Sao Tome and Principe	2.32 (1.37- 3.29)	4.92 (3.99- 5.99)	162.61 (77.81- 357.17)	-0.04 (-0.29- 0.2)
Saudi Arabia	101.29 (62.48- 152.81)	611.22 (392.3- 824.19)	413.65 (128.71- 908.92)	2.84 (2.54- 3.14)
Senegal	224.88 (132.14- 320.37)	461.37 (338.12- 622.6)	140.03 (77.54- 255.11)	-0.56 (-0.7- -0.42)
Serbia	698.36 (587.99- 838.97)	935.34 (810.22- 1063.98)	43.79 (18.47- 73.99)	0.61 (0.17- 1.04)
Seychelles	4.2 (2.96- 5.61)	6.53 (5.05- 7.86)	12.85 (-15.11- 41.25)	-1.28 (-1.41- -1.15)
Sierra Leone	98.14 (57.85- 151.22)	234.41 (159.48- 357.59)	155.84 (61.37- 314.53)	0.89 (0.71- 1.07)
Singapore	268.06 (169.19- 352.5)	390.65 (352.37- 432.28)	0.19 (-26.5- 65.91)	-2.49 (-2.73- -2.26)
Slovakia	379.92 (313.28- 459.73)	396.46 (345.18- 453.51)	14.4 (-13.89- 48.23)	-0.91 (-1.06- -0.77)
Slovenia	313.81 (263.07- 362.62)	657.14 (582.56- 745.97)	119.63 (79.29- 182.95)	2.05 (1.37- 2.73)
Solomon Islands	14.85 (9.25- 26.12)	35.45 (24.91- 56)	62.01 (27.27- 117.01)	0.55 (0.42- 0.68)
Somalia	190.97 (74.89- 421.29)	380.31 (208.96- 677.24)	75.23 (15.41- 240.74)	-1.11 (-1.37- -0.85)
South Africa	3809 (3306.24- 4347.24)	7247.87 (6454.34- 8151.49)	39.8 (21.71- 60.41)	-0.5 (-1.31- 0.32)
South Korea	1356.01 (1081.05- 1586.91)	2757.69 (2420.07- 3279.44)	98.58 (53.09- 185.53)	0.46 (0.12- 0.8)
South Sudan	80.11 (28.73- 188.4)	129.91 (65.39- 247.51)	36.48 (-13.41- 157.88)	-0.56 (-0.84- -0.29)
Spain	7341.25 (6198.72- 8444.1)	9562.43 (8732.26- 10501.66)	23.3 (3.47- 54.72)	-0.67 (-0.79- -0.55)
Sri Lanka	1407.26 (1088.82- 1857.81)	2248.46 (1742.97- 2786.97)	69.13 (10.32- 140.71)	-1.07 (-1.47- -0.66)
Sudan	236.5 (108.37- 468.19)	839.51 (530.17- 1290.65)	335.55 (172.18- 661.59)	2.82 (2.53- 3.1)
Suriname	18.7 (16.41- 21.18)	33.26 (29.31- 37.36)	35.79 (12.71- 63.3)	-0.74 (-0.95- -0.52)
Swaziland	67.22 (46.29- 92.44)	122.43 (95.79- 151.95)	25.13 (-13.66- 85.06)	0.23 (-0.33- 0.8)
Sweden	3089.02 (2593.16- 3531.39)	2898.6 (2666.19- 3146.69)	-3.51 (-19.18- 18.85)	-1.22 (-1.34- -1.11)
Switzerland	2557.36 (2117.05- 2862)	3392.23 (3029.54- 3873.22)	31.12 (9.56- 74.48)	-0.13 (-0.4- 0.13)
Syria	235.49 (144.64- 363.02)	545.14 (434.89- 687.7)	47.76 (-15.13- 166.99)	-0.41 (-0.66- -0.16)
Taiwan (China)	615.49 (494.98- 799.79)	1158.12 (1073.16- 1248.17)	42.63 (6.97- 80.59)	-0.36 (-0.64- -0.07)
Tajikistan	290.3 (217.54- 357.96)	651.94 (579.73- 737.69)	99.51 (53.03- 178.22)	0.13 (-0.12- 0.38)
Tanzania	502.3 (264.41- 879.16)	930.41 (597.19- 1302.76)	92 (33.74- 192.64)	-1.2 (-1.54- -0.86)

© 2021 Zhai Z et al. *JAMA Network Open*.

Thailand	7255.43 (6111.49- 8868.56)	7905.65 (6777.18- 9135.14)	-4.9 (-28.12- 18.79)	-3.41 (-4.06- -2.75)
The Bahamas	15.17 (13.03- 17.06)	28.3 (25.4- 31.41)	21.55 (3.35- 43.11)	-0.67 (-0.82- -0.53)
The Gambia	24.22 (14.22- 35.49)	52.72 (39.89- 72.98)	79.37 (21.9- 199.62)	-0.5 (-0.59- -0.41)
Timor-Leste	21.59 (11.92- 38.33)	42.7 (25.77- 56.68)	199.45 (62.94- 503.69)	0.4 (0.08- 0.71)
Togo	123.87 (79.26- 176.51)	235.65 (173.82- 326.85)	72.2 (19.89- 170.52)	-1.75 (-2.02- -1.48)
Tonga	2.52 (2.13- 3.01)	4.15 (3.41- 4.94)	51.56 (21.2- 87.18)	0.87 (0.78- 0.96)
Trinidad and Tobago	39.78 (36- 44.75)	70.6 (57.04- 85.97)	44.75 (19.54- 74.15)	-0.37 (-1.22- 0.49)
Tunisia	65.16 (52.88- 85.13)	339.82 (232.32- 513.59)	408.43 (235.43- 632.8)	3.65 (3.18- 4.12)
Turkey	17263.86 (10014.13- 26376.56)	17606.89 (15576.45- 19884.5)	22.81 (-24.28- 112.65)	-3.24 (-3.71- -2.77)
Turkmenistan	67.34 (56.42- 78.78)	167.89 (143.47- 189.26)	156.13 (115.99- 199.23)	0.86 (0.42- 1.3)
Uganda	247.5 (109.81- 447.97)	431.71 (272.8- 618.79)	123.21 (47.75- 279.75)	-1.29 (-1.56- -1.03)
Ukraine	3800.56 (3173.69- 4579.17)	5834.51 (5393.74- 6269.26)	57.66 (27.18- 93.26)	1.02 (0.57- 1.48)
United Arab Emirates	19.29 (11.14- 30.73)	476.7 (257.09- 826.11)	381.55 (207.11- 637.09)	4.9 (4.52- 5.29)
United Kingdom	43696.85 (33986.95- 51264.33)	48645.34 (46917.87- 50511.22)	15.02 (-4.7- 49.26)	-0.77 (-0.81- -0.72)
United States	47003.3 (42009.41- 50807)	56331.81 (54180.02- 58595.56)	-5.95 (-15.59- 6.05)	-1.53 (-1.69- -1.36)
Uruguay	207.87 (187.6- 229.35)	299.38 (264.92- 336.87)	46.54 (25.94- 69.56)	0.84 (0.53- 1.16)
Uzbekistan	481.97 (392.88- 621.26)	772.87 (674.89- 875.72)	30.68 (0.64- 67.48)	-1.53 (-2.24- -0.82)
Vanuatu	7.37 (4.33- 10.92)	23.08 (13.82- 37.31)	75.71 (32.86- 138.48)	1.38 (1.25- 1.51)
Venezuela	499.44 (449.86- 567.63)	1135.58 (967.05- 1336.9)	47.16 (21.33- 76.07)	-0.92 (-1.19- -0.66)
Vietnam	2375.28 (1804.83- 3203.12)	5245.87 (4398.13- 6297.22)	104.45 (46.92- 171.72)	0.11 (-0.02- 0.24)
Virgin Islands, U.S.	13.9 (12.49- 15.76)	27.65 (22.66- 32.1)	48.69 (26.71- 70.44)	0.69 (0.51- 0.87)
Yemen	195.94 (60.78- 465.82)	936.42 (500.51- 1607.62)	371.57 (192.72- 969.65)	2.71 (2.39- 3.03)
Zambia	216.09 (123.42- 382.96)	421.54 (316.96- 584.82)	100.91 (36.14- 222.7)	-1.5 (-2.11- -0.88)
Zimbabwe	113.5 (81.28- 177.8)	382.94 (306.07- 492.35)	156.35 (80.42- 262.7)	3.35 (2.04- 4.66)

CI: confidence interval; DALY, disability adjusted life-year; EAPC: estimated annual percentage change; UI: uncertainty interval.

© 2021 Zhai Z et al. *JAMA Network Open*.

eFigure 1. Mesothelioma Cases With Age Composition by SDI Region From 1990 to 2017

The data from certain SDI regions are presented in the top-right panel. (A: The cases of incidence; B: The cases of death; C: The number of DALY).

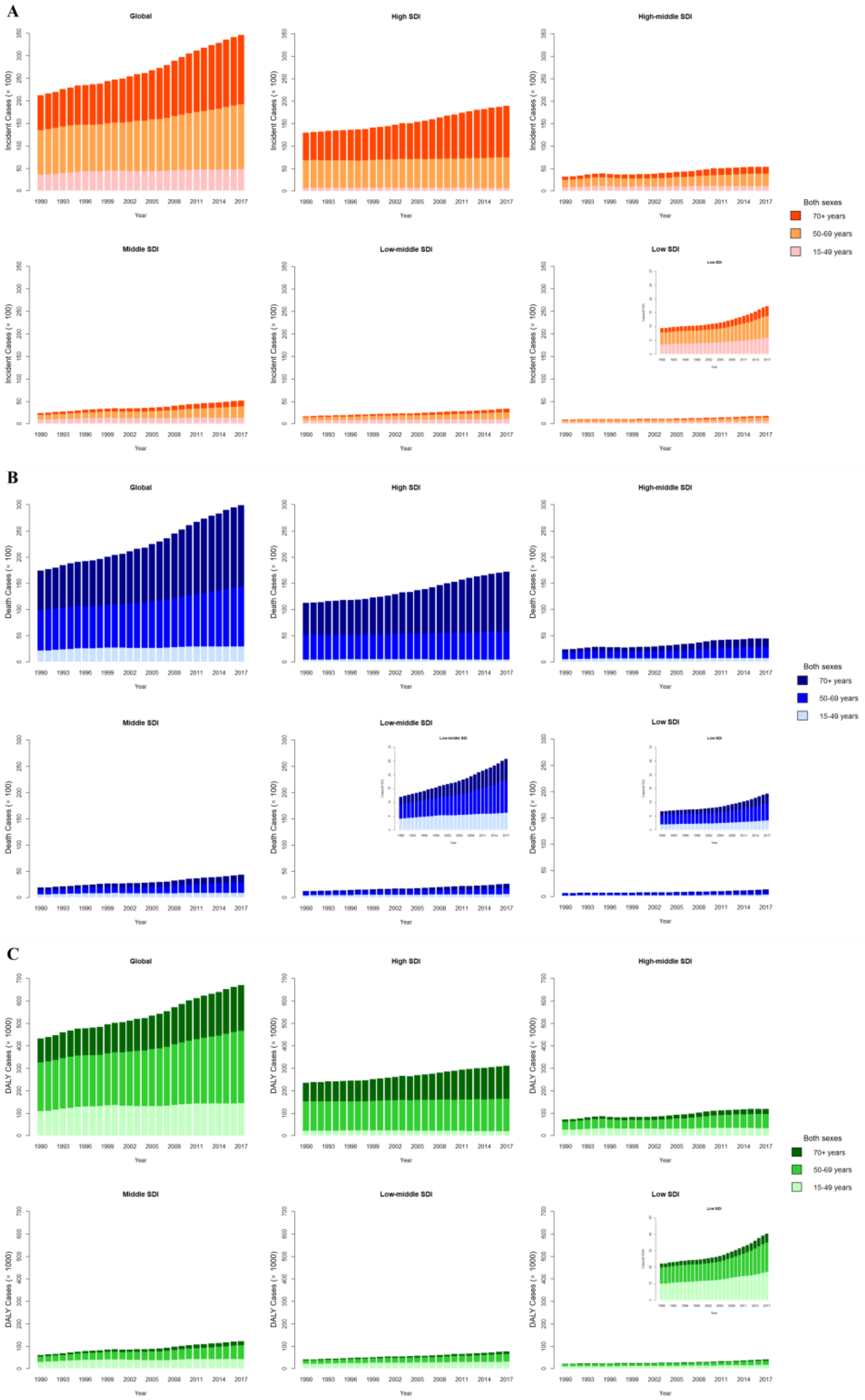

© 2021 Zhai Z et al. *JAMA Network Open*.

eFigure 2. Age-Standardized Incidence Rates for Mesothelioma per 100 000 People in 21 Geographic Regions by SDI in 2017

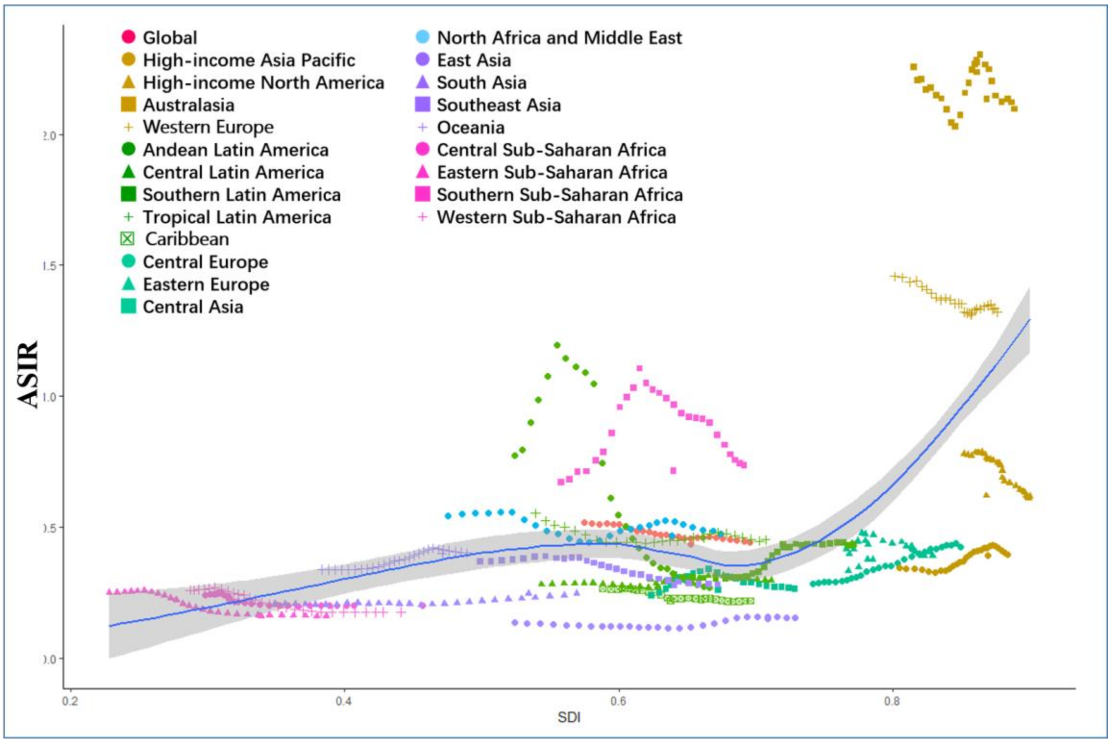

© 2021 Zhai Z et al. *JAMA Network Open*.

eFigure 3. Age-Standardized Death Rates for Mesothelioma per 100 000 People in 21 Geographic Regions by SDI in 2017

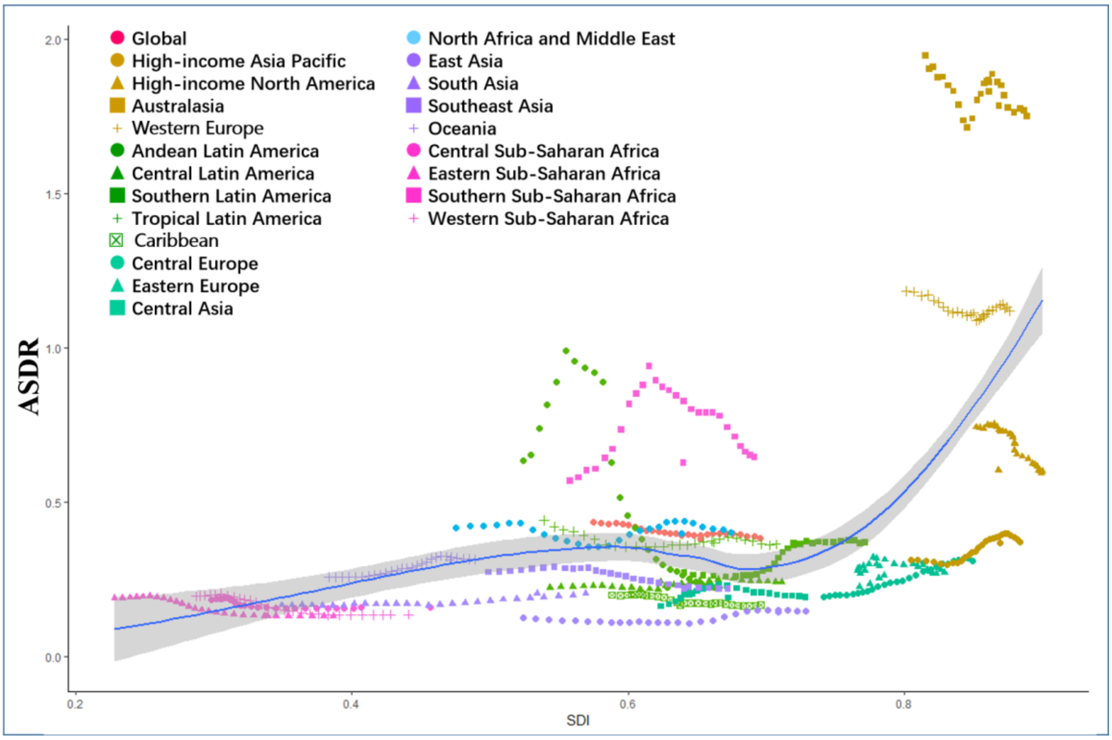

© 2021 Zhai Z et al. *JAMA Network Open*.

eFigure 4. Proportion of Age Subgroups of Incident Cases by Region and Year in 1990 and 2017

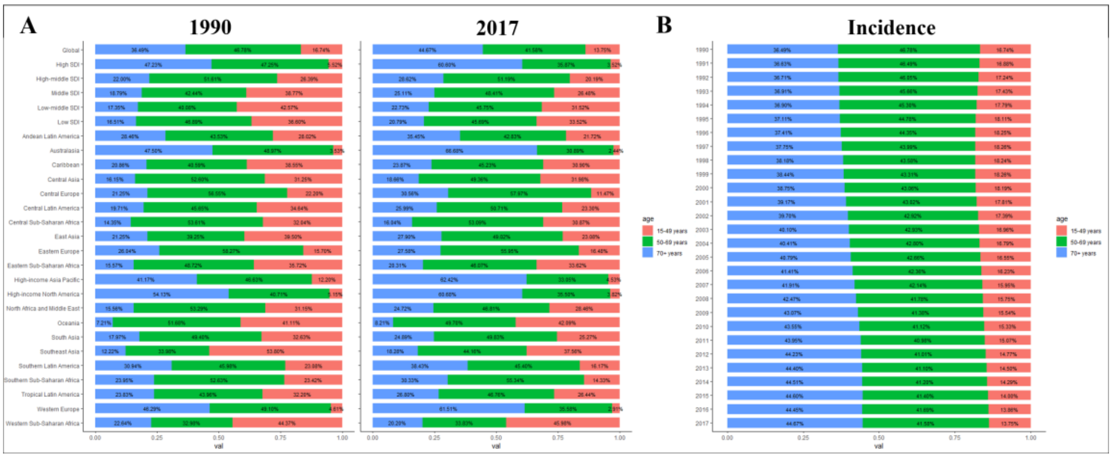

© 2021 Zhai Z et al. *JAMA Network Open*.

eFigure 5. Proportion of Age Subgroups of Incident Cases by Year Among Female and Male Patients

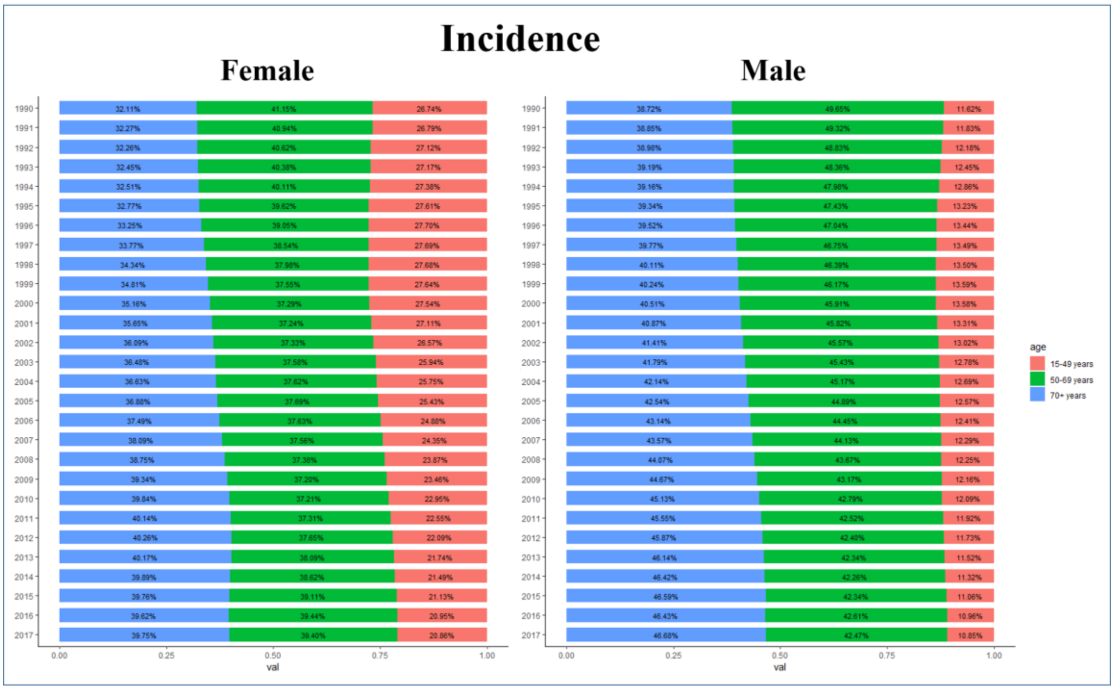

© 2021 Zhai Z et al. *JAMA Network Open*.

eFigure 6. Mesothelioma Cases With Age Composition Among Female Patients by SDI Region From 1990 to 2017

The data from certain SDI regions are presented in the top-right panel. (A: The cases of incidence; B: The cases of death; C: The number of DALY).

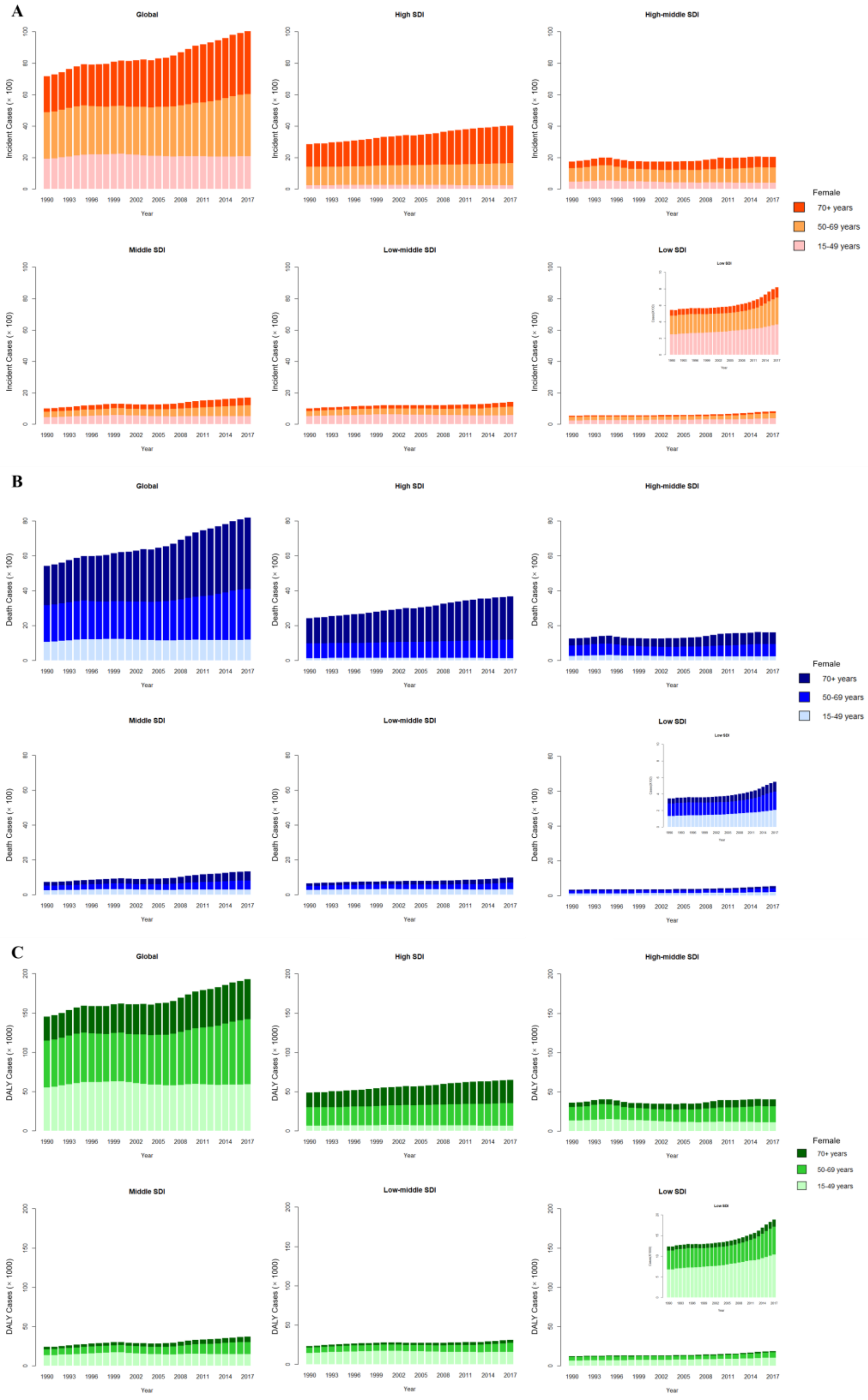

© 2021 Zhai Z et al. *JAMA Network Open*.

eFigure 7. Mesothelioma Cases With Age Composition Among Men by SDI Region From 1990 to 2017

The data from certain SDI regions are presented in the top-right panel. (A: The cases of incidence; B: The cases of death; C: The number of DALY).

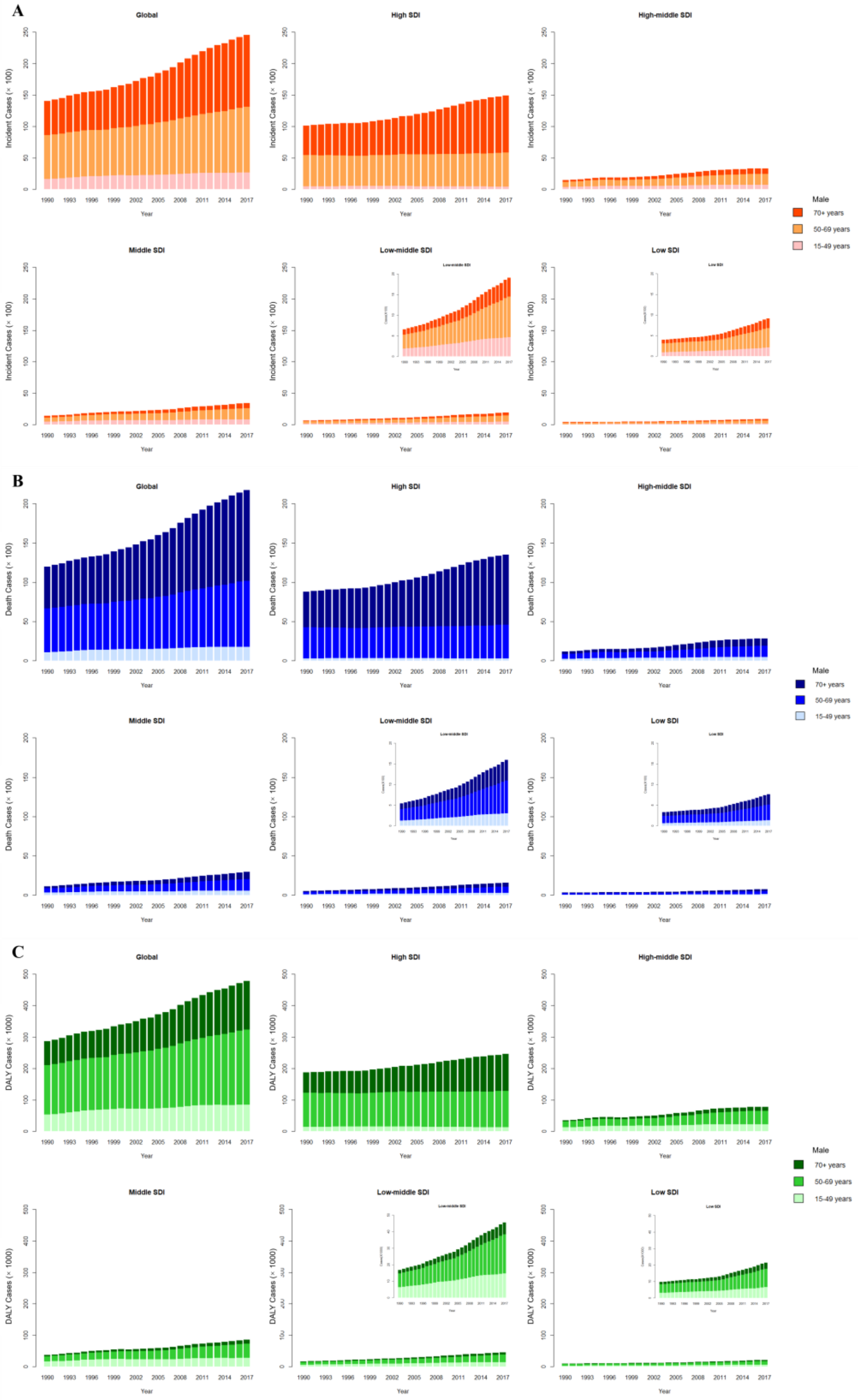

© 2021 Zhai Z et al. *JAMA Network Open*.

eFigure 8. Comparison of Incidence Rates With Age Composition Between Female and Male Patients

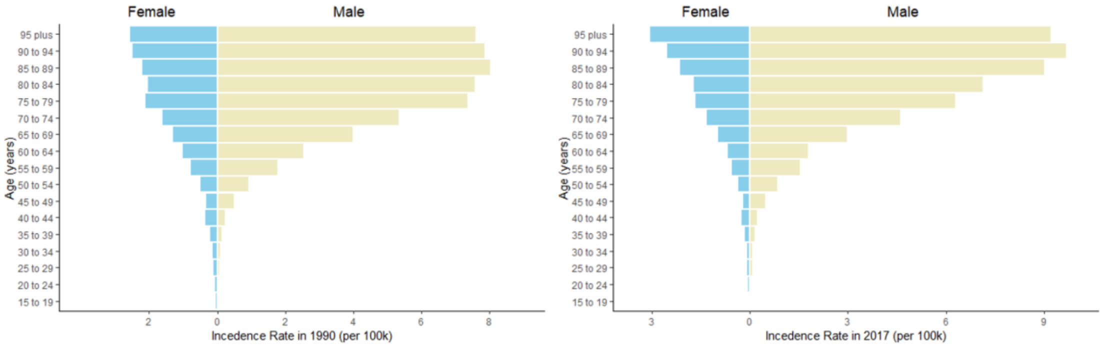

© 2021 Zhai Z et al. *JAMA Network Open*.

eFigure 9. Comparison of Death Rates With Age Composition Between Female and Male Patients

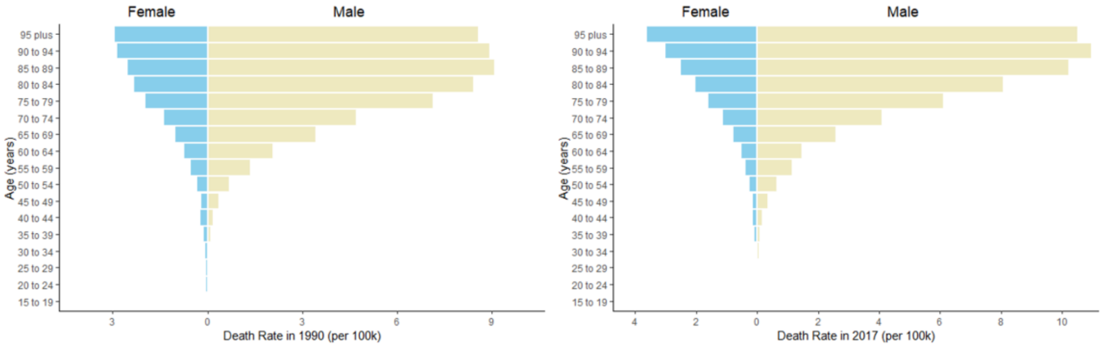

© 2021 Zhai Z et al. *JAMA Network Open*.

eFigure 10. Comparison of DALY Rates With Age Composition Between Female and Male Patients

DALY, disability adjusted life-year.

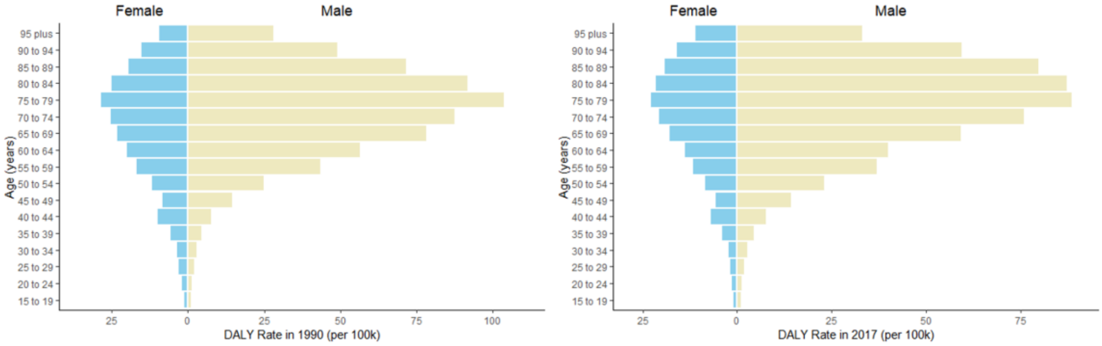

© 2021 Zhai Z et al. *JAMA Network Open*.

eFigure 11. Temporal Trends of Age-Standardized Incidence Rates (ASIRs) for Mesothelioma per 100 000 People in 47 Countries With Complete Asbestos Bans

Symbol of each country was shown in the eTable 1.

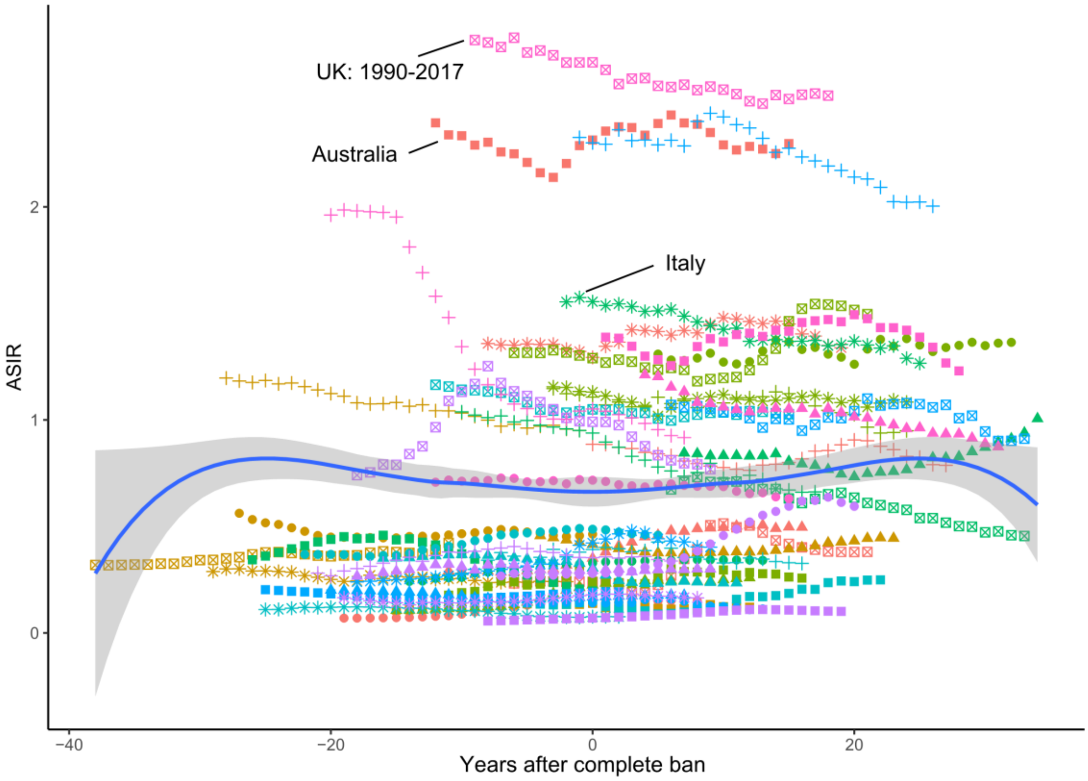

© 2021 Zhai Z et al. *JAMA Network Open*.

eFigure 12. Comparison Between Deaths in 1990 vs 2017 With Age Composition at Regional Level

The left column in each group is case data in 1990 and the right column in 2017. Those data from certain regions can be viewed in the top-right of the panel.

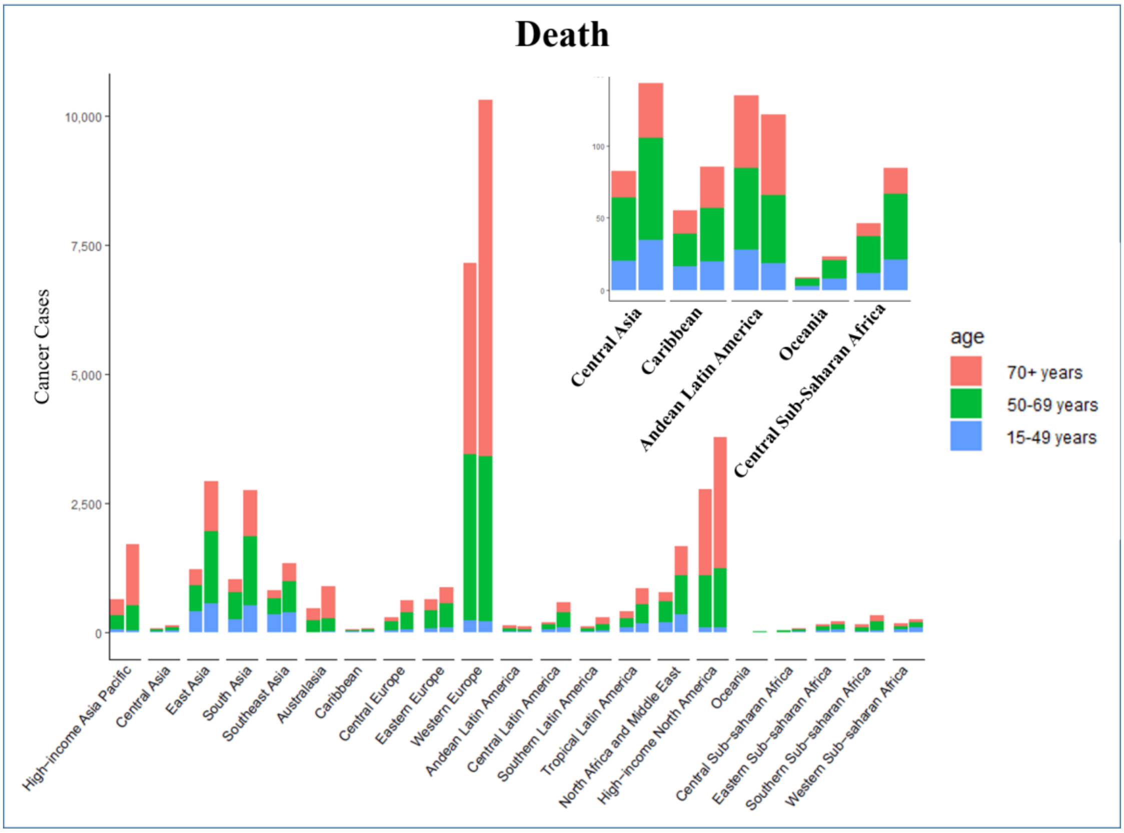

© 2021 Zhai Z et al. *JAMA Network Open*.

eFigure 13. Comparison Between DALYs in 1990 vs 2017 With Age Composition at Regional Level

The left column in each group is case data in 1990 and the right column in 2017. Those data from certain regions can be viewed in the top-right of the panel. DALY, disability adjusted life-year.

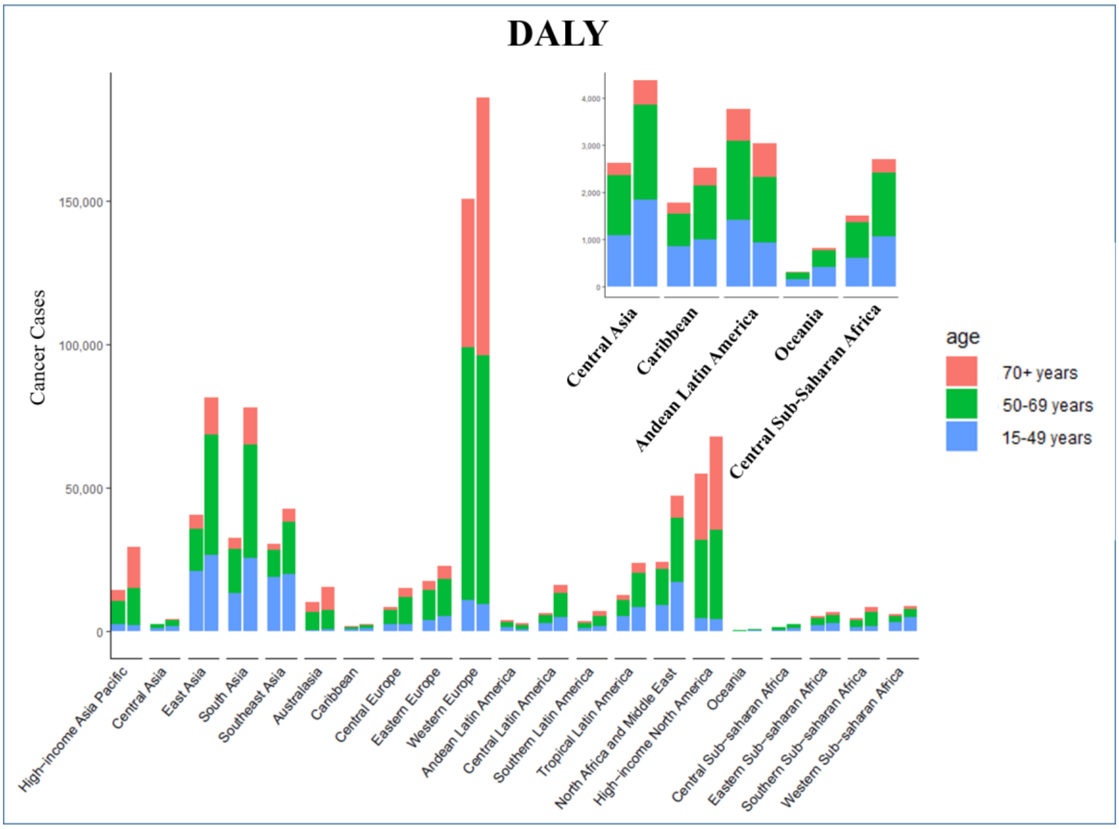

© 2021 Zhai Z et al. *JAMA Network Open*.

eFigure 14. Proportion of Deaths in Age Subgroups by Region and Year

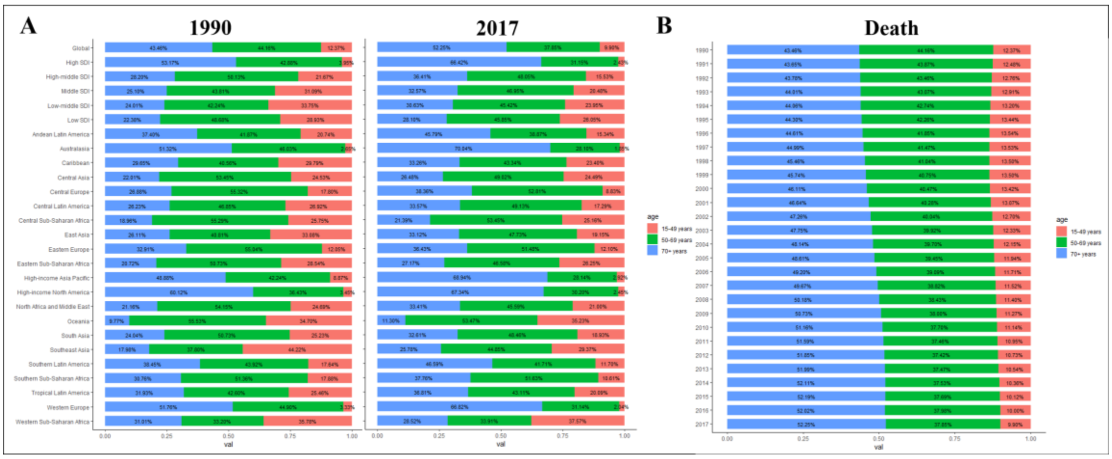

© 2021 Zhai Z et al. *JAMA Network Open*.

eFigure 15. Age-Standardized DALY Rates for Mesothelioma per 100 000 People in 21 Geographic Regions by SDI in 2017

DALY, disability adjusted life-year.

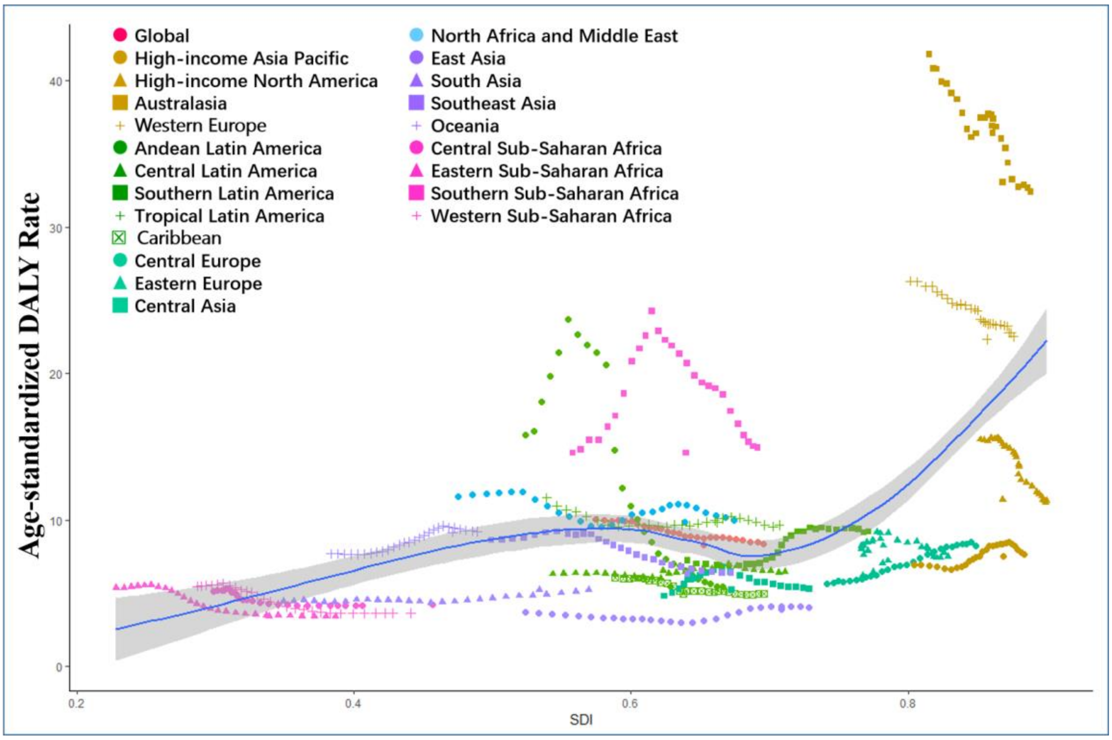

© 2021 Zhai Z et al. *JAMA Network Open*.

eFigure 16. Temporal Trends of Age-Standardized Incidence, Death, and DALY Rates for Mesothelioma by SDI Level Among Female and Male Patients

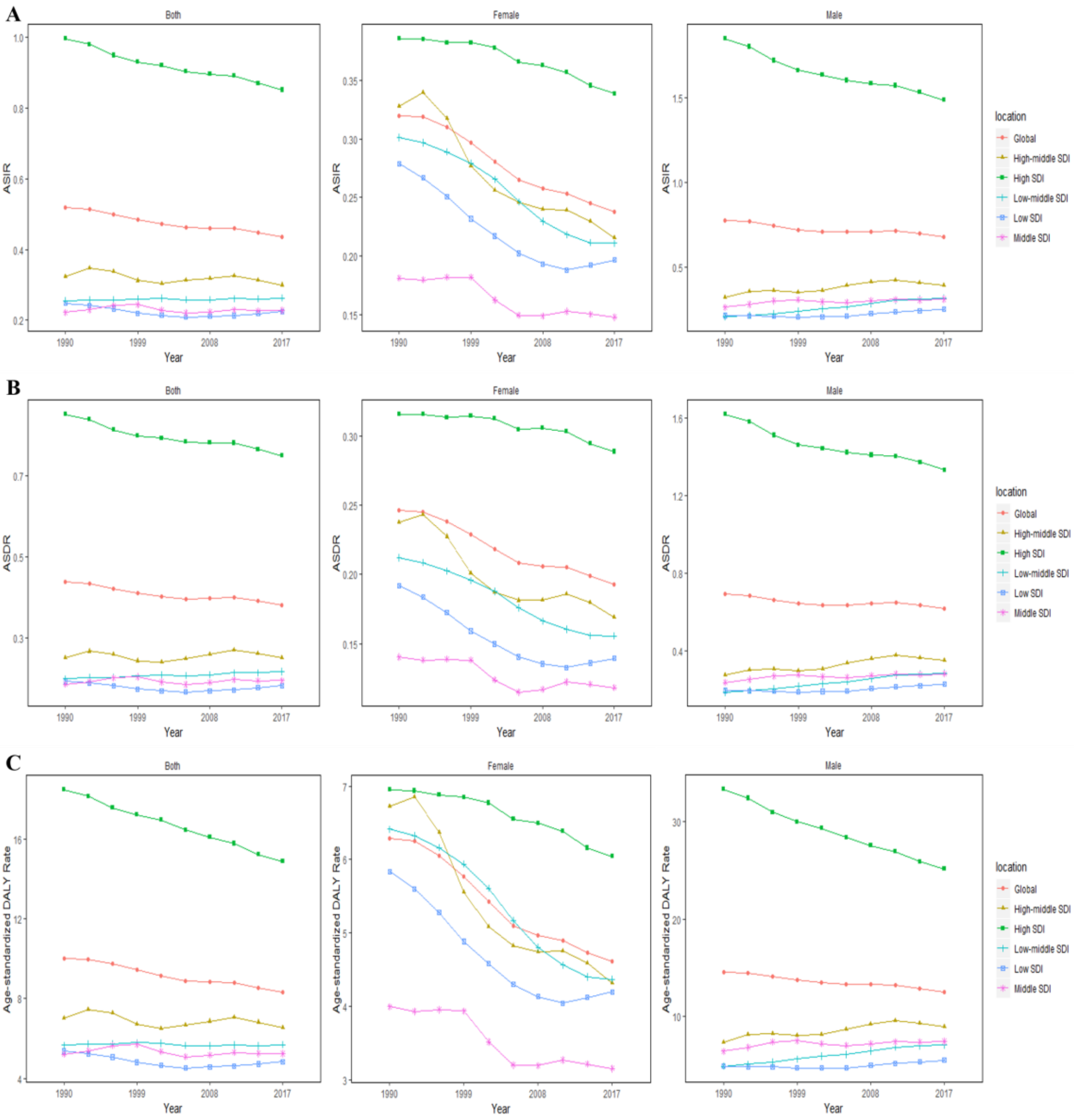

© 2021 Zhai Z et al. *JAMA Network Open*.

eFigure 17. Joinpoint Regression Analysis of Age-Standardized Incidence Rates (ASIRs) Among Female Patients in SDI Regions From 1990 to 2017

APC: annual percent change.

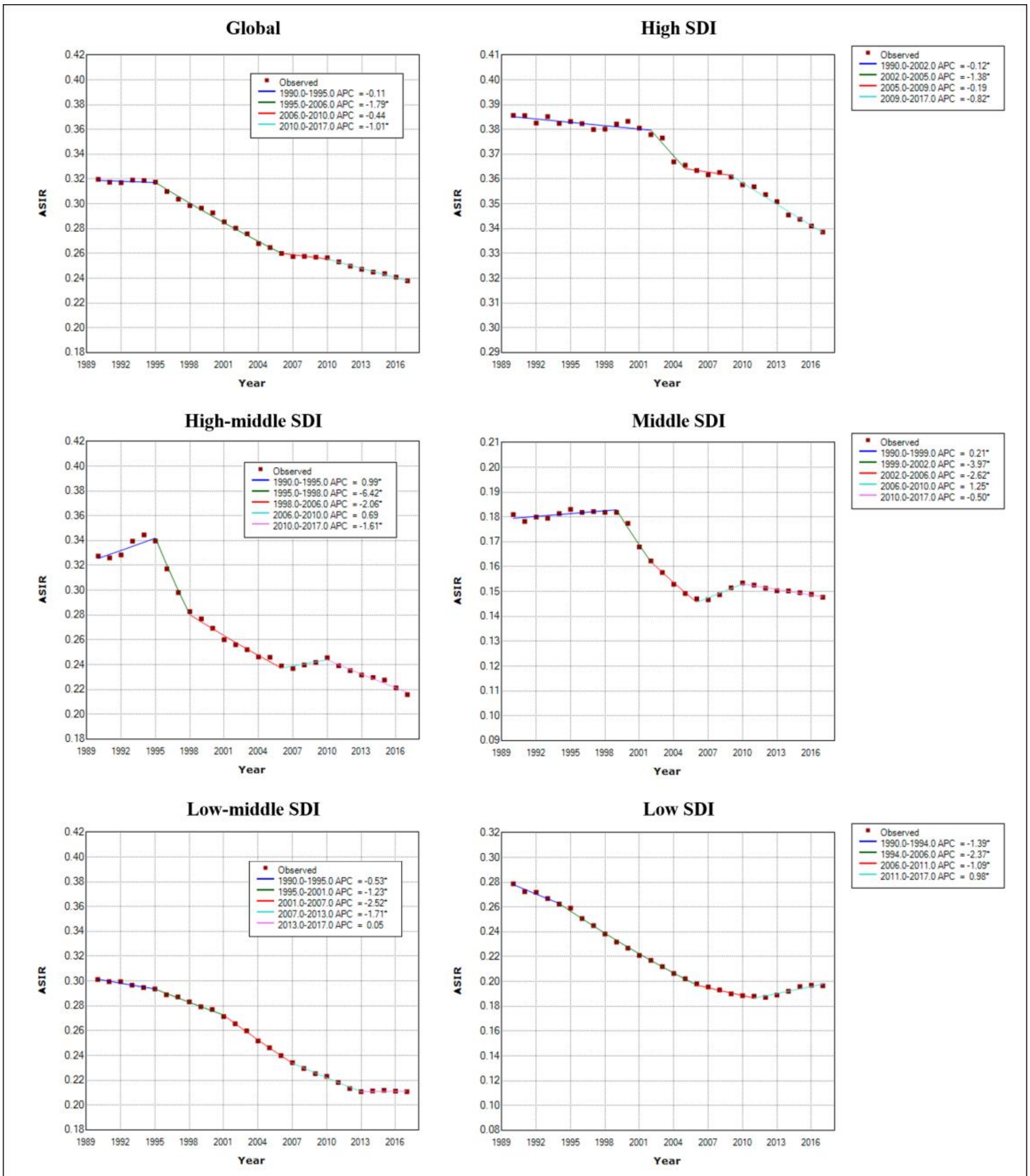

* Indicates that the Annual Percent Change (APC) is significantly different from zero at the alpha = 0.05 level.

© 2021 Zhai Z et al. *JAMA Network Open*.

eFigure 18. Joinpoint Regression Analysis of Age-Standardized Incidence Rates (ASIRs) Among Male Patients in SDI Regions From 1990 to 2017

APC: annual percent change.

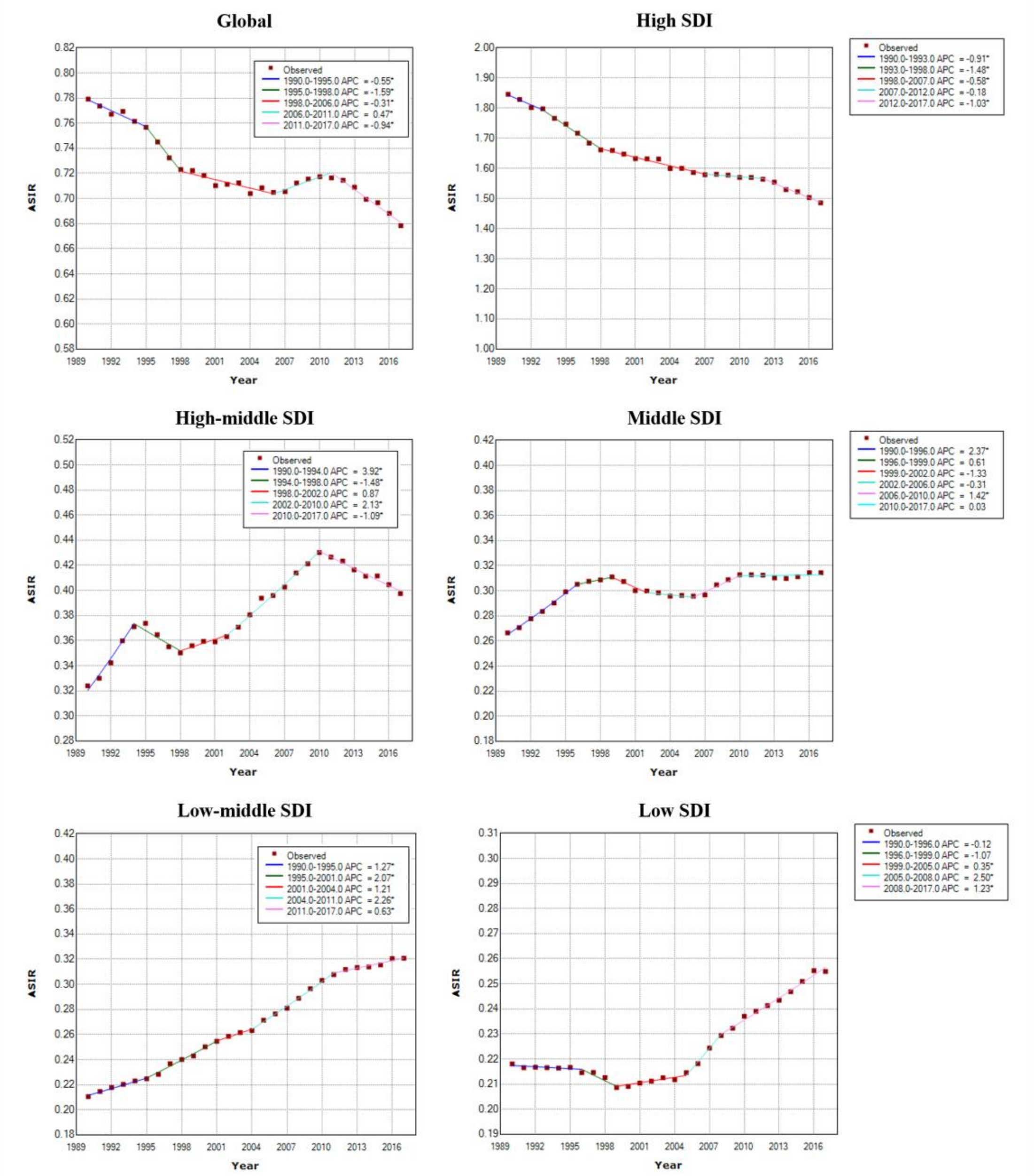

* Indicates that the Annual Percent Change (APC) is significantly different from zero at the alpha = 0.05 level.

© 2021 Zhai Z et al. *JAMA Network Open*.

eFigure 19. Joinpoint Regression Analysis of Age-Standardized Incidence Rates (ASIRs) in SDI Regions From 1990 to 2017

APC: annual percent change.

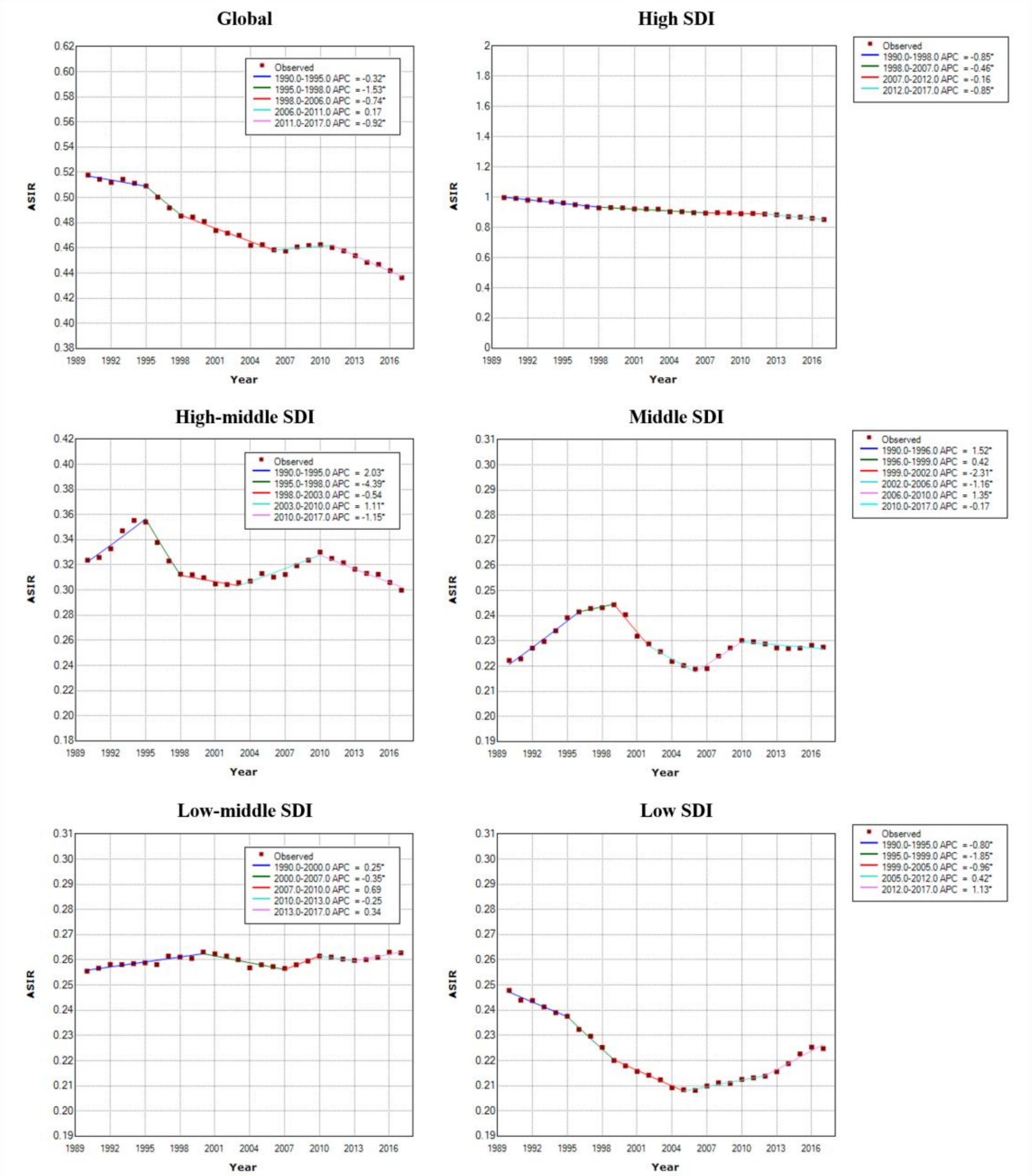

* Indicates that the Annual Percent Change (APC) is significantly different from zero at the alpha = 0.05 level.

© 2021 Zhai Z et al. *JAMA Network Open*.

eFigure 20. Joinpoint Regression Analysis of Age-Standardized Death Rates (ASDRs) in SDI Regions From 1990 to 2017

APC: annual percent change.

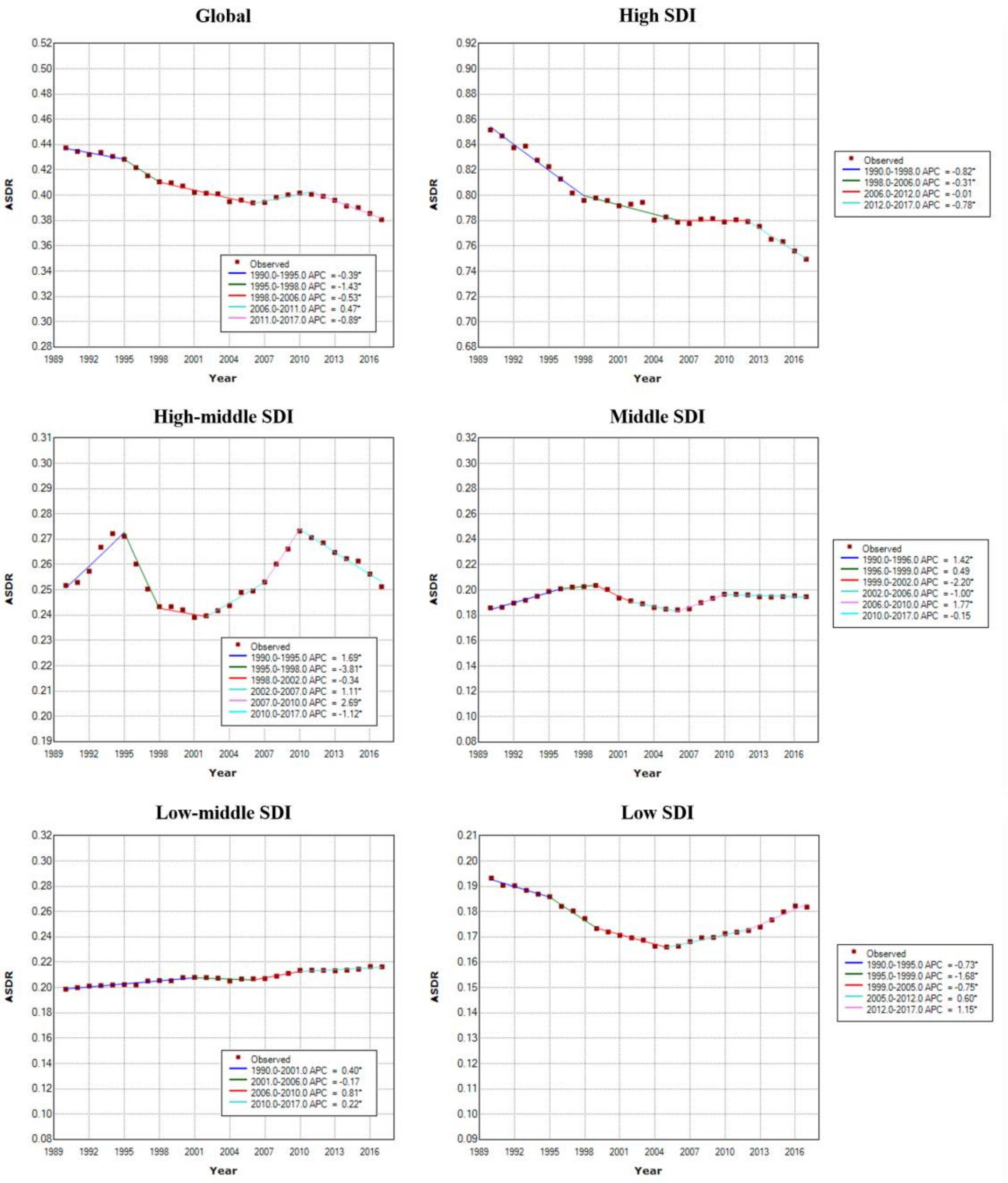

* Indicates that the Annual Percent Change (APC) is significantly different from zero at the alpha = 0.05 level.

© 2021 Zhai Z et al. *JAMA Network Open*.

eFigure 21. Epidemiological Patterns of Mesothelioma Deaths in 195 Countries and Territories

(A) ASDR of mesothelioma in 2017; (B) All ages percent change in death cases between 1990 and 2017; (C) The EAPC of ASDR from 1990 to 2017. Countries with an extreme value of cases' number or change were annotated. ASDR, age-standardized death rate; EAPC, estimated annual percentage change.

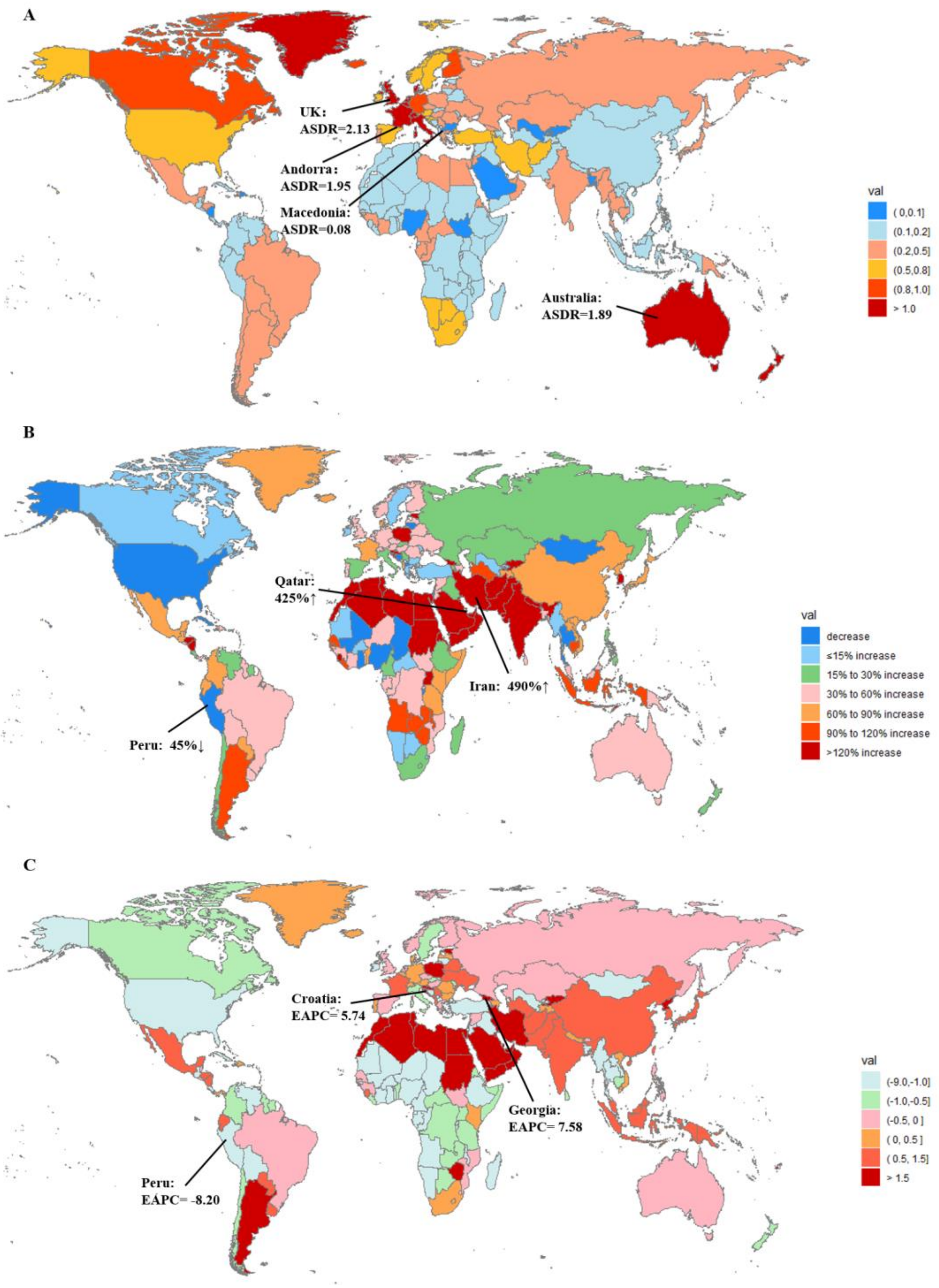

© 2021 Zhai Z et al. *JAMA Network Open*.

eFigure 22. Epidemiological Patterns of Mesothelioma DALYs in 195 Countries and Territories

(A) ASR of DALY in 2017; (B) All ages percent change in DALY cases between 1990 and 2017; (C) The EAPC of ASR from 1990 to 2017. Countries with an extreme value of cases' number or change were annotated. DALY, disability adjusted life-year; EAPC, estimated annual percentage change.

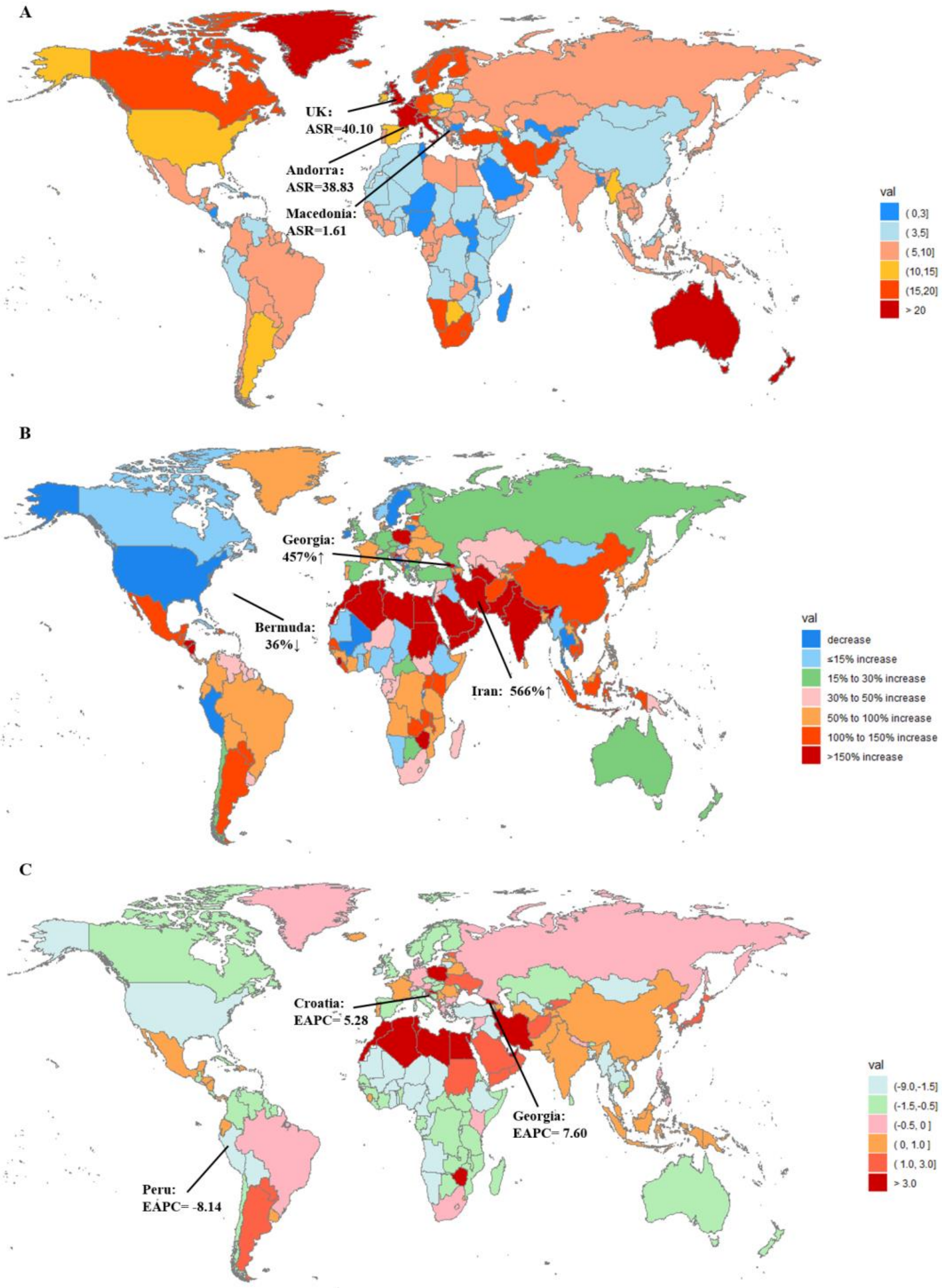

© 2021 Zhai Z et al. *JAMA Network Open*.

eFigure 23. Epidemiological Patterns Among Male Patients in 195 Countries and Territories

(A) ASIR of the male in 2017; (B) ASDR of the male in 2017; (C) The EAPC of ASIR from 1990 to 2017. Countries with an extreme value of ASR or change were annotated. ASR, age-standardized rate; ASIR, age-standardized incidence rate; ASDR, age-standardized death rate; EAPC, estimated annual percentage change.

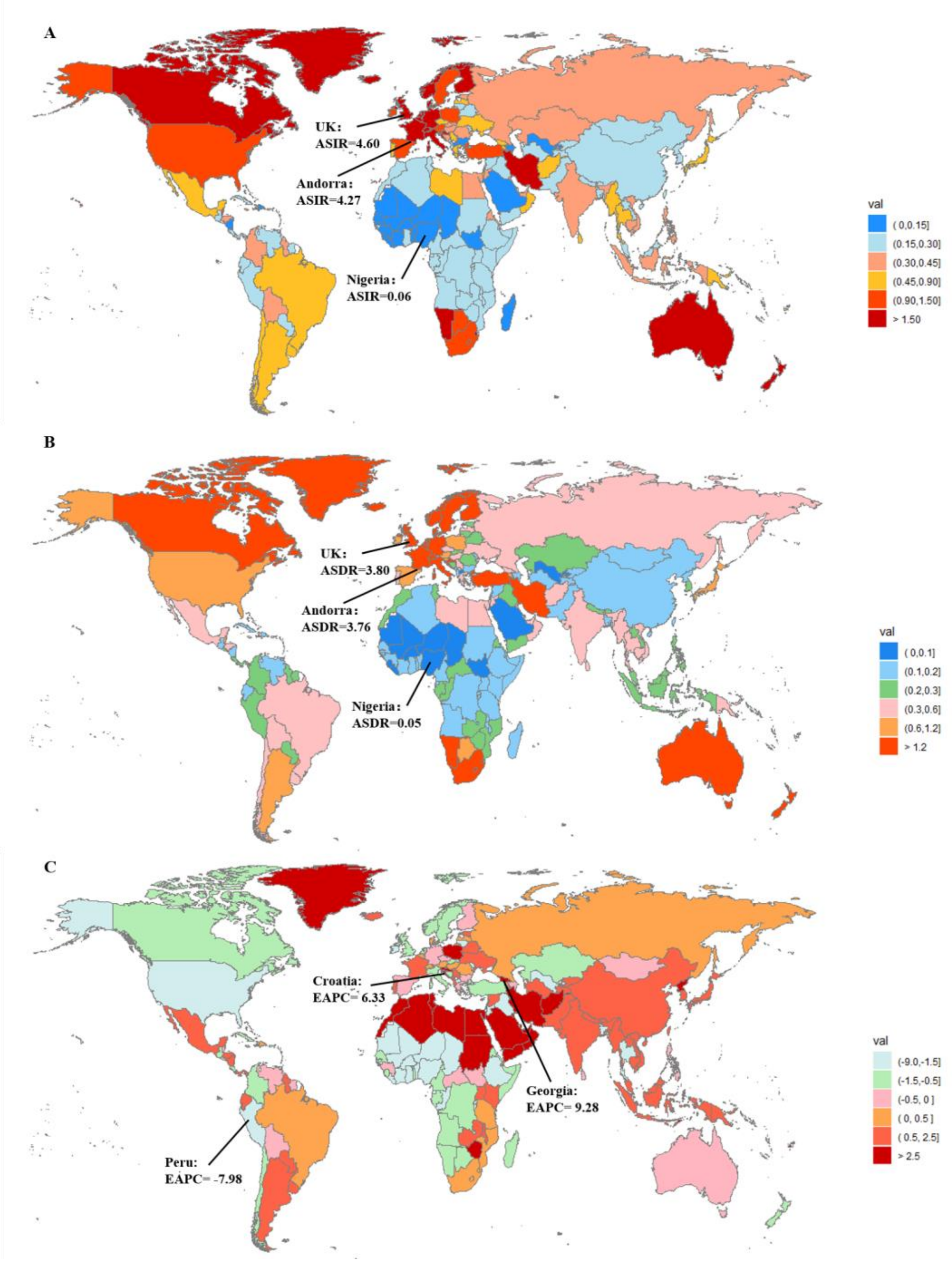

© 2021 Zhai Z et al. *JAMA Network Open*.

eFigure 24. Epidemiological Patterns Among Female Patients in 195 Countries and Territories

(A) ASIR of the female in 2017; (B) ASDR of the female in 2017; (C) The EAPC of ASIR from 1990 to 2017. Countries with an extreme value of ASR or change were annotated. ASR, age-standardized rate; ASIR, age-standardized incidence rate; ASDR, age-standardized death rate; EAPC, estimated annual percentage change.

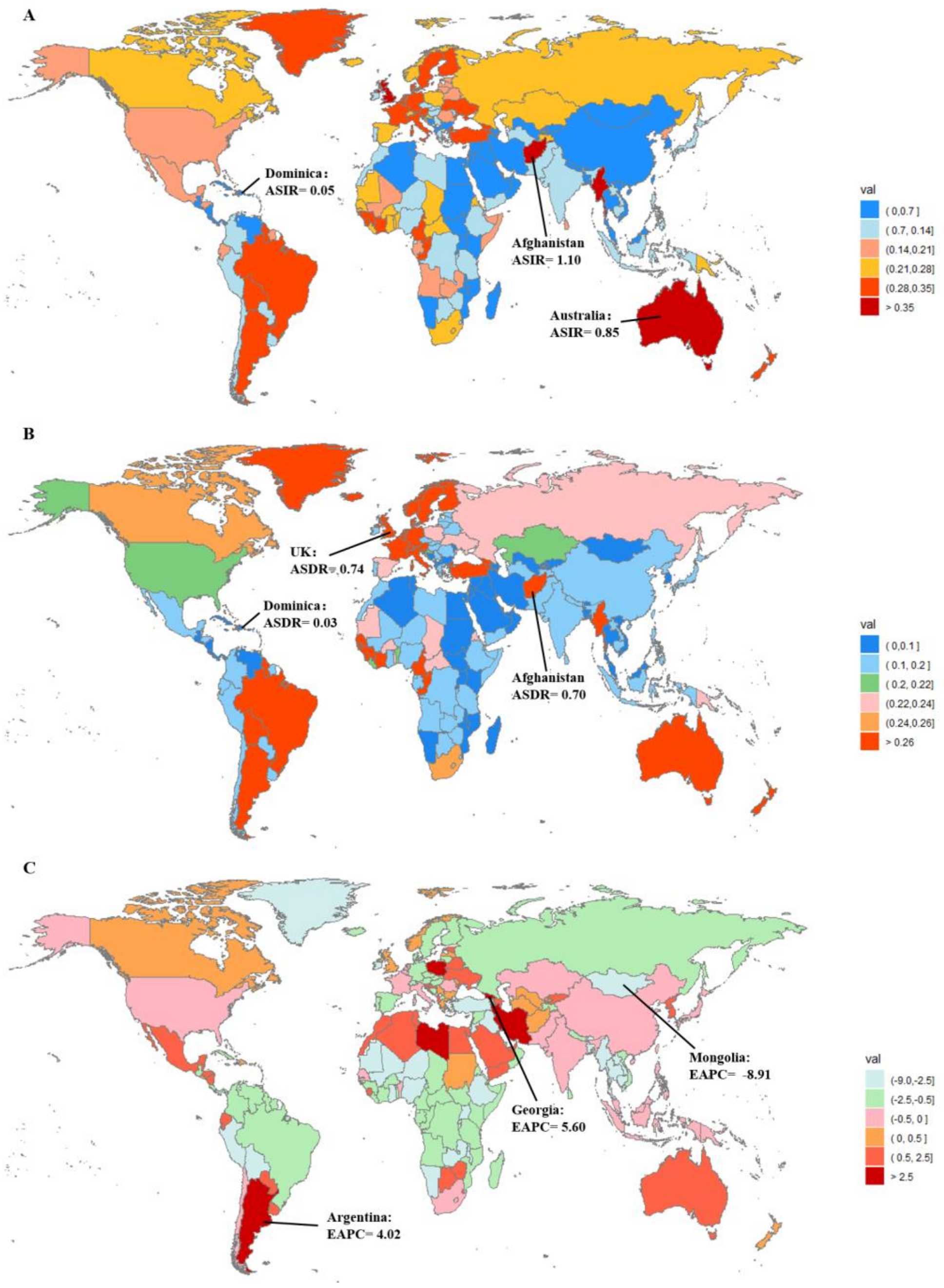

© 2021 Zhai Z et al. *JAMA Network Open*.

eReferences

1. Liu Z, Jiang Y, Yuan H, Fang Q, Cai N, Suo C, et al. The trends in incidence of primary liver cancer caused by specific etiologies: Results from the Global Burden of Disease Study 2016 and implications for liver cancer prevention. Journal of hepatology. 2019;70(4):674-83.
2. Roth GA, Abate D, Abate KH, Abay SM, Abbafati C, Abbasi N, et al. Global, regional, and national age-sex-specific mortality for 282 causes of death in 195 countries and territories, 1980–2017: a systematic analysis for the Global Burden of Disease Study 2017. The Lancet. 2018;392(10159):1736-88.
3. James SL, Abate D, Abate KH, Abay SM, Abbafati C, Abbasi N, et al. Global, regional, and national incidence, prevalence, and years lived with disability for 354 diseases and injuries for 195 countries and territories, 1990–2017: a systematic analysis for the Global Burden of Disease Study 2017. The Lancet. 2018;392(10159):1789-858.
4. Kim HJ, Fay MP, Feuer EJ, Midthune DN. Permutation tests for joinpoint regression with applications to cancer rates. Statistics in medicine. 2000;19(3):335-51.
5. Network. GBoDC. Global Burden of Disease Study 2017 (GBD 2017) Socio-Demographic Index (SDI) 1950–2017. 2018 ed2018.

© 2021 Zhai Z et al. *JAMA Network Open*.
